# Degradation by Design: New Cyclin K Degraders from Old CDK Inhibitors

**DOI:** 10.1021/acschembio.3c00616

**Published:** 2024-01-09

**Authors:** Katie L. Thomas, Habib Bouguenina, Daniel S. J. Miller, Fernando J. Sialana, Thomas G. Hayhow, Jyoti S. Choudhary, Olivia W. Rossanese, Benjamin R. Bellenie

**Affiliations:** † Centre for Cancer Drug Discovery, The Institute of Cancer Research, London SM2 5NG, U.K.; ‡ Functional Proteomics Group, The Institute of Cancer Research, London SW3 6JB, U.K.; § Oncology R&D, AstraZeneca, 1 Francis Crick Avenue, Cambridge Biomedical Campus, Cambridge CB2 0AA, U.K.

## Abstract

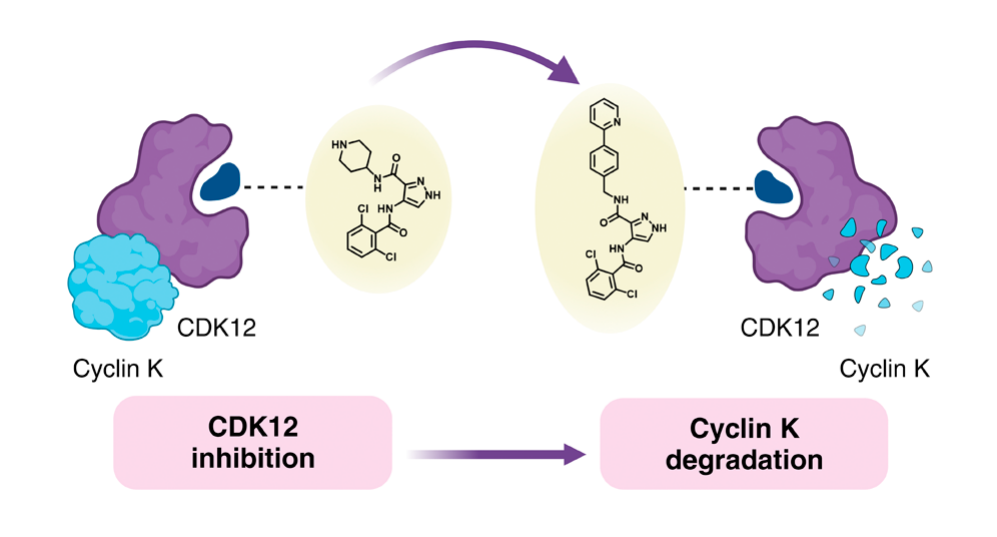

Small molecules that induce protein degradation hold the potential to overcome several limitations of the currently available inhibitors. Monovalent or molecular glue degraders, in particular, enable the benefits of protein degradation without the disadvantages of high molecular weight and the resulting challenge in drug development that are associated with bivalent molecules like Proteolysis Targeting Chimeras. One key challenge in designing monovalent degraders is how to build in the degrader activity—how can we convert an inhibitor into a degrader? If degradation activity requires very specific molecular features, it will be difficult to find new degraders and challenging to optimize those degraders toward drugs. Herein, we demonstrate that an unexpectedly wide range of modifications to the degradation-inducing group of the cyclin K degrader CR8 are tolerated, including both aromatic and nonaromatic groups. We used these findings to convert the pan-CDK inhibitors dinaciclib and AT-7519 to Cyclin K degraders, leading to a novel dinaciclib-based compound with improved degradation activity compared to CR8 and confirm the mechanism of degradation. These results suggest that general design principles can be generated for the development and optimization of monovalent degraders.

## Introduction

In recent years, there have been significant developments in the modalities available to target protein function for therapeutic benefit.^1,2^ Targeted protein degradation (TPD) represents one of these innovative strategies that relies on hijacking the endogenous protein recycling machinery of the cell, known as the ubiquitin-proteasome system.^3,4^ Small molecules are used to induce protein–protein interactions (PPIs) between an E3 ligase and a target protein of interest, leading to polyubiquitination and subsequent degradation of the target protein by the proteasome.^5,6^ This strategy presents a range of advantages over classical inhibition as it has the ability to abrogate scaffolding functions and can induce longer-lasting effects at lower treatment concentrations due to the catalytic mechanism of action.^7,8^ Furthermore, inhibitor development has been limited to the subset of proteins that possess a ligandable active site, with the majority of the proteome being considered “undruggable”.^9^

Within the TPD space, Proteolysis Targeting Chimeras (PROTACs) have seen significant advances in their design principles.^10−12^ These bivalent compounds combine a target binder with an E3 ligase binder through a linker to induce ternary complex formation and subsequent degradation. This strategy has proven powerful, with over 15 compounds entering clinical trials since 2019 for a range of targets such as BTK, AR, and IRAK4.^13^ Due to their modular nature, PROTACs can be readily constructed by combining a target binder with a known E3 ligase ligand and optimizing the linker. Unfortunately, despite their readiness for design, PROTACs can be challenging to develop for clinical use as their large size often results in unfavorable pharmacokinetic properties.^14^ As an alternative, monovalent degraders that act at the interface of two proteins are being explored, as they are often smaller in size (<500 Da).^15−17^ These compounds often possess a strong affinity for only one protein partner, and the combined protein-degrader surface can form new or stabilize existing PPIs. Unlike PROTACs, these compounds initially bind to one substrate and, therefore, are not subject to a “hook effect” and maintain their degradation effect at higher concentrations. Compounds that can induce these PPIs are frequently termed molecular glues, although the mechanism of action is often elucidated retrospectively. This is the case for several approved anticancer drugs, such as the immunomodulatory imide drug (IMiD) thalidomide.^18,19^ Using a TPD approach can enable the targeting of proteins that do not possess a ligandable binding site, such as transcription factors, by binding to an E3 ubiquitin ligase and altering its substrate selectivity.^20^ Despite the promise of molecular glues, designing compounds to degrade a specific target of interest is not currently deemed possible, and further work is required to elucidate the structure-activity relationship (SAR) around ligase/target recruitment. Previous work by our group on BCL6 degraders showed sharp drop-offs in degradation ability with small changes to the solvent-exposed portion of the molecule; for example, switching from a difluoropiperidine to a morpholine ring abrogated degradation.^21^ This has also been seen for IMiDs; for example, lenalidomide, which lacks a carbonyl group in the phthalimide ring compared to the IMiD pomalidomide, can also recruit an additional nonzinc finger neo-substrate, casein kinase I isoform alpha (CK1α), to cereblon for ubiquitination.^22^ Thus, early evidence suggests that the PPIs induced by molecular glues may be highly sensitive to the structural features of the glue. This makes the optimization of monovalent degraders from hit compounds into drug candidates challenging, adding further complexity to the development of this modality.

In 2020, Słabicki et al. revealed that the pan-CDK inhibitor CR8 was acting as a monovalent degrader of cyclin K.^23^ CR8 binds to cyclin-dependent kinase 12 (CDK12) and induces its association with the E3 ligase adaptor protein damaged DNA binding protein 1 (DDB1). This binding brings the CDK12 partner protein, cyclin K, within proximity of the E3 ligase, leading to its ubiquitination and subsequent degradation. The study also provided detailed crystal structures of this novel complex, revealing how the neosurface created by CDK12 and the solvent-exposed pyridyl group of CR8 interact with the DDB1 β-propeller C (BPC) domain, leading to a strong stabilization of the interaction (1000-fold compared to apo-form in vitro). Based on these detailed insights, we explored the SAR of cyclin K degraders to understand the determinants of DDB1 recruitment and extract key features of the molecules that drive degradation. We translated the findings from this SAR to other pan-CDK inhibitors and showed that these inhibitors can be converted into potent cyclin K degraders through the incorporation of solvent-exposed groups. By providing novel insights on the SAR of a known monovalent degrader and translating them to close relatives, our work sheds new light on the potential of converting small molecule inhibitors to degraders and is a step toward the rational design of novel monovalent degraders.

## Results and Discussion

**Figure 1:**
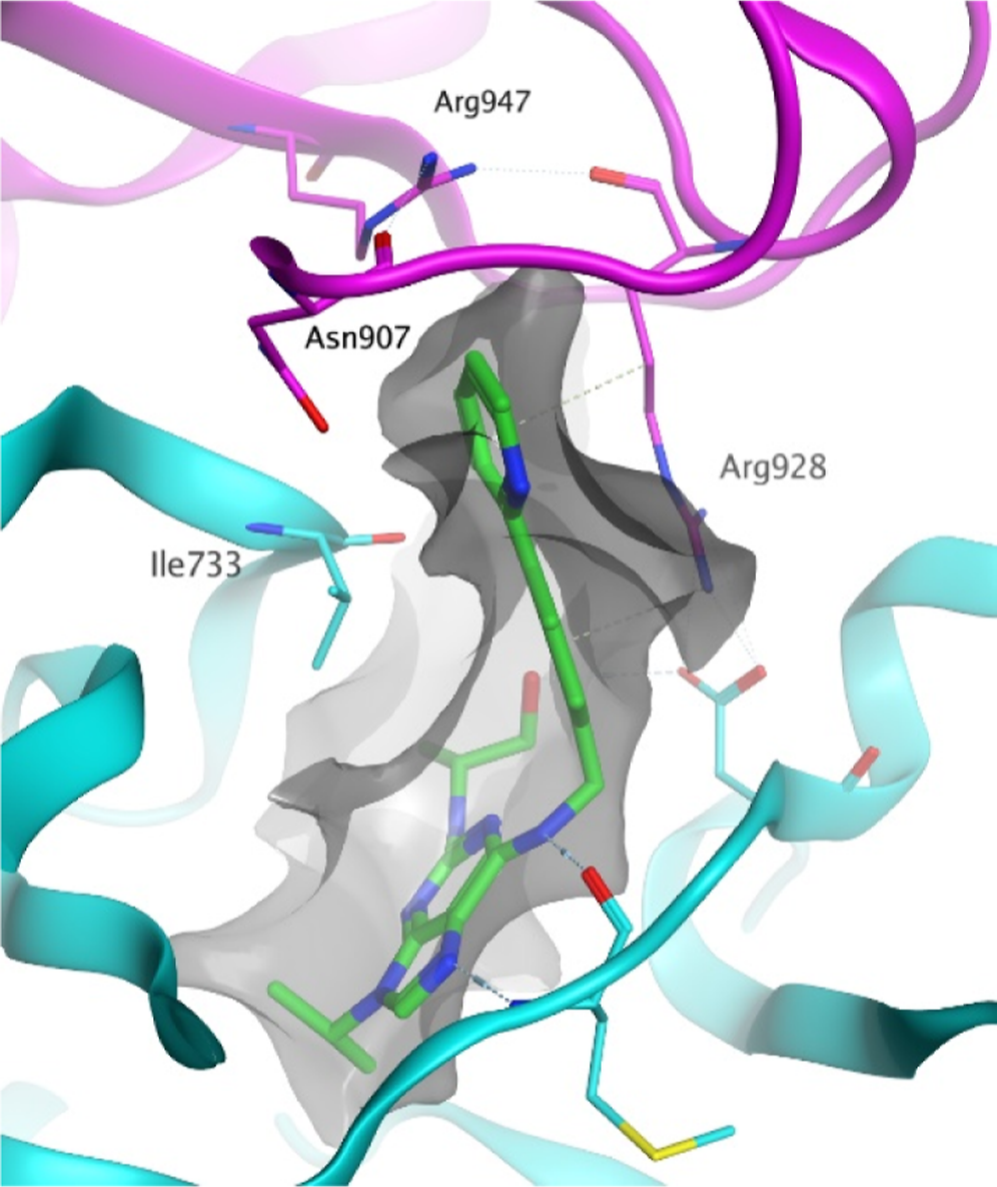
Crystal structure of CR8-bound CDK12 in complex with DDB1 and cyclin K (PDB: 6TD3). A narrow pocket formed by DDB1 is occupied by a planar pyridyl group. DDB1 is shown in pink, CDK12 is shown in blue, and the gray mesh shows the interaction map.

CR8 (**1**) was developed from seliciclib (**SCB**, **2**), a small-molecule CDK inhibitor that lacks solvent-exposed pyridine (Table 1).^24^ The subtle difference between the inhibitor and degrader demonstrates that small surface modifications can greatly impact the mechanism of action of a compound. This system is ideal to study the SAR of degradation through alterations to the pyridine ring to see whether degradation ability can be maintained or improved with other groups. We first sought to explore small structural changes to the solvent-exposed pyridine ring in order to determine how broad the SAR around DDB1 recruitment would be. Given that merely the addition of this pyridine is responsible for converting an inhibitor into a degrader, we hypothesized that—in common with IMiD-based glues—the SAR might be tight and intolerant of many changes. The X-ray crystal structure revealed that the pyridine ring sits in a narrow cleft between CDK12 and the DDB1 BPC domain. The cleft is flanked by the DDB1 Arg928 residue, which forms a stacking interaction with the pyridine ring, and by the CDK12 Ile733 residue (Figure 1). It was expected that groups extending from the para position would not be tolerated due to a clash with DDB1 residues Arg947 and Asn907. These residues form key interactions with CDK12, so disruption of this is likely to limit ternary complex formation.

**Table 1:**
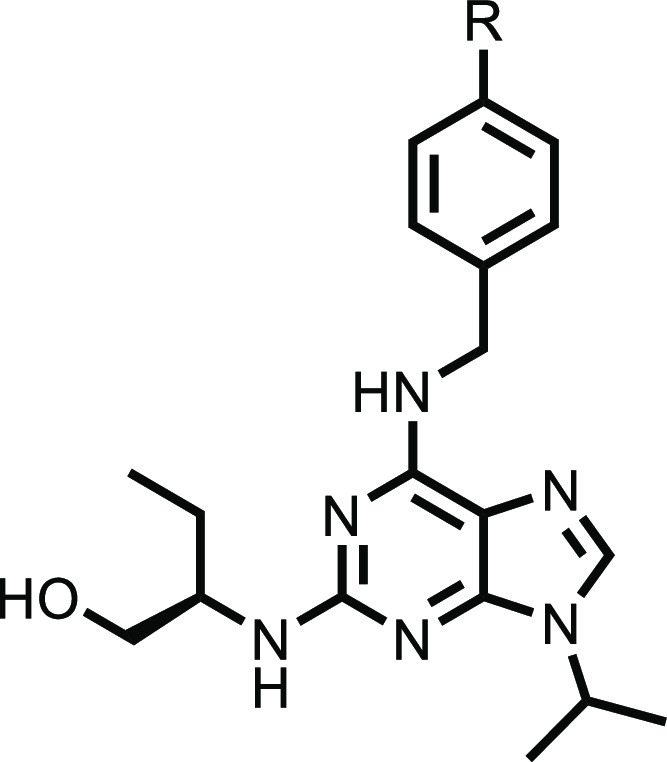

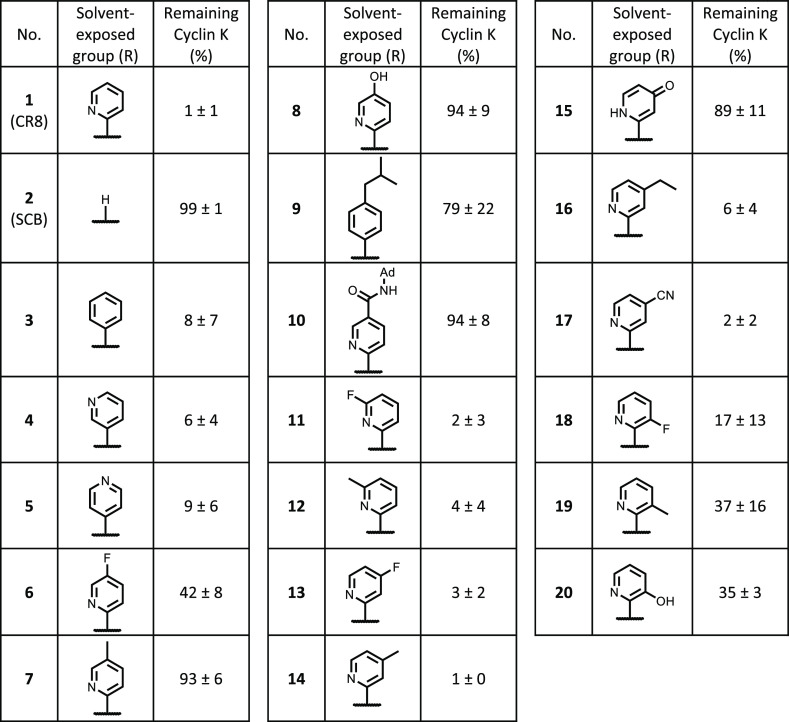
Normalized Levels of Cyclin K in HEK293T Cells after Treatment with the Compounds **1–20**^a^

a 1 μM for 2 h, quantified from Western blots. The results shown are the mean values ± SD from three independent experiments.

**Figure 2:**
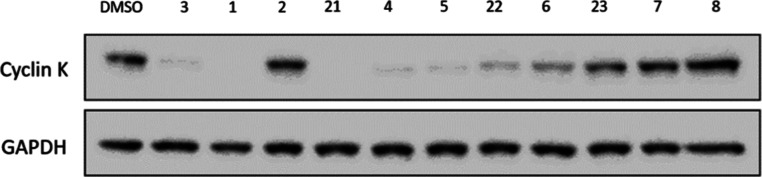
Representative Western blot showing levels of cyclin K in HEK293T cells following treatment with the indicated compounds at 1 μM for 2 h.

We therefore designed a range of compounds to explore the SAR of CR8 (**1**) by substituting the solvent-exposed pyridine, and then tested their ability to induce cyclin K degradation by Western blot (Table 1). To understand the importance of the pyridine nitrogen, phenyl (**3**), 3-pyridine (**4**), and 4-pyridine (**5**) analogues were prepared, which showed cyclin K degradation comparable to that of CR8 (**1**) (Figure 2, Table 1). Analogues featuring larger substituents such as fluorine (**6**), methyl (**7**), hydroxy (**8**), isobutyl (**9**), and adamantylamide (**10**) at the para position were expected to be inactive due to the limited space predicted by the crystal structure and were synthesized to test whether the model was predictive of activity. The smallest group, fluorine (**6**), already showed a reduction in degradation activity despite the small size differential to hydrogen, and increasing size to methyl (**7**) abrogated degradation further. This tight SAR is consistent with our initial expectations and consistent with the published model.

We postulated that groups that optimally fill the space between CDK12 and DDB1 would result in an improvement in ternary complex formation and thus improve the degradation ability. The crystal structure indicated space to incorporate additional substituents at either of the meta positions on the pyridine ring. Fluoro (**11**) and methyl (**12**) substituents at the 6-position of the 2-pyridyl ring were able to induce complete degradation of cyclin K. The same result was seen for the incorporation of 4-fluoro (**13**) and 4-methyl (**14**) substituents on the pyridine. Building on this, 4-ethyl (**16**) and 4-nitrile (**17**) analogues were synthesized and able to successfully induce degradation. The activity of the nitrile showed that some hydrophilicity is tolerated in this region; however, the pyrid-4-one (**15**) resulted in a total loss of degradation ability, which was unexpected as it is of similar size to the methyl analogue (**12**). The addition of a fluoro group (**18**) to the ortho position of the ring was expected to be tolerated based on its small size, and results confirmed that this analogue was able to induce degradation. However, moving to the slightly larger methyl group (**19**) or hydroxy group (**20**) was expected to cause a twist in the pyridine ring that could negatively impact the degradation ability due to the narrow shape of the cleft. Both analogues showed reduced degradation ability compared to CR8, indicating that although coplanarity of the rings is not essential for degradation, it is preferred.

**Figure 3:**
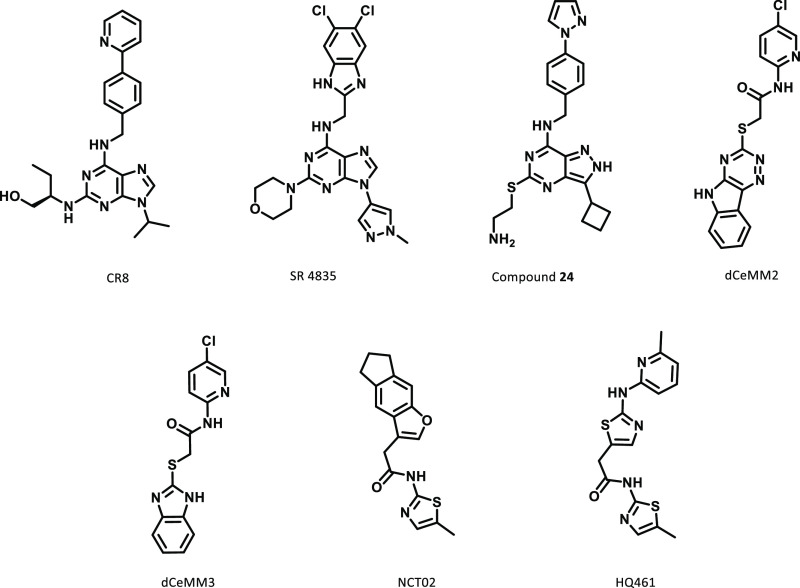
Structures of other published cyclin K degraders.

A further key objective was to explore whether more significant structural changes would be tolerated. There are a range of other cyclin K degraders that have been identified since the initial discovery of CR8, indicating that multiple scaffolds can be used to trigger degradation (Figure 3).^25−28^ Despite this, almost all of the degraders feature planar aromatic groups in the solvent-exposed region. Hence, it was predicted that introducing more 3-dimensionality to this area would reduce ternary complex formation due to the cleft being relatively narrow. Furthermore, the pi-stacking interaction between pyridine and DDB1 Arg928 has been deemed a key interaction, and losing this was predicted to negatively affect the degradation ability. A range of analogues was synthesized to test this hypothesis, with results showing that 3,6-dihydro-2*H*-pyran (**21**), which sits in a puckered conformation, was still capable of inducing complete degradation comparable to CR8, while the cyclohex-1-ene (**22**) analogue induced partial degradation (Table 2). This result demonstrates that, perhaps surprisingly, a broad range of solvent-exposed groups can be used to trigger cyclin K degradation, including nonaromatic rings. When cyclohex-1-ene was hydrogenated to cyclohexane (**23**), the degradation ability was significantly reduced. The reduction in degradation can be rationalized by comparing the conformations of the two rings, with cyclohexane favoring the more puckered chair conformation and consequently sitting further out of plane with the phenyl ring when compared to the half-chair conformation of the cyclohex-1-ene. The shift in conformation is subtle, showing that minor changes to the structure can have a large impact on degradation ability. An N-linked piperazine (**24**) analogue showed significantly reduced degradation ability, and the morpholine (**25**) analogue showed only partial degradation. Given that morpholine (**25**) and 3,6-dihydro-2*H*-pyran (**21**) are likely to adopt a similar conformation, it is possible that other factors affect the degradation ability in addition to the effect of planarity. One possibility was that the increased hydrophilicity of **25** reduced cell permeability, limiting degradation. However, PAMPA permeability data (Table 3) demonstrated that the two compounds have comparable permeabilities, suggesting that this is not a cause of the differences.

**Table 2:**
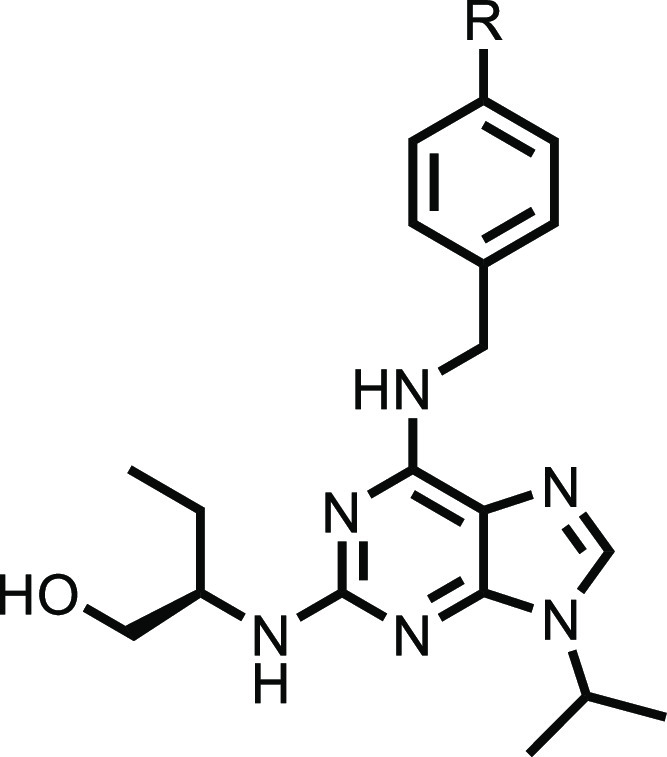

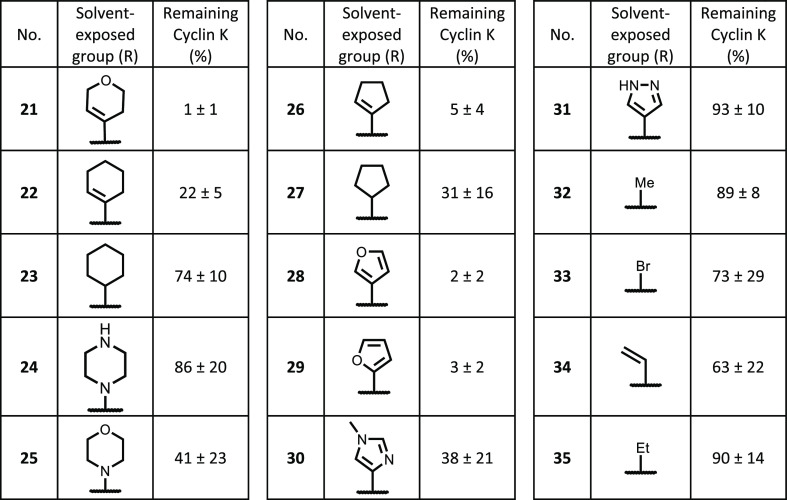
Normalized Levels of Cyclin K in HEK293T Cells after Treatment with Compounds **21–35**^a^

a 1 μM for 2 h, quantified from Western blots. The results shown are the mean values ± SD from three independent experiments.

**Table 3:**
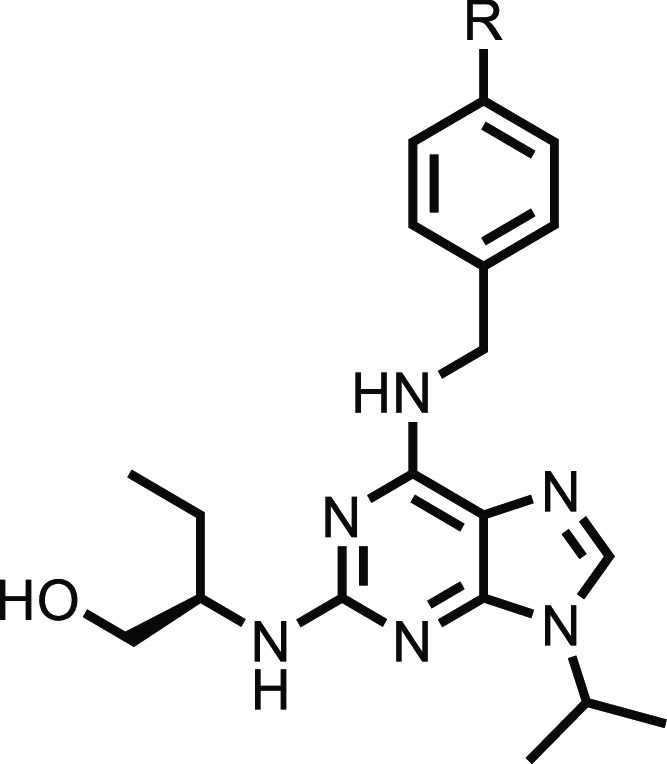

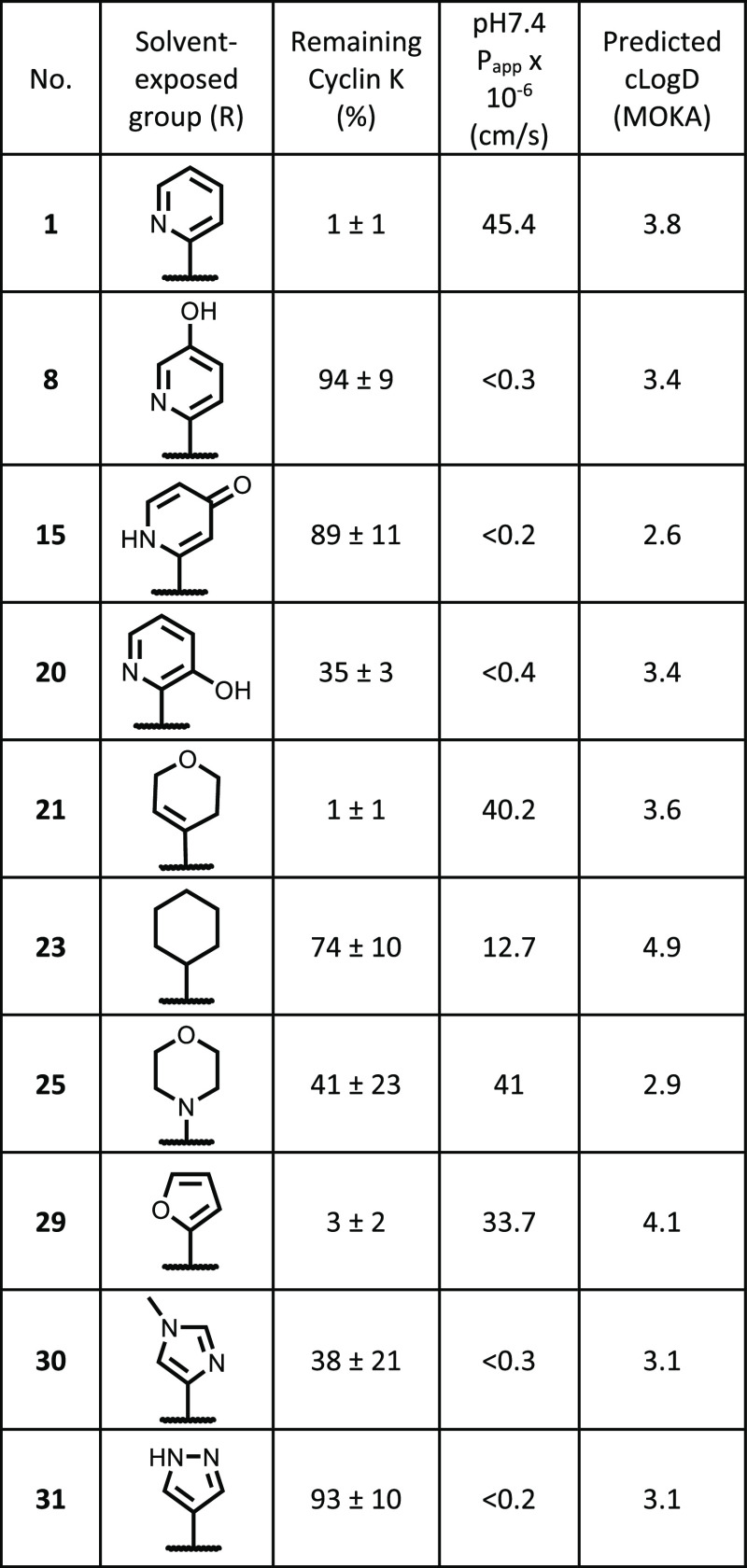
PAMPA Permeability Data and Predicted Log*D* for a Range of Compounds

Having determined that the replacement of pyridine with partially saturated rings was tolerated, we investigated whether smaller groups could be used to induce degradation by replacing the CR8 pyridine with a range of 5-membered rings. We hypothesized that these smaller groups may not properly fill the cleft between CDK12 and DDB1, reducing interactions and, therefore, may lead to reduced degradation ability. Based on the activity of the cyclohex-1-ene analogue (**22**), a corresponding cyclopent-1-ene (**26**) analogue was synthesized that showed complete cyclin K degradation (Table 2). Hydrogenation of the double bond provided a cyclopentane analogue (**27**) that demonstrated only partial degradation, mimicking the trend seen with hydrogenation from cyclohex-1-ene (**22**) to cyclohexane (**23**). Surprisingly, the furan analogues (**28** and **29**) were able to induce complete degradation, showing that this group is large enough to sufficiently stabilize the interaction with DDB1. This ability of such small substituents to stabilize the interaction between two large protein complexes could be due to the high level of pre-existing surface complementarity between CDK12 and DDB1. The more hydrophilic *N*-methyl imidazole (**30**) also resulted in a decreased degradation ability, and the pyrazole (**31**) analogue resulted in a complete loss of degradation; however, this is likely explained by the compound having low permeability (Table 3). Having established that a variety of 5 and 6 membered rings could be tolerated, we sought to investigate the minimum substituent required to induce cyclin K degradation. Analogues that replace pyridine with a methyl (**32**), bromo (**33**), ethene (**34**) or ethyl (**35**) group were synthesized and tested at 1 μM but were able to induce little to no degradation of cyclin K, pointing toward the requirement for a larger solvent-exposed group to fill the cleft.

**Figure 4:**
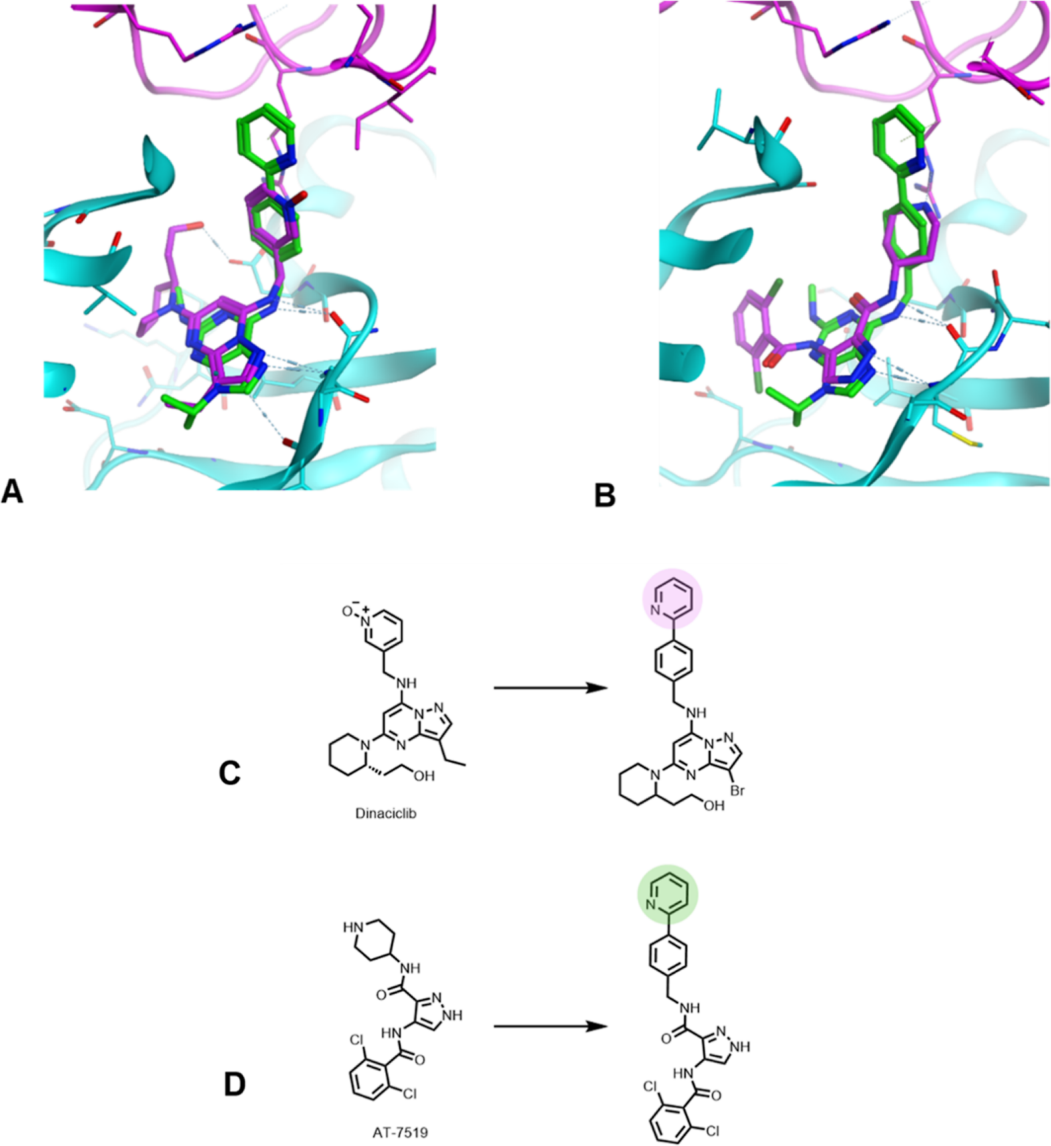
Converting other pan-CDK inhibitors into degraders. (A) Crystal structure of the DDB1–CR8–CDK12–cyclin K-complex with dinaciclib docked. (B) Crystal structure of the DDB1–CR8–CDK12–cyclin K-complex with AT-7519 docked, showing similar binding modes to CR8. Hydrogen bonds are shown in blue. (C) Structure of dinaciclib on the left and potential degrader shown on the right. (D) Structure of AT-7519 on the left and potential degrader. The solvent-exposed group is shown in pink/green.

**Figure 5:**
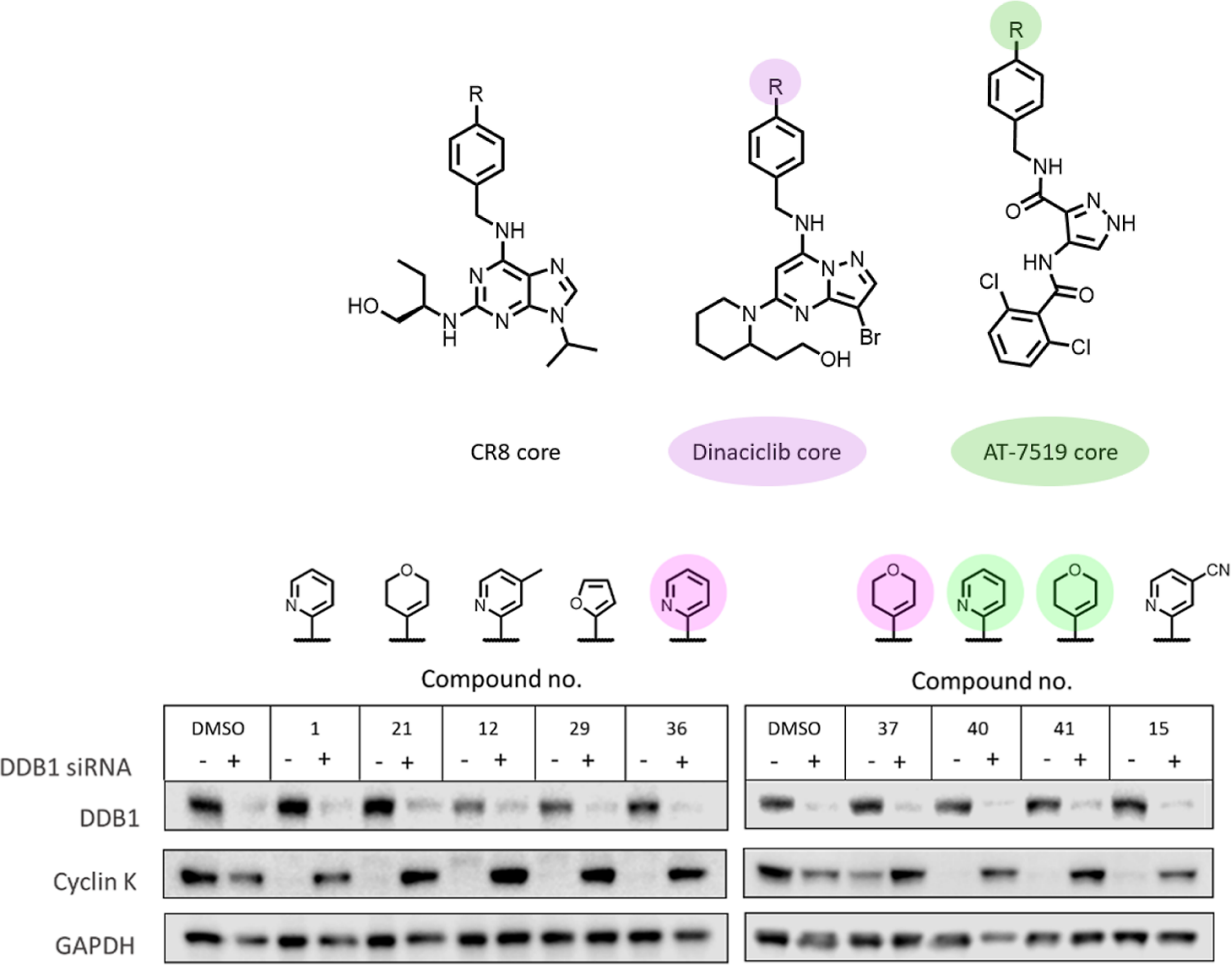
DDB1 knockdown using siRNA was performed for select compounds. Analogues of the dinaciclib core are highlighted in pink, and analogues of the AT-7519 core are highlighted in green. Unhighlighted compounds are based on the CR8 core.

We next aimed to take these key degradation features and expand the concept to identify a novel series of cyclin K degraders that would aid in the understanding of whether the SAR developed for CR8 would be transferrable to other scaffolds. We theorized that other pan-CDK inhibitors could be converted into degraders through the addition of appropriately placed solvent-exposed groups. A range of pan-CDK inhibitors was docked into the crystal structure of the CDK12/DDB1 complex and overlaid with CR8 (Figure 4a,b). The overlays were examined to see which compounds have a synthetically tractable vector from the core structure out toward the solvent, where substituents could be incorporated in order to enable ternary complex formation with DDB1. We first focused on the inhibitor dinaciclib due to its high structural similarity and synthetic route to CR8 analogues (Figure 4c).^29^ Furthermore, dinaciclib is a more potent CDK inhibitor compared to CR8, and we hypothesized that increased binding affinity would lead to increased degradation ability. Dinaciclib’s structure was altered slightly due to the availability of chemical intermediates, with the ethyl group on the core being replaced with a bromo group. This was not expected to affect the binding affinity of the compound, as the bromo analogue has previously been shown to be equipotent to dinaciclib.^30^ The pyridine *N*-oxide was also replaced with a phenyl ring, analogous to the CR8 core, as based on the docking and previous drug discovery efforts, it was deemed not to be important for potency.^31^ We did not expect these changes to significantly alter the binding mode of the compound, meaning that substituents incorporated at the 4-position of the phenyl ring should sit in a position similar to the solvent-exposed groups in CR8. We also selected the compound AT-7519 to see if a more structurally divergent core could be used. Through docking the compound, it appeared that replacing the piperidine with a benzyl group and introducing the solvent-exposed group at the 4 position would result in a similar placement of the solvent-exposed group to the pyridine in CR8 (Figure 4a–d).^32^ From here, several of the solvent-exposed groups that we used on the CR8 core to successfully degrade cyclin K were chosen to append to the modified cores. The dinaciclib core showed complete degradation with pyridine (**36**), 4-methylpyridine (**38**), and furan (**39**) (Table 4). Interestingly, with 3,6-dihydro-2*H*-pyran (**37**), only partial degradation was seen at this time point. This was surprising as this analogue on the CR8 core (**21**) shows complete degradation (full curves shown in Figure S3), and, given the high structural similarity between CR8 and dinaciclib, this analogue was expected to perform similarly. On the AT-7519 core, complete degradation was also seen for pyridine (**40**), 3,6-dihydro-2*H*-pyran (**41**) and furan (**42**), showing that diverse binder scaffolds can be used to trigger degradation when solvent-exposed groups are positioned correctly to mediate the interaction between CDK12 and DDB1. To confirm that the new analogues were operating via the same mechanism as CR8, DDB1 was knocked down using siRNA (Figure 5); all of the compounds tested were DDB1-dependent, as shown by the abrogation of cyclin K degradation under the DDB1 knockdown condition.

**Table 4:**
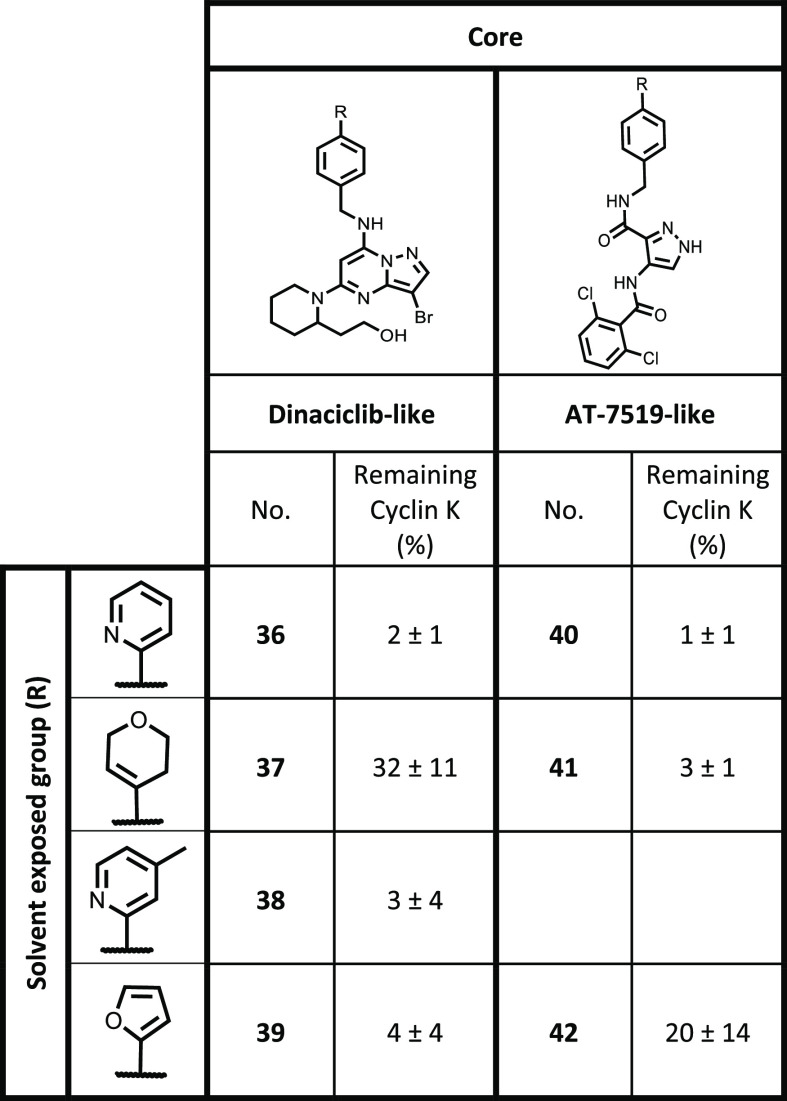
Normalized Levels of Cyclin K in HEK293T Cells after Treatment with Compounds **36–42** at 1 μM for 2 h Quantified from Western Blots^a^

a Results shown are the mean values ± SD from three independent experiments.

With the aim of comparing the degradation ability of the new analogues, we derived DC_50_ values by using Western blotting (Table 5). As CR8 works through binding to CDK12 (or CDK13) and recruiting DDB1, we would therefore expect that modifications that increase CDK12 affinity while not changing the portion of the molecule that binds DDB1 would lead to improved ternary complex formation and, hence, improved degradation. This approach has been previously used to improve the potency of BCL6 degraders.^33^ We therefore also measured CDK12 affinity using the KINOME*scan* binding assay (Table 5).^34^

**Table 5:**
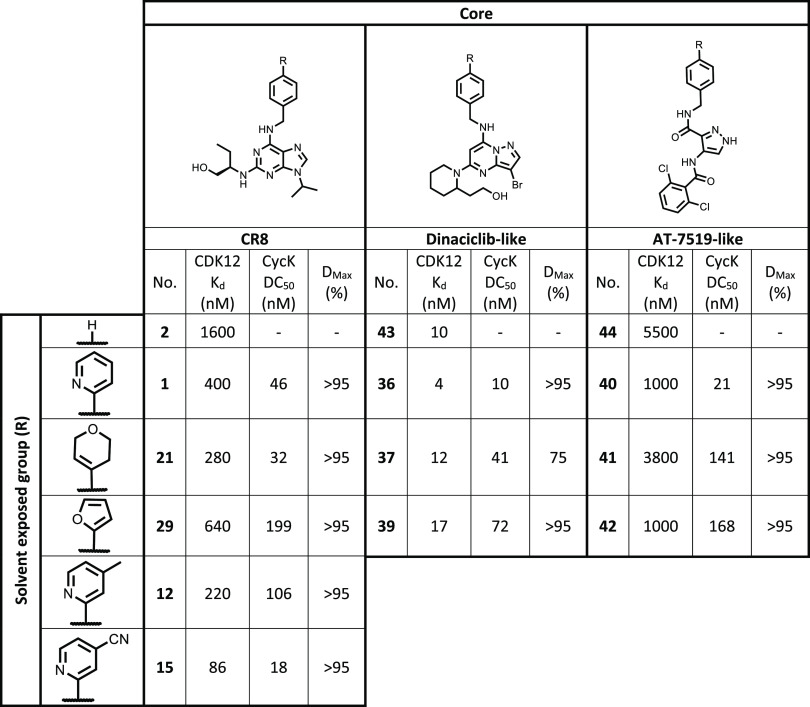
Table Showing DC_50_s Generated by Western Blot Quantification of Cyclin K Degradation, Representing 3 Biological Repeats, Compared to *K*_d_ Values for CDK12 Binding Generated Using the KINOME*scan* Assay (Eurofins DiscoverX)

As expected, modifications to the solvent-exposed group have a relatively small impact on the binding affinity but a more critical effect on degradation. For example, the furan analogues **29**, **39,** and **42** show similar CDK12 affinities to their pyridine counterparts **1** (CR8), **36,** and **40** but demonstrate a 4–8 fold decrease in degradation ability, indicating that this group may not optimally fill the space between CDK12 and DDB1. Nevertheless, it is interesting and perhaps surprising that, given the small portion of the ligand that contacts DDB1, a substantial amount of modification is tolerated, with multiple groups yielding potent degraders.

Larger differences in binding affinity were observed between series, with the dinaciclib analogues showing between 25 and 100-fold better affinity to CDK12 compared to the CR8/seliciclib analogues. Although this did lead to some improvement in cyclin K degradation activity, the magnitude of this difference was not consistent with the change in binding affinity, as evidenced by a comparison of compounds **1** and **36**, where a 100-fold increase in CDK12 affinity led to a smaller (fivefold) improvement in degradation as measured by DC_50_. In addition, despite showing a 25-fold increase in CDK12 binding affinity, the dinaciclib 3,6-dihydro-2*H*-pyran analogue (**37**) had comparable DC_50_ but a lower *D*_max_ than the CR8 equivalent (**21**). This highlights the importance of degradation kinetics in addition to binding affinity.

Although the AT-7519-based compounds were generally the least active degraders, the weaker CDK12 affinity of this series did not lead to a consistent drop in the degradation affinity. The pyridine-analogue **40** (the closest analogue to CR8) is a strong degrader with a similar DC_50_ to compound **36**, despite a 250-fold difference in CDK12 binding affinity. These findings suggest that it is possible, to some extent, to independently optimize inhibition and degradation activity for this class of molecular glue degraders. This could lead to compounds with different selectivity profiles and, therefore, potentially different biological activity—compound **36** in the dinaciclib series is both an effective CDK12 inhibitor and cyclin K degrader, whereas AT-7519-based degrader **40** is a similarly effective cyclin K degrader but with ∼500-fold weaker CDK12 affinity.

All parent compounds are reported to show kinase inhibition across multiple CDKs. Both seleciclib, the parent compound of CR8, and dinaciclib show sub-μM activity at CDK1, 2, 5, and 9.^24,29^ AT-7519 shows broader kinase activity, including against GSK3.^35^ Despite the affinity of CR8 for multiple members of the CDK family, only its binding to CDK12 or 13 leads to ternary complex formation with DDB1, and an analogous complex is not formed with CDK9.^23^ Consistent with this, cyclin K is the most degraded target, and CDK12 and CDK13 are also depleted after 5 h of treatment. Notably, though, over 50 other proteins were also depleted by at least twofold, including cyclin B1 and Aurora kinase.^23^ We therefore used proteomics to explore whether our modifications to the headgroup and core led to any differences in which proteins are depleted, and in particular, whether other CDKs, cyclins, or kinases were degraded.

**Figure 6:**
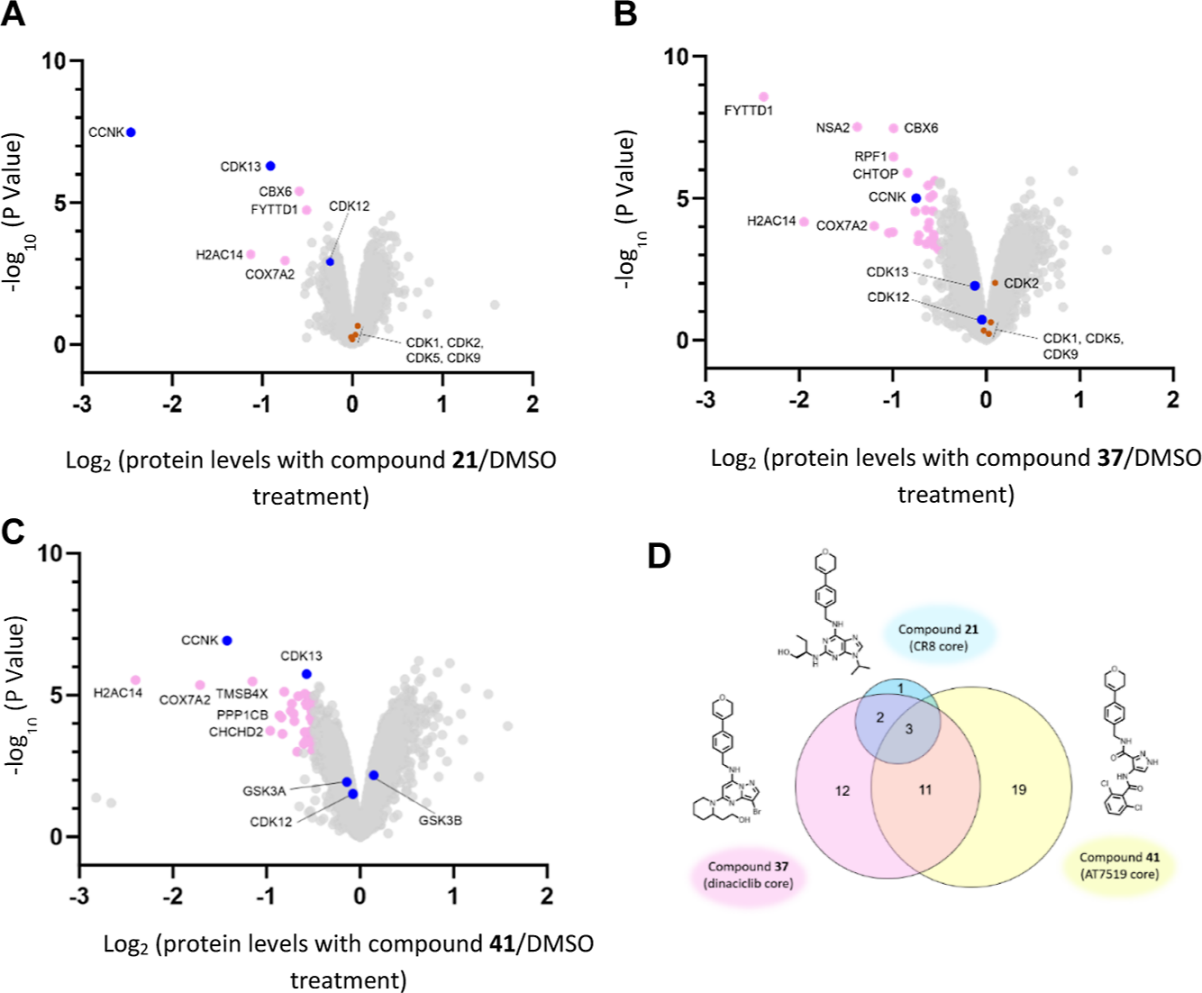
Whole-proteome quantification of HEK293T cells treated with 1 μM **21** (A), **37** (B), **41** (C), or DMSO for 2 h (*n* = 3). (D) Venn diagram showing the overlap in proteins that demonstrated at least a −0.5 log_2_ fold change with each of the three compounds tested.

Quantitative proteome-wide mass spectrometry was performed on cells treated with each of the three 3,6-dihydro-2*H*-pyran analogues (compounds **21**, **37,** and **41**) (Figure 6a–c). Despite the structural similarity between **21** and **37** and matching solvent-exposed groups across all three, substantial differences in the degradation profile were seen between compounds. The CR8 analogue (**21**) displayed the greatest specificity, with only six proteins showing at least a −0.5 log_2_ fold change after a 2 h treatment, compared to 28 proteins for the dinaciclib analogue (**37**) and 33 proteins for the AT-7519 analogue (**41**) (Figure 6d). Out of the three compounds tested, compound **21** showed the strongest effect on cyclin K. None of the three compounds showed any effect on CDK12, likely due to the short treatment time of 2 h, although CDK13 depletion is also observed, particularly with **21**. No other CDKs, cyclins, or kinases—including off-targets of parent compounds such as GSK3B—were significantly depleted following treatment with any of the three compounds, suggesting that groups mediating degradation are not generally transferable between related targets. Compound **37** (Figure 6b) shows a much less pronounced effect on cyclin K, with only a 0.75 log_2_ fold decrease compared to the 2.47 log_2_ fold decrease for compound **21**, reflecting the differences in the *D*_max_ between the two compounds (Table 5). Despite weaker cyclin K degradation, other proteins such as FYTTD1, H2AC14, and NSA2 are decreased to a much greater degree, suggesting that the effect on these proteins is unlikely to be a downstream consequence of cyclin K degradation. Compound **41** (Figure 6c) also showed pronounced effects on H2AC14 and NSA2, among other proteins. This offers a tantalizing hint that degraders for novel targets may be discoverable by scaffold-hopping from known degraders.

Through exploration of CR8 analogues, we have demonstrated that a range of solvent-exposed groups are able to trigger cyclin K degradation, showing that minimal additional interaction surface is required to stabilize the formation of the CDK12-DDB1 ternary complex. Our work demonstrates how X-ray crystal structures of ternary complexes can be used to predict and optimize the degradation ability of a known system, highlighting the importance of factors such as lipophilicity and the size of the solvent-exposed group. We have also shown that molecular glue degraders are optimizable through modifications to the core. Interestingly, while the increased CDK12 binding affinity of the dinaciclib pyridine analogue (**36**) relative to CR8 (**1**) does lead to some improvement in degradation, across our broader range of analogues, the binding vs degradation relationship is divergent. This suggests that degradation ability can be optimized to some extent independently from binding, not only through changes to the “solvent exposed” (putative DDB1 binding) region, which influence degradation without substantially changing binding affinity but also through modifications to the nonsolvent exposed target-binding region. Currently, it is not deemed possible to rationally design molecular glues for a specific target. However, this work suggests that it may be possible to discover new glues by exploring changes to solvent-exposed regions of existing target binders in order to stabilize an interaction with an E3 ligase component. While writing this manuscript, the Thomä group published work exploring the SAR of cyclin K degraders. Their work further supports the conclusion that a diverse range of solvent-exposed groups can be incorporated into cyclin K inhibitors and used to trigger cyclin K degradation.^36^ Our data expands on their findings—while they report that aromatic groups are required for degradation, we have identified nonaromatic substituents that enable potent degradation, as well as a subset of degraders showing improved activity compared to CR8. We demonstrate that degradation-inducing moieties can be transferred between structurally distinct series. The wide diversity of cyclin K degraders discovered in our combined studies indicates that the interaction interface does not have to be perfect to trigger target degradation, and this observation may facilitate the discovery of new glues. Recent works by Nomura et al. showed that appending a solvent-exposed covalent warhead to a range of target binders resulted in ligase recruitment and subsequent target degradation.^37^ This emphasizes the possibility of building on existing ligands to develop molecular glues. While this approach may necessitate some prerequisite surface complementarity between the target protein and the recruited E3 ligase, it still presents a promising outlook for the future of molecular glue design.

## Methods

### Cell Culture

HEK293T human epithelial kidney cells were cultured in DMEM (Gibco/Life Technologies), containing 25 mM d-glucose, 4 mM l-glutamine, and 1 mM sodium pyruvate, and supplemented with 10% (v/v) FBS and 1% nonessential amino acids (Gibco/Life Technologies). Cells were incubated at 37 °C in a humidified atmosphere with 5% CO_2_.

### Western Blotting

Cells were washed with PBS and lysed (20 mM HEPES pH 7.5, 10% glycerol, 0.4 M KCl, 0.4% Triton X-100, 15 mM EDTA, and 1× protease and phosphatase inhibitors). The insoluble fraction was removed by centrifugation. Protein quantification was performed using a Bradford assay and compared to a BSA calibration curve. Equal amounts of lysates were run on SDS–PAGE 4–12% Bis–Tris Protein Gels (NuPAGE, Thermo Fisher Scientific) and then transferred onto Immobilon-P PVDF Membrane (Millipore, IPVH00010) using a Mini Trans-Blot Cell (Bio-Rad, 1703930). The membranes were blocked with 5% bovine albumin serum (BSA) in Tris-buffered saline with Tween 20 (TBS-T) and incubated with primary antibodies overnight at 4 °C. The membranes were washed three times with TBS-T and incubated with HRP-conjugated secondary antibody (BioRad) for 2 h. Membranes were washed again three times with TBS-T before being developed using Pierce ECL Western Blotting Substrate (Thermo, 32106) and visualized using LI-COR. Antibodies were purchased from Cell Signaling Technology: anti-GAPDH (D4C6R) and anti-DDB1 (D4C8) or Bethyl Laboratories: anti-Cyclin K (A301-939A). The following HRP-linked secondary antibodies were used by dilution in 5% BSA/TBS-T: antirabbit IgG (BioRad, 5213–2504) and antimouse IgG (BioRad, 103005).

Unedited blots containing the marker lane are available on request. Western blots were quantified using ImageJ. Cyclin K levels were normalized using GAPDH as a loading control. DC_50_ values were calculated from concentration–response curves using nonlinear regression fit to a 4-parameter curve by GraphPad Prism.

### siRNA Knockdown

HEK293T was cultured in DMEM and seeded into 6-well plates. DBB1 siRNA (Horizon Discovery SMARTPool ON-TARGETplus siRNA) and control nucleic acid preparations were diluted to 5 nM with serum-free media (OptiMEM, Gibco) and transfection reagent (Lipofectamine RNAiMax, Thermo Fisher Scientific) and added to the appropriate well. Cells were incubated at 37 °C in a humidified atmosphere with 5% CO_2_ for 48 h before treatment with compounds.

### Molecular Docking

Virtual molecular docking of dinaciclib and AT-7519 was performed using *Molecular Operating Environment (MOE)* v2020.09 (Chemical Computing Group ULC, Montreal, Canada) with the crystal structure of the CDK12-Cyclin K- DDB1 complex (PDB: 6TD3) with CR8 bound as an input.

### Proteome-Wide Quantitation by Mass Spectrometry

HEK293T were cultured in DMEM and seeded into 6-well plates. Cells were treated with a 1 μM solution of test compound or DMSO control for 2 h. Cells were collected and washed with PBS, snap-frozen, and stored at −80 °C.

Samples for quantitative proteomics were prepared using the SimPLIT workflow.^38^ Offline peptide fractionation was based on high-pH reverse phase (RP) chromatography using the Waters XBridge C18 column (2.1 × 150 mm, 3.5 μm) on a Dionex Ultimate 3000 HPLC system at a 0.85% gradient with a flow rate of 0.2 mL/min. Mobile phase A was 0.1% ammonium hydroxide, and mobile phase B was 100% acetonitrile and 0.1% ammonium hydroxide. Retention time-based fractions (every 30 s) are collected and pooled into 24 samples for LC–MS analysis.

LC–MS analysis was performed on a Dionex UltiMate 3000 UHPLC system coupled with an Orbitrap Ascend Tribrid mass spectrometer (Thermo Scientific). Samples were analyzed with the PepMap C18 capillary column (75 μm × 50 cm, 2 μm) at 50 °C. Mobile phase A was 0.1% formic acid, and mobile phase B was 80% acetonitrile and 0.1% formic acid. The gradient separation method was as follows: 150 min gradient up to 38% B, for 10 min up to 95% B, for 5 min isocratic at 95% B, re-equilibration to 5% B in 10 min, and for 10 min isocratic at 5% B. Mass spectrometry data were acquired using a TMT-SPS-MS3 with online real-time database search (RTS). Precursors between 400 and 1600 *m*/*z* were selected with a mass resolution of 120,000, standard automatic gain control (AGC) of 2 × 10^5^, and IT (injection time) of 50 ms. With the top speed mode in 3 s. Ion trap MS2 scans (HCD fragmentation, 32% collision energy, AGC at 1 × 10^5^, max IT at 35, turbo scan rate). RTS was enabled, and quantitative TMT-SPS-MS3 scans (resolution of 45,000, AGC at 1 × 10^5^, max IT at 200 ms) were collected.

The SEQUEST-HT search engine was used to analyze the acquired mass spectra in Proteome Discoverer 3.0 (Thermo Scientific) for protein identification and quantification. The precursor mass tolerance was set at 20 ppm, and the fragment ion mass tolerance was set at 0.5 Da. Spectra were searched for fully tryptic peptides with a maximum of 2 mis-cleavages. TMTpro on lysine residues and peptide N termini (304.2071 Da) and carbamidomethylation of cysteine residues (+57.0215 Da) were set as static modifications, while oxidation of methionine residues (+15.9949 Da) and deamidation of asparagine and glutamine (+0.9848 Da) were set as variable modifications. Peptide confidence was estimated with the Percolator. The peptide FDR was set at 0.01, and validation was based on the q value and a decoy database search. All spectra were searched against UniProt-SwissProt proteome *Homo sapiens* protein entries (version 14 Jan 2023) appended with contaminants and media proteins. The reporter ion quantifier node included a TMTpro quantification method with an integration window tolerance of 15 ppm and an integration method based on the most confident centroid peak at the MS3 level. Only unique peptides were used for quantification, with protein groups considered for peptide uniqueness. Peptides with an average reporter signal-to-noise ratio of >3 were used for protein quantification. Correction for the isotopic impurity of reporter quantification values is applied. Only spectra with at least 50% of the SPS masses matching the identified peptides are used for quantification. Normalized protein abundance values of compound treatments were compared to DMSO control using a moderated *t*-test in Limma as implemented in Phantasus. Differentially regulated proteins relative to the DMSO control (log 2FC = 0.5 and *p*-value < 0.001) were reported.

## Supporting Information Available

Synthesis and characterization of compounds, representative Western blots and examples of concentration–response curves and pDC_50_ values with associated errors, and data from proteome-wide tandem mass tag spectrometry for compounds **21**, **37,** and **41** (PDF)
(XLSX)
