# Supplementary material for: Degradation by Design: New Cyclin K Degraders from Old CDK Inhibitors
Source: ACS Chem Biol. 2024 Jan 9;19(1):173–84. doi: 10.1021/acschembio.3c00616 (PMC10804372; doi:10.1021/acschembio.3c00616)
Supplement: Supplementary file 1 — cb3c00616_si_001.pdf [file cb3c00616_si_001.pdf]

Supporting Information

Degradation by design: new cyclin K degraders from old CDK inhibitors

Katie L. Thomas[†], Habib Bouguenina[†], Daniel S. J. Miller[†], Fernando J. Sialana[‡], Thomas G. Hayhow[§], Jyoti S. Choudhary[‡], Olivia W. Rossanese[†], Benjamin R. Bellenie[†,*].

[†]Centre for Cancer Drug Discovery, The Institute of Cancer Research, London SM2 5NG, U.K; [‡] Functional Proteomics Group, The Institute of Cancer Research, London SW3 6JB, U.K; [§]Oncology R&D, AstraZeneca, 1 Francis Crick Avenue, Cambridge Biomedical Campus, Cambridge CB2 0AA, U.K.
* Corresponding author. Email: Benjamin.Bellenie@icr.ac.uk

Table of Contents

S1. Experimental Procedures **S2**
S2. ^{1}HNMR and ^{13}CNMR spectra of key compounds **S38**
S3. Western Blotting Data and concentration response **S49**
S4. pDC$_{50}$ values **S52**

S1. Experimental Procedures

General Procedure 1 – S_NAR 1

To a solution of an amine (1 eq.) and DIPEA (2 eq.) in 1-butanol (0.25 M) was added an aryl or heteroaryl chloride (1 eq.). The reaction was heated to 110°C for 2 – 5 hours. The solution was cooled to rt and filtered, washing with 1-butanol.

General Procedure 2 – N-alkylation

To a solution of an amine (1 eq.) and K_2CO_3 (3 eq.) in DMSO (0.5 M) was added an alkyl bromide (3 eq.) and the solution was stirred at rt for 2- 4 hours. The reaction was diluted into EtOAc and water. The aqueous layer was extracted with EtOAc and the combined organics were washed with brine, dried over $MgSO_4$ and concentrated *in vacuo*.

General Procedure 3 – S_NAR 2

A chloride (1 eq.) and an amine (8 eq.) were combined in a vial. The vial was sealed and heated to 160°C for 16 hours. Water was added and was extracted with EtOAc. The organic layers were combined, washed with brine, dried over $MgSO_4$. The solution was concentrated *in vacuo*.

General Procedure 4 – Suzuki reaction

An aryl bromide (1 eq.), boronic acid/ester (1.2 eq.) and potassium phosphate (3 eq.) were dissolved in 1,4 dioxane and water (9:1 ratio, 0.1 M). The solution was degassed (N_2) and Pd(dppf)Cl_2.DCM (0.05 eq.) was added. The reaction was heated to 100°C under microwave irradiation for 2 – 4 hours. The solution was cooled to rt, filtered through celite and concentrated *in vacuo*.

General Procedure 5 – S_NAR 3

To a stirred solution of an aromatic chloride (1 eq.) and DIPEA (4 eq.) in 1,4-dioxane (0.1 M) was added an amine (1 eq.) and the solution stirred at rt for 2 – 18 hours. The solution was concentrated *in vacuo* and the residue purified by column chromatography.

General Procedure 6 – Amide formation

To a stirred solution of a carboxylic acid (1 eq.), DMF (0.1 eq.) in DCM (0.1 M) was added oxalyl chloride (1.5 eq.). The solution was stirred at rt for 18 hours. The reaction was concentrated *in vacuo* and washed with EtOAc. The intermediate was dissolved in 1,4-dioxane (0.1 M) and triethylamine (3 eq.) and an amine (1.1 eq.) were added. The reaction was stirred at rt for 18 hours followed by concentrated *in vacuo*.

General Procedure 7 – Boc Deprotection

To a stirred solution of a boc protected amine (1 eq.) in DCM (0.1 M) was added 4M HCl in 1,4-dioxane (10 eq.) and the reaction stirred at rt for 1 – 4 hours. The solution was concentrated *in vacuo*.

General Procedure 8 – S$_N$AR 4

To a stirred solution of an aromatic chloride (1 eq.) and $NaHCO_3$ (2 eq.) in NMP (0.15 M) was added an amine (2 eq.) and the solution stirred at 160°C overnight. The solution was concentrated *in vacuo* followed by dilution into EtOAc. Water was added and was extracted with EtOAc. The organic layer was washed with brine, dried over $MgSO_4$ and solvent was removed *in vacuo*.

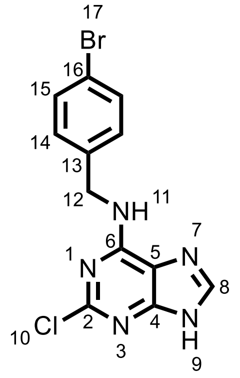

N-[(4-bromophenyl)methyl]-2-chloro-9H-purin-6-amine (Intermediate A)

The product was synthesised following general procedure **1**, using 2,6-dichloro-9H-purine (500 mg, 2.65 mmol) and 4-bromobenzylamine (0.33 mL, 2.65 mmol). The product was dried *in vacuo* to afford the title compound (852 mg, 95%) as a white solid. LC-MS purity >95%, ret. time 1.34 mins. HRMS (ESI +ve): $C_{12}H_9BrClN_5$ $[M+H]^+$: 337.9801 (Found: 337.9801). ^{1}H NMR (600 MHz, DMSO-d$_6$) δ 13.08 (s, 1H, H9), 8.73 (s, 1H, H11), 8.15 (s, 1H, H8), 7.52 (d, *J* = 8.0 Hz, 2H, H15), 7.31 (d, *J* = 8.3 Hz, 2H, H14), 4.63 – 4.59 (m, 2H, H12). ^{13}C NMR (151 MHz, DMSO-d$_6$) δ 155.21 (C6), 153.32 (C2), 151.10 (C4), 140.13 (C8), 139.36 (C16), 131.64 (C14), 130.04 (C15), 120.31 (C13), 118.36 (C5), 43.02 (C12).

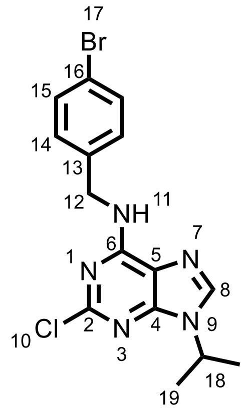

N-(4-bromobenzyl)-2-chloro-9-isopropyl-*9H*-purin-6-amine (Intermediate B)

The product was synthesised following general procedure **2**, using N-[(4-bromophenyl)methyl]-2-chloro-9*H*-purin-6-amine (**Intermediate A**, 852 mg, 2.51 mmol) and 2-bromopropane (0.71 mL, 7.55 mmol). The reaction was stirred at rt overnight. Potassium carbonate (347 mg, 2.51 mmol) and 2-bromopropane (0.24 mL, 2.51 mmol) were added and the reaction was stirred at rt for 2 hours followed by heating to 40°C for 2 hours. Water was added and was extracted with EtOAc. The organic layers were combined, washed with brine, dried over $MgSO_4$ and solvent was removed *in vacuo*. The residue was purified by column chromatography (0 → 60% EtOAc in cyclohexane) to

afford the title compound (648 mg, 68%) as a white solid. HRMS (ESI +ve): $C_{15}H_{16}BrClN_5$ $[M+H]^+$: 380.0277 (Found: 380.0276). ^{1}H NMR (600 MHz, DMSO-d_6) δ 8.86 – 8.81 (m, 1H, H12), 8.30 (s, 1H, H8), 7.51 (d, *J* = 8.4 Hz, 2H, H15), 7.30 (d, *J* = 8.1 Hz, 2H, H14), 4.67 (h, *J* = 6.8 Hz, 1H, H18), 4.60 (d, *J* = 6.2 Hz, 2H, H12), 1.50 (d, *J* = 6.8 Hz, 6H, H19). ^{13}C NMR (151 MHz, DMSO-d_6) δ 155.29 (C6), 153.14 (C2), 149.90 (C4), 140.02 (C8), 139.28 (C13), 131.62 (C15), 130.01 (C14), 120.30 (c16), 118.92 (C5), 47.22 (C18), 43.02 (C12), 22.58 (C19).

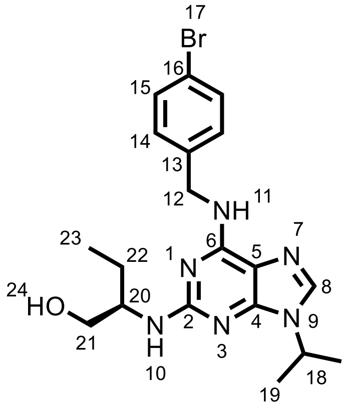

(R)-2-((6-((4-bromobenzyl)amino)-9-isopropyl-*9H*-purin-2-yl)amino)butan-1-ol (Intermediate C)

The product was synthesised following general procedure **3**, using N-(4-bromobenzyl)-2-chloro-9-isopropyl-*9H*-purin-6-amine (**intermediate B,** 540 mg, 1.42 mmol) and (R)-2-amino-1-butanol (1.1 mL, 11.35 mmol). The residue was purified by column chromatography (60 → 100% EtOAc in cyclohexane) to afford the title compound (587 mg, 95%) as an orange oil that solidified upon standing. HRMS (ESI +ve): $C_{19}H_{26}BrN_6O$ $[M+H]^+$: 433.151 (Found: 433.1348). ^{1}H NMR (600 MHz, $CDCl_3$) δ 7.51 (s, 1H, H8), 7.46 (d, *J* = 8.4 Hz, 2H, H15), 7.26 (d, *J* = 8.3 Hz, 2H, H14), 6.02 (s, 1H, H24), 4.88 (d, *J* = 6.1 Hz, 1H, H10), 4.81 – 4.67 (m, 2H, H12), 4.66 – 4.58 (m, 1H, H18), 3.92 – 3.87 (m, 1H, H20), 3.84 (dd, *J* = 10.8, 2.6 Hz, 1H, H21), 3.65 (dd, *J* = 10.7, 7.8 Hz, 1H, H21), 1.69 – 1.57 (m, 2H, H22), 1.55 (d, *J* = 6.8, 1.2 Hz, 6H, H19), 1.04 (t, *J* = 7.4 Hz, 3H, H23). ^{13}C NMR (151 MHz, CDCl3) δ 160.03 (C2), 154.77 (C6), 150.03 (C4), 137.95 (C16), 134.73 (C8), 131.65 (C15), 129.35 (C14), 121.14 (C13), 114.66 (C5), 68.53 (C21), 56.37 (C20), 46.48 (C18), 43.53 (C12), 25.02 (C22), 22.60 (C19), 10.94 (C23).

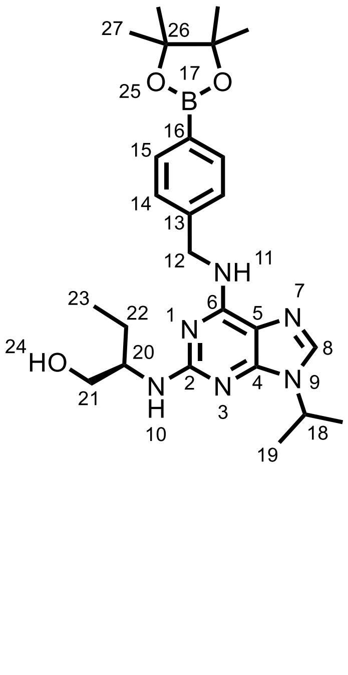

(R)-2-((9-isopropyl-6-((4-(4,4,5,5-tetramethyl-1,3,2-dioxaborolan-2-yl)benzyl)amino)-9H-purin-2-yl)amino)butan-1-ol (Intermediate D)

(R)-2-((6-((4-bromobenzyl)amino)-9-isopropyl-*9H*-purin-2-yl)amino)butan-1-ol (**intermediate C,** 200 mg, 0.46 mmol) , Bis(pinacolato)diboron (140 mg, 0.55 mmol) and potassium acetate (90 mg, 0.92 mmol) were dissolved in dry 1,4-Dioxane (3 mL, 0.15 M). The solution was degassed (N_2) and Pd(dppf)Cl_2.DCM (18 mg, 0.01 mmol) was added and the reaction heated to 100°C overnight. The reaction was filtered through a pad of celite and concentrated *in vacuo*. The residue was purified by column chromatography (0 →15% MeOH in DCM) and concentrated *in vacuo* to afford the title compound (188 mg, 85%) as an orange oil. ^{1}H NMR (600 MHz, $CDCl_3$) δ 7.79 (d, *J* = 8.0 Hz, 2H, H15), 7.53 (s, 1H, H8), 7.39 (d, *J* = 7.8 Hz, 2H, H14), 4.87 (d, *J* = 6.0 Hz, 1H, H10), 4.81 (s, 2H, H12), 4.63 (p, *J* = 6.8 Hz, 1H, H18), 3.92 – 3.86 (m, 1H, H20), 3.84 (dd, *J* = 10.7, 2.5 Hz, 1H, H21), 3.64 (dd, *J* = 10.7, 7.8 Hz, 1H, H21), 1.67 – 1.57 (m, 2H, H22), 1.56 (d, *J* = 5.7 Hz, 6H, H19), 1.36 (s, 12H, H27), 1.04 (t, *J* = 7.4 Hz, 3H, H23). ^{13}C NMR (151 MHz, $CDCl_3$) δ 160.56 (C2), 154.88 (C6), 150.28 (C4), 141.91 (C16), 135.11 (C13), 134.59 (C8), 126.95 (C14, C15), 114.67 (C5), 83.78 (C26), 68.68 (C21), 56.42 (C20), 46.45 (C18), 44.46 (C12), 24.87 (C22), 24.60 (C27), 22.61 (C19), 22.55 (C19), 10.95 (C23).

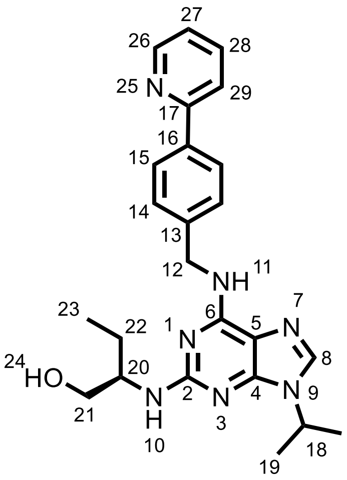

(R)-2-((9-isopropyl-6-((4-(pyridin-2-yl)benzyl)amino)-*9H*-purin-2-yl)amino)butan-1-ol (1)

(R)-2-((9-isopropyl-6-((4-(4,4,5,5-tetramethyl-1,3,2-dioxaborolan-2-yl)benzyl)amino)-*9H*-purin-2-yl)amino)butan-1-ol (**intermediate D,** 35 mg, 0.07 mmol) was coupled with 2-bromopyridine (0.01 mL, 0.11 mmol) according to general procedure **4**. The residue was purified by reverse phase column (10 → 60% MeOH in water (0.1% formic acid)) and passed through an SCX cartridge (washing with MeOH and eluting with 2M NH_3/MeOH). concentration *in vacuo* afforded the title compound (5 mg, 15%) as a white solid. LCMS purity >95%, ret. time 1.03 mins. HRMS (ESI +ve): $C_{24}H_{30}N_7O$ [M+H]$^+$: 432.2512 (Found: 432.2511). ^{1}H NMR (600 MHz, $CDCl_3$) δ 8.70 (d, *J* = 4.5 Hz, 1H, H26), 7.97 (d, *J* = 9.0 Hz, 2H, H15), 7.78 – 7.71 (m, 2H, H27, H29), 7.53 (s, 1H, H8), 7.49 (d, *J* = 8.2 Hz, 2H, H14), 7.26 – 7.22 (m, 1H, H28), 6.14 (s, 1H, H24), 4.97 – 4.92 (m, 1H, H10), 4.91 – 4.77 (m, 2H, H12), 4.63 (hept, *J* = 6.8 Hz, 1H, H18), 3.95 – 3.87 (m, 1H, H20), 3.84 (dd, *J* = 10.7, 2.6 Hz, 1H, H21), 3.65 (dd, *J* = 10.7, 7.8 Hz, 1H, H21), 1.69 – 1.58 (m, 2H, H22), 1.56 (d, *J* = 6.8 Hz, 6H, H19), 1.04 (t, *J* = 7.5 Hz, 3H, H23). ^{13}C NMR (151 MHz, $CDCl_3$) δ 157.13 (C17, C2), 154.83 (C6), 149.68 (C26, C4), 139.65 (C16), 138.51 (C13), 136.75 (C29), 134.65 (C8), 128.06 (C14), 127.14 (C15), 122.09 (C28), 120.48 (C27), 114.72 (C5), 68.51 (C21), 56.38 (C20), 46.48 (C18), 44.25 (C12), 25.02 (C22), 22.61 (C19), 22.55 (C19), 10.94 (C23).

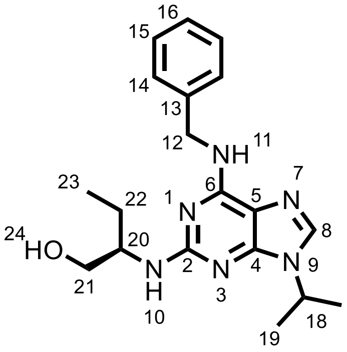

(R)-2-((6-(benzylamino)-9-isopropyl-*9H*-purin-2-yl)amino)butan-1-ol (2)

(R)-2-((6-((4-bromobenzyl)amino)-9-isopropyl-*9H*-purin-2-yl)amino)butan-1-ol (350 mg, 0.80 mmol) , Bis(pinacolato)diboron (246 mg, 0.96 mmol) and potassium acetate (317 mg, 3.23 mmol) were dissolved in 1,4-Dioxane (6 mL, 0.15 M). The solution was degassed (N_2) and Pd(dppf)Cl_2.DCM (33 mg, 0.04 mmol) was added and the reaction heated to 100°C overnight. Dehalogenation of the starting material led to formation of the title compound. The residue was purified by reverse phase column (20 → 65% MeOH in water (0.1% formic acid)) and passed through an SCX cartridge (washing with MeOH and eluting with 2M NH_3/MeOH). Concentration *in vacuo* afforded the title compound (55 mg, 20%) as an off-white solid. HRMS (ESI +ve): $C_{19}H_{30}N_6O$ $[M+H]^+$: 355.2246 (Found: 355.2243). ^{1}H NMR (600 MHz, $CDCl_3$) δ 7.51 (s, 1H, H8), 7.39 (d, *J* = 7.1 Hz, 2H, H14), 7.35 (t, *J* = 7.6 Hz, 2H, H15), 7.31 – 7.29 (m, 1H, H16), 5.98 (s, 1H, H24), 4.89 (d, *J* = 6.0 Hz, 1H, H10), 4.79 (s, 2H, H12), 4.62 (h, *J* = 6.8 Hz, 1H, H18), 3.94 – 3.88 (m, 1H, H20), 3.84 (dd, *J* = 10.7, 2.5 Hz, 1H, 21), 3.65 (dd, *J* = 10.7, 7.8 Hz, 1H, H21), 1.69 – 1.57 (m, 2H, H22), 1.55 (d, *J* = 6.8, 1.1 Hz, 6H, H19), 1.05 (t, *J* = 7.5 Hz, 3H, H23). ^{13}C NMR (151 MHz, $CDCl_3$) δ 160.09 (C2), 154.87 (C6), 150.20 (C4), 138.73 (C13), 134.60 (C8), 128.62 (C15), 127.75 (C14), 127.38 (C16), 114.75 (C5), 68.68 (C21), 56.41 (C20), 46.44 (18), 44.42 (C12), 25.04 (C22), 22.62 (C19), 22.55 (C19), 10.95 (C23).

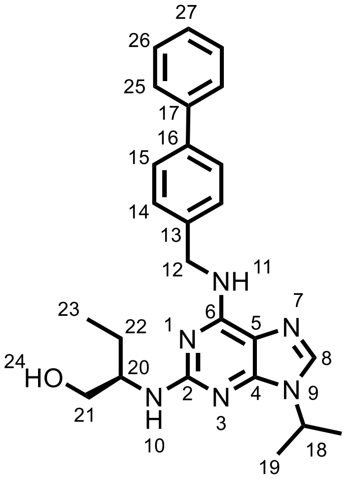

(R)-2-((6-(([1,1'-biphenyl]-4-ylmethyl)amino)-9-isopropyl-9H-purin-2-yl)amino)butan-1-ol (3)

(R)-2-((6-((4-bromobenzyl)amino)-9-isopropyl-*9H*-purin-2-yl)amino)butan-1-ol (50 mg, 0.12 mmol), was coupled with phenylboronic acid (17 mg, 0.14 mmol) according to general procedure **4**. The product was purified by reverse phase chromatography (30 → 60% MeOH in water (0.1% formic acid)) and SCX column (washing with MeOH and eluting with 2M NH_3/MeOH). Concentration *in*

vacuo afforded the title compound (7 mg, 12%) as a white solid. LCMS purity >95%, ret. time 1.32 mins. HRMS (ESI +ve): $C_{25}H_{31}N_6O$ $[M+H]^+$: 431.2559 (Found: 431.2553). ^1H NMR (600 MHz, $CDCl_3$) δ 7.61 – 7.57 (m, 4H, H14, H15), 7.53 (s, 1H, H8), 7.47 – 7.43 (m, 4H, H25, H26), 7.39 – 7.33 (m, 1H, H27), 6.13 (s, 1H, H24), 4.94 (d, *J* = 6.1 Hz, 1H, H11), 4.90 – 4.73 (m, 2H, H12), 4.63 (hept, *J* = 6.8 Hz, 1H, H18), 3.96 – 3.90 (m, 1H, H20), 3.85 (dd, *J* = 10.7, 2.6 Hz, 1H, H21), 3.66 (dd, *J* = 10.7, 7.8 Hz, 1H, H21), 1.69 – 1.58 (m, 2H, H22), 1.56 (d, *J* = 6.8 Hz, 6H, H18), 1.05 (t, *J* = 7.5 Hz, 3H, H23). 13C NMR (151 MHz, $CDCl_3$) δ 159.98 (C2), 154.85 (C6), 150.03 (C4), 140.85 (C17), 140.34 (C16), 137.84 (C13), 134.62 (C8), 128.77 (C14), 128.16 (C15), 127.35 (C25), 127.27 (C27), 127.09 (C26), 114.71 (C5), 68.61 (C21), 56.41 (C20), 46.48 (C18), 44.00 (C12), 25.03 (C22), 22.61 (C19), 22.55 (C19), 10.95 (C23).

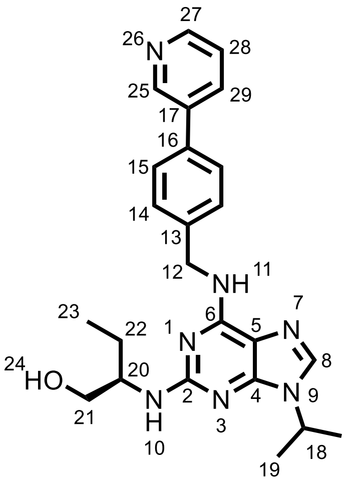

(R)-2-((9-isopropyl-6-((4-(pyridin-3-yl)benzyl)amino)-*9H*-purin-2-yl)amino)butan-1-ol (4)

((R)-2-((6-((4-bromobenzyl)amino)-9-isopropyl-*9H*-purin-2-yl)amino)butan-1-ol (30 mg, 0.070 mmol) was coupled with 3-pyridine boronic acid (10 mg, 0.083 mmol) according to general procedure **4**. The residue was purified by reverse phase column (30 → 50% MeOH in water (0.1% formic acid)) and concentrated *in vacuo* to afford the title compound (11 mg, 35%) as a white solid. LCMS purity >95%, ret. time 0.95 mins. HRMS (ESI +ve): $C_{24}H_{30}N_7O$ $[M+H]^+$: 432.2512 (Found: 432.2550). ^1H NMR (600 MHz, $CDCl_3$) δ 8.86 (s, 1H, H25), 8.61 (dd, *J* = 4.8, 1.6 Hz, 1H, H27), 7.88 (dt, *J* = 7.9, 2.0 Hz, 1H, H29), 7.59 – 7.54 (m, 3H, H8, H15), 7.51 (d, *J* = 8.0 Hz, 2H, H14), 7.38 (dd, *J* = 7.9, 4.8 Hz, 1H, H28), 5.98 (s, 1H, H24), 4.92 – 4.78 (m, 3H, H10, H12), 4.63 (h, *J* = 6.8 Hz, 1H, H18), 3.95 – 3.90 (m, 1H, H20), 3.85 (dd, *J* = 10.7, 2.5 Hz, 1H, H21), 3.66 (dd, *J* = 10.7, 7.8 Hz, 1H, H21), 1.70 – 1.58 (m, 2H, H22), 1.57 (dd, *J* = 6.7, 1.1 Hz, 6H, H19), 1.05 (t, *J* = 7.5 Hz, 3H, H23). ^{13}C NMR (151 MHz, $CDCl_3$) δ 160.02 (C2), 154.93 (C6), 149.85 (C4), 148.51 (C28), 148.30 (C30), 136.94 (C17), 136.33 (C16) 134.72 (C8), 134.27 (C29), 128.44 (C14), 127.37 (C15), 123.56 (C28), 114.13 (C5), 68.66 (C21), 56.42 (C20), 46.50 (C18), 44.03 (C12), 25.04 (C22), 22.62 (C19), 22.55 (C19), 10.95 (C23).

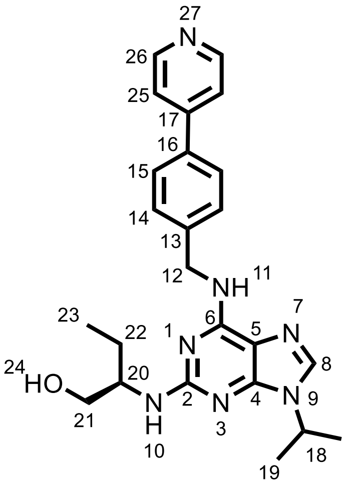

(R)-2-((9-isopropyl-6-((4-(pyridin-4-yl)benzyl)amino)-*9H*-purin-2-yl)amino)butan-1-ol (5)

(R)-2-((6-((4-bromobenzyl)amino)-9-isopropyl-*9H*-purin-2-yl)amino)butan-1-ol (30.00 mg, 0.069 mmol) was coupled with 4-pyridine boronic acid (10 mg, 0.083 mmol) according to general procedure **4**. The residue was purified by reverse phase column (30 → 45% MeOH in water (0.1% formic acid)) and concentrated *in vacuo* to afford the title compound (11 mg, 35%) as a white solid. LCMS purity >95%, ret. time 1.69 mins. HRMS (ESI +ve): $C_{24}H_{30}N_7O$ $[M+H]^+$: 432.2512 (Found: 432.2505). ^1H NMR (600 MHz, $CDCl_3$) δ 8.69 – 8.67 (m, 2H, H26), 7.63 (d, *J* = 8.5 Hz, 2H, H15), 7.55 (s, 1H, H8), 7.52 (d, *J* = 6.6 Hz, 4H, H14, H25)), 5.95 (s, 1H, H24), 4.92 – 4.80 (m, 3H, H10, H12), 4.63 (h, *J* = 6.8 Hz, 1H, H18), 3.95 – 3.89 (m, 1H, H20), 3.85 (dd, *J* = 10.7, 2.6 Hz, 1H, H21), 3.66 (dd, *J* = 10.7, 7.8 Hz, 1H, H21), 1.70 – 1.62 (m, 2H, H22), 1.57 (d, *J* = 7.1 Hz, 6H, H19), 1.05 (t, *J* = 7.5 Hz, 3H, H23). ^{13}C NMR (151 MHz, $CDCl_3$) δ 150.30 (C26), 147.84 (C17), 139.91 (C13), 137.19 (C16), 134.76 (C8), 128.42 (C25), 127.23 (C15), 121.54 (C14), 114.68 (C5), 68.62 (C21), 56.42 (C20), 46.51 (C12, C18), 25.04 (C22), 22.62 (C19), 22.55 (C19), 10.95 (C23).

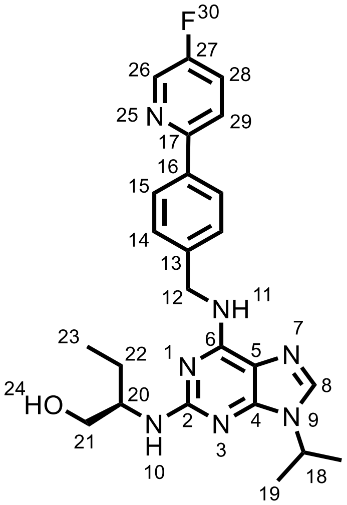

(R)-2-((6-((4-(5-fluoropyridin-2-yl)benzyl)amino)-9-isopropyl-*9H*-purin-2-yl)amino)butan-1-ol (6)

(2R)-2-[[9-isopropyl-6-[[4-(4,4,5,5-tetramethyl-1,3,2-dioxaborolan-2-yl)phenyl]methylamino]purin-2-yl]amino]butan-1-ol (35 mg, 0.07 mmol) was coupled with 2-Bromo-5-fluoropyridine (19 mg, 0.11 mmol) according to general procedure **4**. Conversion was incomplete so 2-Bromo-5-fluoropyridine

(12 mg, 0.07 mmol), potassium phosphate (30 mg, 0.15 mmol) and Pd(dppf)Cl_2.DCM, (3 mg, 0.004 mmol) were added and the reaction was heated to 100°C overnight. The reaction was cooled to rt, filtered through celite and purified by column chromatography (0 → 15% MeOH in DCM). The compound was further purified by reverse phase column (30 → 60% MeOH in water (0.1% formic acid)) and passed through an SCX cartridge (eluting with 2M NH_3/MeOH). Concentration *in vacuo* afforded the title compound (1.9 mg, 5%) as a white solid. LCMS purity >95%, ret. time 1.18 mins. HRMS (ESI +ve): $C_{24}H_{29}FN_7O$ $[M+H]^+$: 450.2418 (Found: 450.2360). ^1H NMR (500 MHz, $CDCl_3$) δ 8.55 (d, *J* = 2.9 Hz, 1H, H26), 7.93 (d, *J* = 8.3 Hz, 2H, H15), 7.76 – 7.70 (m, 1H, H29), 7.54 (s, 1H, H8), 7.49 (d, *J* = 8.4 Hz, 3H, H14, H28), 6.02 (s, 1H, H24), 4.93 – 4.77 (m, 3H, H10, H12), 4.67 – 4.60 (m, 1H, H18), 3.95 – 3.89 (m, 1H, H20), 3.85 (dd, *J* = 10.7, 2.6 Hz, 1H, H21), 3.65 (dd, *J* = 10.7, 7.9 Hz, 1H, H21), 1.68 – 1.58 (m, 2H, H22), 1.56 (d, *J* = 7.4 Hz, 6H, H19), 1.05 (t, *J* = 7.5 Hz, 3H, H23). ^{13}C NMR (126 MHz, $CDCl_3$) δ 159.08 (C2), 158.19 (C27), 154.89 (C6), 153.52 (C17), 150.34 (C4), 139.25 (C13), 137.91 (C16), 137.62 (C28), 134.69 (C8), 128.12 (C14), 127.00 (C15), 123.63 (C28), 121.30 (C29), 114.74 (C5), 68.60 (C21), 56.41 (C20), 46.49 (C18), 43.62 (C12), 25.04 (C22), 22.61 (C19), 22.55 (C19), 10.94 (C23).

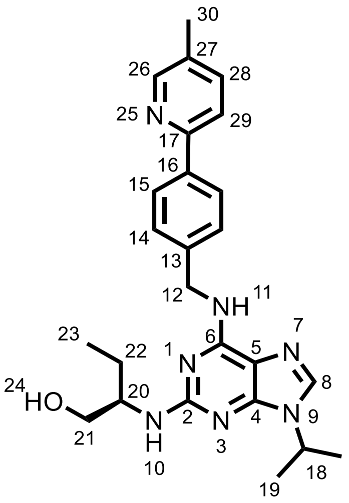

(R)-2-((9-isopropyl-6-((4-(5-methylpyridin-2-yl)benzyl)amino)-*9H*-purin-2-yl)amino)butan-1-ol (7)

(R)-2-((9-isopropyl-6-((4-(4,4,5,5-tetramethyl-1,3,2-dioxaborolan-2-yl)benzyl)amino)-*9H*-purin-2-yl)amino)butan-1-ol (30 mg, 0.06 mmol) was coupled with 2-Bromo-5-methylpyridine (16 mg, 0.09 mmol) according to general procedure **4**. Conversion was incomplete so 2-Bromo-5-methylpyridine (10 mg, 0.06 mmol), potassium phosphate (13 mg, 0.06 mmol) and Pd(dppf)Cl_2.DCM, (2.6 mg, 0.003 mmol) were added and the reaction was heated to 100°C under microwave irradiation for 2 hours. The reaction was filtered through celite, purified by reverse phase column (30 → 50% MeOH in water (0.1% formic acid)) and passed through an SCX cartridge (washing with MeOH and eluting with 2M NH_3/MeOH). Concentration *in vacuo* afforded the title compound (2 mg, 7%) as a white solid. LCMS purity >95%, ret. time 1.03 mins. HRMS (ESI +ve): $C_{25}H_{32}N_7O$ $[M+H]^+$: 446.2668 (Found: 446.2682). ^1H NMR (600 MHz, $CDCl_3$) δ 8.53 (s, 1H, H26), 7.95 (d, *J* = 8.2 Hz, 2H, H15), 7.63 (d, *J* = 8.1 Hz, 1H, H29), 7.57 (dd, *J* = 8.3, 2.1 Hz, 1H, H28), 7.53 (s, 1H, H8), 7.48 (d, *J* = 8.3 Hz, 2H, H14), 5.99 (s, 1H, H24), 4.90 (d, *J* = 5.9 Hz, 1H, H10), 4.85 (s, 2H, H12), 4.63 (hept, *J* = 6.8 Hz, 1H, H18), 3.95 – 3.89 (m, 1H, H20), 3.84 (dd, *J* = 10.7, 2.6 Hz, 1H, H21), 3.65 (dd, *J* = 10.7, 7.8 Hz, 1H, H21), 2.39 (s, 3H, H30), 1.70 – 1.58 (m, 2H, H22), 1.56 (dd, *J* = 6.7, 1.0 Hz, 6H, H19), 1.05 (t, *J* = 7.5 Hz, 3H, H23). ^{13}C

NMR (151 MHz, $CDCl_3$) δ 160.20 (C2), 154.87 (C6), 154.47 (C17), 150.09 (C4, C27), 139.19 (C13), 138.57 (C16), 137.31 (C28), 134.63 (C8), 131.61 (C27), 128.05 (C14), 126.91 (C15), 119.95 (C29), 114.81 (C5), 68.64 (C21), 56.42 (C20), 46.47 (C18), 44.18 (C12), 25.04 (C22), 22.62 (C19), 22.56 (C19), 18.18 (C30), 10.95 (C23).

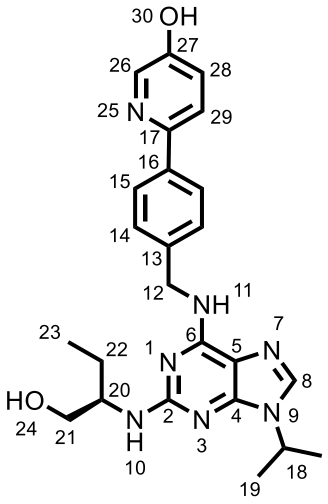

(R)-6-(4-(((2-((1-hydroxybutan-2-yl)amino)-9-isopropyl-*9H*-purin-6-yl)amino)methyl)phenyl)pyridin-3-ol (8)

((R)-2-((9-isopropyl-6-((4-(4,4,5,5-tetramethyl-1,3,2-dioxaborolan-2-yl)benzyl)amino)-*9H*-purin-2-yl)amino)butan-1-ol (30 mg, 0.06 mmol) was coupled with 2-Bromo-5-hydroxypyridine (16 mg, 0.09 mmol) according to general procedure **4**. Conversion was incomplete so 2-Bromo-5-hydroxypyridine (16 mg, 0.09 mmol), potassium phosphate (13 mg, 0.06 mmol) and Pd(dppf)Cl_2.DCM, (2.6 mg, 0.003 mmol) were added and the reaction was heated to 100°C under microwave irradiation for 2 hours. The reaction was filtered through celite, purified by reverse phase column (30 → 50% MeOH in water (0.1% formic acid)) and passed through an SCX cartridge (washing with MeOH and eluting with 2M NH_3/MeOH). Concentration *in vacuo* afforded the title compound (4.1 mg, 14%) as a white solid. LCMS purity >95%, ret. time 0.99 mins. HRMS (ESI +ve): $C_{24}H_{30}N_7O_2$ $[M+H]^+$: 448.2461 (Found: 448.2361). ^1H NMR (600 MHz, $CDCl_3$) δ 8.14 (d, *J* = 2.8 Hz, 1H, H26), 7.65 (s, 1H, H8), 7.55 (d, *J* = 8.0 Hz, 2H, H15), 7.16 (dd, *J* = 13.3, 8.3 Hz, 3H, H14, H29), 6.93 (dd, *J* = 8.6, 2.9 Hz, 1H, H28), 6.14 (s, 1H, H24), 4.99 (d, *J* = 6.2 Hz, 1H, H10), 4.80 – 4.65 (m, 3H, H12, H18), 4.00 – 3.94 (m, 1H, H20), 3.87 (dd, *J* = 10.8, 2.6 Hz, 1H, H21), 3.69 (dd, *J* = 10.7, 7.7 Hz, 1H, H21), 1.72 – 1.61 (m, 2H, H22), 1.60 (d, *J* = 6.8 Hz, 6H, H19), 1.06 (t, *J* = 7.4 Hz, 3H, H23). ^{13}C NMR (151 MHz, $CDCl_3$) δ 160.36 (C2), 154.36 (C6), 152.25 (C27), 150.22 (C4), 148.40 (C17), 138.24 (C16), 137.90 (C26), 137.62 (C13), 134.30 (C8), 127.75 (C14), 126.25 (C15), 122.93 (C28), 120.81 (C29), 113.62 (C5), 68.40 (C21), 56.3 (C20)1, 46.83 (C18), 44.14 (C12), 24.98 (C22), 22.59 (C19), 22.52 (C19), 10.93 (C23).

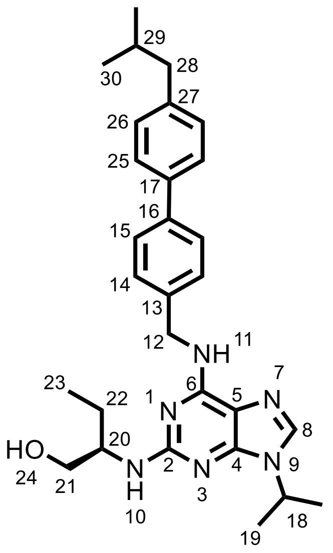

(R)-2-((6-(((4'-isobutyl-[1,1'-biphenyl]-4-yl)methyl)amino)-9-isopropyl-*9H*-purin-2-yl)amino)butan-1-ol (9)

(R)-2-((6-((4-bromobenzyl)amino)-9-isopropyl-*9H*-purin-2-yl)amino)butan-1-ol (30 mg, 0.07 mmol) was coupled with 4-Isobutylphenylboronic acid (15 mg, 0.08 mmol) according to general procedure **4**. Conversion was incomplete so 4-Isobutylphenylboronic acid (15 mg, 0.08 mmol), potassium phosphate (44 mg, 0.2 mmol) and Pd(dppf)Cl_2.DCM (2.8 mg, 0.004 mmol) were added the reaction was heated to 100°C under microwave irradiation for 2 hours. mmol). The reaction was filtered through celite, purified by reverse phase column (60 → 80% MeOH in water (0.1% formic acid)). Concentration in vacuo afforded the title compound (9.5 mg, 27%) as a white solid. LCMS purity >95%, ret. time 1.54 mins. HRMS (ESI +ve $C_{29}H_{39}N_6O$ $[M+H]^+$: 487.3185 (Found: 487.3174). ^{1}H NMR (600 MHz, $CDCl_3$) δ 7.57 (d, *J* = 7.9 Hz, 2H, H14), 7.52 – 7.49 (m, 3H, H25, H8), 7.44 (d, *J* = 7.8 Hz, 2H, H15), 7.23 (d, *J* = 7.8 Hz, 2H, H26), 6.12 (s, 1H, H24), 4.92 (d, *J* = 6.0 Hz, 1H, H10), 4.82 (s, 2H, H12), 4.62 (h, *J* = 6.8 Hz, 1H, H18), 3.96 – 3.89 (m, 1H, H20), 3.85 (dd, *J* = 10.7, 2.5 Hz, 1H, H21), 3.66 (dd, *J* = 10.7, 7.9 Hz, 1H, H21), 2.53 (d, *J* = 7.2 Hz, 2H, H28), 1.97 – 1.88 (m, 1H, H29), 1.70 – 1.57 (m, 2H, H22), 1.55 (d, *J* = 6.9 Hz, 6H, H19), 1.05 (t, *J* = 7.5 Hz, 3H, H23), 0.96 (d, *J* = 6.7 Hz, 6H, H30). ^{13}C NMR (151 MHz, $CDCl_3$) δ 160.09 (C2), 154.89 (C6), 150.12 (C4), 140.90 (C27), 140.29 (C13), 138.15 (C17), 137.50 (C16), 134.61 (C8), 129.55 (C26), 128.12 (C15), 127.15 (C14), 126.73 (C25), 114.76 (C5), 68.61 (C21), 56.41 (C20), 46.44 (C18), 45.08 (C28), 44.11 (C12), 30.25 (C29), 25.04 (C22), 22.61 (C19), 22.55 (C19), 22.43 (C30), 10.96 (C23).

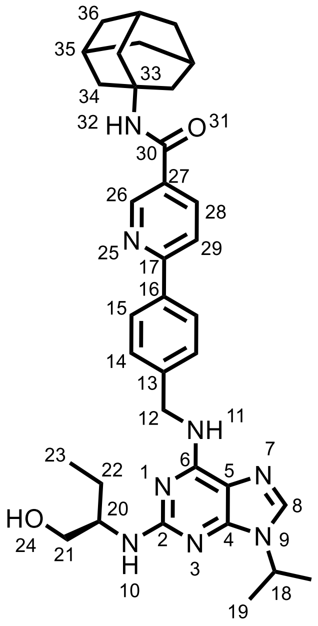

(R)-N-(adamantan-1-yl)-6-(4-(((2-((1-hydroxybutan-2-yl)amino)-9-isopropyl-9H-purin-6-yl)amino)methyl)phenyl)nicotinamide (10)

(2R)-2-[[9-isopropyl-6-[[4-(4,4,5,5-tetramethyl-1,3,2-dioxaborolan-2-yl)phenyl]methylamino]purin-2-yl]amino]butan-1-ol (40 mg, 0.083 mmol) was coupled N-(adamantan-1-yl)-6-bromonicotinamide (33 mg, 0.10 mmol) according to general procedure **4**. The residue was purified by reverse phase column (50 → 80% MeOH in water (0.1% formic acid)) and the product was passed through an SCX cartridge (washing with MeOH and eluting with 2M NH3/MeOH). Concentration *in vacuo* afforded the title compound (35 mg, 66%) as a brown solid. LCMS purity >95%, ret. time 1.43 mins. HRMS (ESI +ve): $C_{35}H_{45}N_8O_2$ $[M+H]^+$: 609.3659 (Found: 609.3659). ^{1}H NMR (600 MHz, $CDCl_3$) δ 8.98 (d, *J* = 2.3 Hz, 1H, H26), 8.12 (dd, *J* = 8.3, 2.3 Hz, 1H, H28), 8.00 (d, *J* = 8.3 Hz, 2H, H15), 7.77 (d, *J* = 8.3 Hz, 1H, H29), 7.53 – 7.48 (m, 3H, H14, H8), 6.16 (s, 1H, H32), 5.85 (s, 1H, H32), 4.93 – 4.76 (m, 3H, H10, H12), 4.62 (hept, *J* = 6.8 Hz, 1H, H18), 3.94 – 3.87 (m, 1H, H20), 3.83 (dd, *J* = 10.7, 2.6 Hz, 1H, H21), 3.64 (dd, *J* = 10.7, 7.7 Hz, 1H, H21), 2.17 (s, 9H, H34, H35), 1.77 – 1.74 (m, 6H, H36), 1.69 – 1.57 (m, 2H, H22), 1.55 (d, *J* = 6.8 Hz, 6H, H19), 1.03 (t, *J* = 7.5 Hz, 3H, H23). ^{13}C NMR (151 MHz, $CDCl_3$) δ 164.65 (C30), 160.05 (C2), 159.11 (C27), 154.85 (C6), 150.46 (C4), 147.67 (C26), 140.58 (C13), 137.44 (C16), 135.78 (C29), 134.66 (C8), 129.60 (C17), 128.11 (C14), 127.38 (C15), 119.90 (C29), 114.73 (C5), 68.50 (C21), 56.35 (C20), 52.73 (C33), 46.48 (C18), 44.01 (C12), 41.69 (C34), 36.33 (C36), 29.50 (C35), 25.01 (C22), 22.61 (C19), 10.94 (C23).

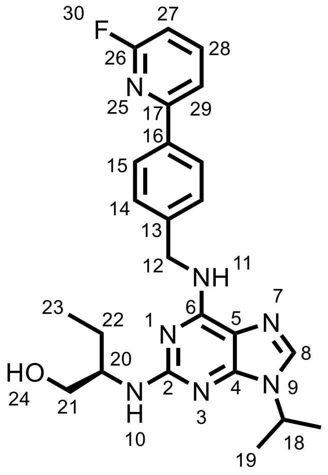

(2R)-2-[[6-[[4-(6-fluoro-2-pyridyl)phenyl]methylamino]-9-isopropyl-purin-2-yl]amino]butan-1-ol (11)

(2R)-2-[[9-isopropyl-6-[[4-(4,4,5,5-tetramethyl-1,3,2-dioxaborolan-2-yl)phenyl]methylamino]purin-2-yl]amino]butan-1-ol (40 mg, 0.08 mmol) was coupled with 2-bromo-6-fluoro-pyridine (22 mg, 0.12 mmol) according to general procedure **4**. The residue was purified by reverse phase column (30 → 60% MeOH in water (0.1% formic acid) and the desired fractions were passed through an SCX cartridge (washing with MeOH and eluting with 2M NH_3/MeOH). Concentration *in vacuo* afforded the title compound (17 mg, 45%) as a white solid. LCMS purity >95%, ret. time 1.21 mins. HRMS (ESI +ve): $C_{24}H_{29}FN_7O$ $[M+H]^+$: 450.2418 (Found: 450.2414). ^1H NMR (600 MHz, $CDCl_3$) δ 8.01 – 7.98 (m, 2H, H15), 7.86 (q, J = 8.0 Hz, 1H, H28), 7.63 (dd, J = 7.6, 2.5 Hz, 1H, H27), 7.56 (s, 1H, H8), 7.50 (d, J = 8.1 Hz, 2H, H14), 6.88 (dd, J = 8.1, 3.0 Hz, 1H, H29), 4.88 (s, 2H, H12), 4.65 (hept, J = 6.8 Hz, 1H, H18), 3.95 (s, 1H, H20), 3.84 (dd, J = 10.8, 2.8 Hz, 1H, H21), 3.66 (dd, J = 10.8, 7.7 Hz, 1H, H21), 1.71 – 1.59 (m, 2H H22), 1.57 (dd, J = 6.8, 1.5 Hz, 6H, H19), 1.05 (t, J = 7.4 Hz, 3H, H23). ^{13}C NMR (151 MHz, $CDCl_3$) δ 164.20 (C26), 162.62 (C17), 159.76 (C2), 155.94 (d, J = 13.4 Hz, C6), 141.64 (d, J = 7.6 Hz, C5), 144.90 (C28), 140.21 (C13), 136.71 (C16), 134.89 (C8), 128.10 (C14), 127.19 (C15), 117.21 (d, J = 3.9 Hz, C27), 113.33 (C4), 107.68 (d, J = 37.6 Hz, C29), 68.73 (C21), 56.37 (C20), 46.62 (C18), 44.15 (C12) 24.97 (C22), 22.57 (d, J = 9.9 Hz, C19), 10.92 (C23).

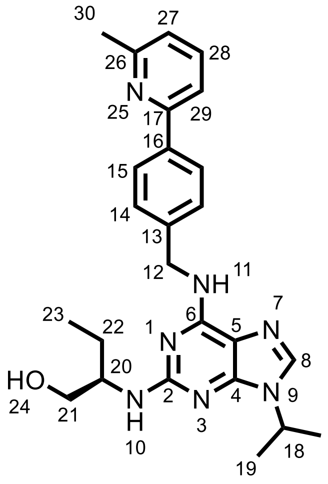

(2R)-2-[[9-isopropyl-6-[[4-(6-methyl-2-pyridyl)phenyl]methylamino]purin-2-yl]amino]butan-1-ol (12)

(2R)-2-[[9-isopropyl-6-[[4-(4,4,5,5-tetramethyl-1,3,2-dioxaborolan-2-yl)phenyl]methylamino]purin-2-yl]amino]butan-1-ol (40 mg, 0.08 mmol) was coupled with 2-bromo-6-methylpyridine (21 mg, 0.12 mmol) according to general procedure **4**. The residue was purified by reverse phase column (30 → 60% MeOH in water (0.1% formic acid) and the desired fractions were passed through an SCX cartridge (washing with MeOH and eluting with 2M NH_3/MeOH). Concentration *in vacuo* afforded the title compound (5.9 mg, 16%) as a white solid. LCMS purity >95%, ret. time 0.99 mins. HRMS (ESI +ve): $C_{25}H_{32}N_7O$ $[M+H]^+$: 446.2668 (Found: 446.2663). ^{1}H NMR (600 MHz, $CDCl_3$) δ 7.96 (d, J = 8.1 Hz, 2H, H15), 7.64 (t, J = 7.7 Hz, 1H, H28), 7.53 – 7.50 (m, 2H, H8, H29), 7.47 (d, J = 8.0 Hz, 2H, H14), 7.10 (d, J = 7.6 Hz, 1H, H27), 4.99 (s, 1H, H11), 4.84 (s, 1H, H12), 4.63 (hept, J = 6.8 Hz, 1H, H18), 3.92 (qd, J = 8.1, 6.8, 4.1 Hz, 1H, H20), 3.84 (dd, J = 10.7, 2.7 Hz, 1H, H21), 3.65 (dd, J = 10.7, 7.7 Hz, 1H, H21), 2.64 (s, 3H, H30), 1.70 – 1.57 (m, 2H, H22), 1.56 (s, 6H, H19), 1.04 (t, J = 7.4 Hz, 3H, H23). ^{13}C NMR (151 MHz, CDCl3) δ 160.21 (C2), 158.38 (C26), 156.61 (C17), 154.74 (C6), 149.81 (C4), 139.26 (C13), 138.93 (C16), 136.88 (C28), 134.68 (C29), 128.01 (C14), 127.24 (C15), 121.60 (C27), 117.52 (C8), 114.64 (C5), 68.39 (C21), 56.35 (C20), 46.52 (C18), 43.82 (C12), 25.00 (C30), 24.74 (C22), 22.60 (C19), 22.53 (C19), 10.93 (C23).

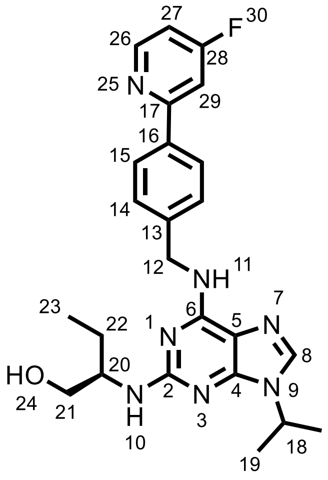

(R)-2-((6-((4-(4-fluoropyridin-2-yl)benzyl)amino)-9-isopropyl-*9H*-purin-2-yl)amino)butan-1-ol (13)

(R)-2-((9-isopropyl-6-((4-(4,4,5,5-tetramethyl-1,3,2-dioxaborolan-2-yl)benzyl)amino)-*9H*-purin-2-yl)amino)butan-1-ol (50 mg, 0.10 mmol) was coupled with 2-Bromo-4-fluoropyridine (0.02 mL, 0.16 mmol) according to general procedure **4**. Conversion was incomplete so 2-Bromo-4-fluoropyridine (18 mg, 0.10 mmol), potassium phosphate (22 mg, 0.10 mmol) and Pd(dppf)Cl_2.DCM (4.3 mg, 0.005 mmol) were added the reaction was heated to 100°C under microwave irradiation for 2 hours. The reaction was filtered through celite, purified by reverse phase column (40 → 60% MeOH in water (0.1% formic acid)). Further purification by preparative HPLC was performed and the desired fractions were passed through an SCX cartridge (washing with MeOH and eluting with 2M NH_3/MeOH). Concentration *in vacuo* afforded the title compound (3.2 mg, 6%) as a white solid. LCMS purity >95%, ret. time 1.15 mins. HRMS (ESI +ve): $C_{24}H_{29}FN_7O$ $[M+H]^+$: 450.2418 (Found: 450.2396). ^{1}H NMR (600 MHz, $CDCl_3$) δ 8.66 (dd, *J* = 8.8, 5.6 Hz, 1H, H26), 7.96 (d, *J* = 8.3 Hz, 2H, H15), 7.53 (s, 1H, H8), 7.50 (d, *J* = 8.2 Hz, 2H, H14), 7.45 (dd, *J* = 10.4, 2.4 Hz, 1H, H29), 7.01 – 6.97 (m, 1H, H27), 6.07 (s, 1H, H24), 4.91 (d, *J* = 6.0 Hz, 1H, H10), 4.86 (s, 2H, H12), 4.63 (hept, *J* = 6.8 Hz, 1H, H18), 3.94 – 3.89 (m, 1H, H20), 3.84 (dd, *J* = 10.7, 2.6 Hz, 1H, H21), 3.65 (dd, *J* = 10.7, 7.8 Hz, 1H, H21), 1.69 – 1.58 (m, 2H, H22), 1.56 (d, *J* = 6.8, 1.0 Hz, 6H, H19), 1.04 (t, *J* = 7.5 Hz, 3H, H23). ^{13}C NMR (151 MHz, $CDCl_3$) δ 169.40 (d, *J* = 261.1 Hz, C28), 160.32 (d, *J* = 7.0 Hz, C17), 160.03 (C2), 154.84 (C6), 151.96 (d, *J* = 7.1 Hz, C26), 150.40 (C4), 140.48 (C13), 137.40 (d, *J* = 3.3 Hz, C16), 134.70 (C8), 128.12 (C14), 127.18 (C15), 114.75 (C5), 109.94 (d, *J* = 16.5 Hz, C27), 108.04 (d, *J* = 17.5 Hz, C29), 68.55 (C21), 56.39 (C20), 46.49 (C18), 25.02 (C22), 22.61 (C19), 22.55 (C19), 10.95 (C23).

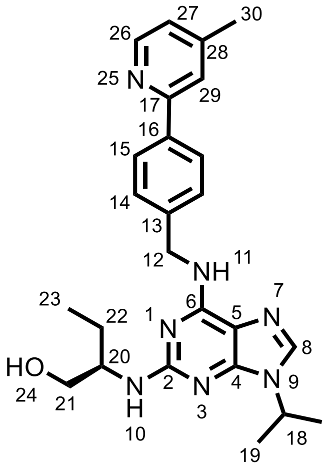

(R)-2-((9-isopropyl-6-((4-(4-methylpyridin-2-yl)benzyl)amino)-9H-purin-2-yl)amino)butan-1-ol (14)

(R)-2-((9-isopropyl-6-((4-(4,4,5,5-tetramethyl-1,3,2-dioxaborolan-2-yl)benzyl)amino)-*9H*-purin-2-yl)amino)butan-1-ol (30 mg, 0.06 mmol) was coupled with 2-bromo-4-methylpyridine (0.01 mL, 0.09 mmol) according to general procedure **4**. The residue was purified by reverse phase column (30 → 50% MeOH in water (0.1% formic acid)). Further purification by preparative HPLC was performed and the desired fractions were passed through an SCX cartridge (washing with MeOH and eluting with 2M NH_3/MeOH). Concentration *in vacuo* afforded the title compound (4.5 mg, 16%) as a white solid. LCMS purity >95%, ret. time 1.20 mins. HRMS (ESI +ve): $C_{25}H_{32}N_7O$ $[M+H]^+$: 446.2662 (Found: 446.2696). ^1H NMR (600 MHz, $CDCl_3$) δ 8.55 (d, *J* = 5.0 Hz, 1H, H26), 7.96 (d, *J* = 8.3 Hz, 2H, H15), 7.55 (s, 1H, H29), 7.53 (s, 1H, H8), 7.48 (d, *J* = 8.2 Hz, 2H, H14), 7.07 (d, *J* = 5.1 Hz, 1H, H27), 6.08 (s, 1H, H24), 5.00 – 4.90 (m, 1H, H10), 4.85 (s, 2H, H12), 4.66 – 4.60 (m, 1H, H18), 3.97 – 3.89 (m, 1H, H20), 3.85 – 3.81 (m, 1H, H21), 3.65 (dd, *J* = 10.7, 7.8 Hz, 1H, H21), 2.43 (s, 3H, H30), 1.70 – 1.58 (m, 2H, H22), 1.56 (d, *J* = 6.8, 1.0 Hz, 6H, H19), 1.04 (t, *J* = 7.5 Hz, 3H, H23). ^{13}C NMR (151 MHz, $CDCl_3$) δ 160.02 (C2), 157.01 (C17), 154.83 (C6), 150.20 (C4), 149.43 (C26), 147.75 (C28), 139.43 (C13), 138.66 (C16), 134.66 (C8), 128.02 (C14), 127.15 (C15), 123.14 (C27), 121.44 (C29), 114.74 (C5), 68.54 (C21), 56.39 (C20), 46.48 (C18), 44.24 (C12), 25.02 (C22), 22.61 (C19), 22.55 (C19), 21.25 (C30), 10.95 (C23).

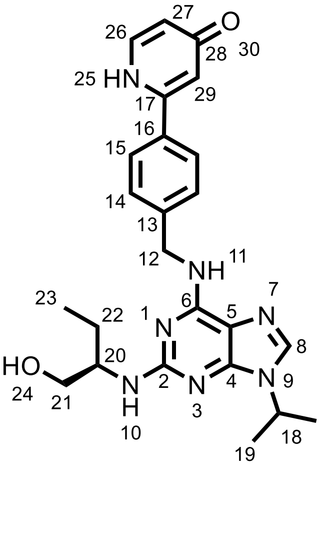

(R)-2-(4-(((2-((1-hydroxybutan-2-yl)amino)-9-isopropyl-*9H*-purin-6-yl)amino)methyl)phenyl)pyridin-4-ol (15)

(((R)-2-((9-isopropyl-6-((4-(4,4,5,5-tetramethyl-1,3,2-dioxaborolan-2-yl)benzyl)amino)-*9H*-purin-2-yl)amino)butan-1-ol (40 mg, 0.08 mmol) was coupled with 2-Bromo-4-hydroxypyridine (21 mg, 0.12 mmol) according to general procedure **4**. Conversion was incomplete so 2-Bromo-5-hydroxypyridine (15 mg, 0.08 mmol), potassium phosphate (35 mg, 0.16 mmol) and Pd(dppf)Cl_2.DCM, (3.4 mg, 0.004 mmol) were added and the reaction was heated to 100°C under microwave irradiation for 2 hours. The reaction was filtered through celite, purified by reverse phase column (40 → 60% MeOH in water (0.1% formic acid)) and passed through an SCX cartridge (washing with MeOH and eluting with 2M NH_3/MeOH). Concentration *in vacuo* afforded the title compound (30 mg, 76%) as a white solid. LCMS purity >95%, ret. time 1.25 mins. HRMS (ESI +ve): $C_{24}H_{30}N_7O_2$ $[M+H]^+$: 448.2461 (Found: 448.2449). ^{1}H NMR (600 MHz, $CDCl_3$) δ 7.66 (d, *J* = 7.6 Hz, 1H, H26), 7.54 (s, 1H, H8), 7.35 (d, *J* = 7.8 Hz, 2H, H15), 7.20 (d, *J* = 7.9 Hz, 2H, H14), 6.56 – 6.48 (m, 2H, H29, H30), 6.37 (d, *J* = 7.0, 2.4 Hz, 1H, H27), 5.19 (s, 1H, H23), 4.76 – 4.49 (m, 4H, H10, H12, H18), 3.97 – 3.90 (m, 1H, H20), 3.82 (dd, *J* = 10.9, 3.0 Hz, 1H, H21), 3.62 (dd, *J* = 10.7, 7.5 Hz, 1H, H21), 1.67 – 1.54 (m, 2H, H22), 1.53 – 1.49 (m, 6H, H19), 1.01 (t, *J* = 7.4 Hz, 3H, H23). ^{13}C NMR (151 MHz, $CDCl_3$) δ 159.92 (C2), 154.53 (C6), 150.00 (C4), 140.96 (C13), 139.46 (C26), 134.58 (C8), 133.28 (C16), 127.97 (C14), 126.94 (C15), 115.77 (C27), 114.74 (C29), 114.29 (C5), 67.59 (C21), 55.88 (C20), 46.57 (C18), 43.75 (C12), 24.85 (C22), 22.56 (C19), 22.49 (C19), 10.92 (C23).

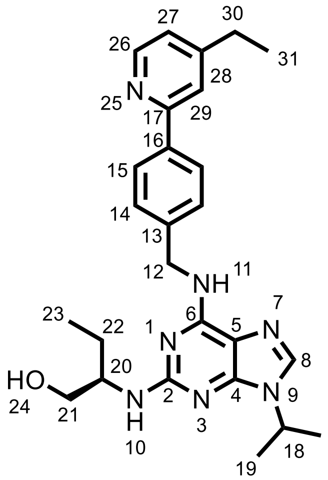

(R)-2-((6-((4-(4-ethylpyridin-2-yl)benzyl)amino)-9-isopropyl-9H-purin-2-yl)amino)butan-1-ol (16)

(R)-2-((9-isopropyl-6-((4-(4,4,5,5-tetramethyl-1,3,2-dioxaborolan-2-yl)benzyl)amino)-*9H*-purin-2-yl)amino)butan-1-ol (40 mg, 0.08 mmol) was coupled with 2-bromo-4-ethylpyridine (0.015 mL, 0.12 mmol) according to general procedure **4**. The residue was purified by reverse phase column (25 → 60% MeOH in water (0.1% formic acid)). Further purification by preparative HPLC was performed and the desired fractions were passed through an SCX cartridge (washing with MeOH and eluting with 2M NH_3/MeOH). Concentration *in vacuo* afforded the title compound (10 mg, 28%) as a yellow solid. LCMS purity >95%, ret. time 1.07 mins. HRMS (ESI +ve): $C_{26}H_{34}N_7O$ $[M+H]^+$: 460.2825 (Found: 460.2809). ^{1}H NMR (600 MHz, $CDCl_3$) δ 8.57 (d, J = 5.0 Hz, 1H, H26), 7.95 (dd, J = 8.1, 2.2 Hz, 2H, H15), 7.55 (s, 1H, H29), 7.51 (d, J = 3.8 Hz, 1H, H8), 7.48 (d, J = 8.0 Hz, 2H, H14), 7.09 (dd, J = 5.1, 1.6 Hz, 1H, H27), 4.83 (s, 1=2H, H12), 4.62 (p, J = 6.9 Hz, 1H, H18), 3.92 (d, J = 8.5 Hz, 1H, H20), 3.82 (dd, J

= 10.9, 2.7 Hz, 1H, H21), 3.64 (dd, J = 10.8, 7.5 Hz, 1H, H21), 2.72 (q, J = 7.7 Hz, 2H, H30), 1.69 – 1.55 (m, 2H, H22), 1.54 (d, J = 6.8 Hz, 6H, H19), 1.31 (t, J = 7.8 Hz, 3H, H31), 1.02 (t, J = 7.4 Hz, 3H, H23). ^{13}C NMR (151 MHz, $CDCl_3$) δ 166.49 (C2), 157.13 (C17), 154.71 (C6), 153.80 (C4, C28), 149.48 (C26), 139.48 (C13), 138.66 (C16), 134.53 (C8), 127.98 (C14), 127.18 (C15), 121.90 (C27), 120.29 (C29), 114.28 (C5), 68.06 (C21), 56.22 (C20), 46.56 (C18), 44.05 (C12), 28.44 (C30), 24.91 (C22), 22.57 (C19), 22.51 (C19), 14.46 (C31), 10.91 (C23).

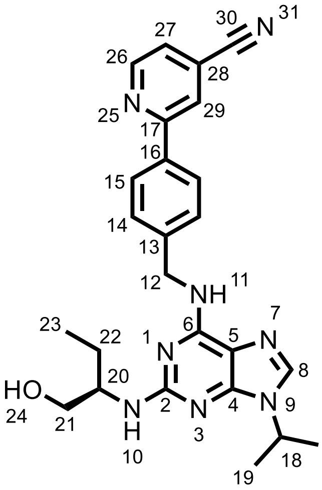

(R)-2-(4-(((2-((1-hydroxybutan-2-yl)amino)-9-isopropyl-*9H*-purin-6-yl)amino)methyl)phenyl)isonicotinonitrile (17)

(2R)-2-[[9-isopropyl-6-[[4-(4,4,5,5-tetramethyl-1,3,2-dioxaborolan-2-yl)phenyl]methylamino]purin-2-yl]amino]butan-1-ol (40 mg, 0.083 mmol) was coupled 2-Bromoisonicotinonitrile (18 mg, 0.10 mmol) according to general procedure **4**. The residue was purified by reverse phase column (30 → 60% MeOH in water (0.1% formic acid)) and the product was passed through an SCX cartridge (washing with MeOH and eluting with 2M NH_3/MeOH). Concentration in vacuo afforded the title compound (16 mg, 40%) as a yellow solid. LCMS purity >95%, ret. time 1.17 mins. HRMS (ESI +ve): $C_{25}H_{29}N_8O$ $[M+H]^+$: 457.2464 (Found: 457.2453). ^{1}H NMR (600 MHz, $CDCl_3$) δ 8.84 (dd, *J* = 5.0, 0.9 Hz, 1H, H26), 7.95 (d, *J* = 8.3 Hz, 2H, H15), 7.92 (s, 1H, H29), 7.51 – 7.47 (m, 3H, H8, H14), 7.43 (dd, *J* = 5.0, 1.4 Hz, 1H, H27), 6.47 (s, 1H, H24), 4.96 (d, *J* = 6.3 Hz, 1H, H10), 4.88 – 4.75 (m, 2H, H12), 4.60 (h, *J* = 6.8 Hz, 1H, H18), 3.93 – 3.87 (m, 1H, H210), 3.82 (dd, *J* = 10.7, 2.7 Hz, 1H, H21), 3.63 (dd, *J* = 10.7, 7.6 Hz, 1H, H21), 1.68 – 1.56 (m, 2H, H22), 1.53 (d, *J* = 6.8 Hz, 6H, H19), 1.01 (t, *J* = 7.5 Hz, 3H, H23). ^{13}C NMR (151 MHz, $CDCl_3$) δ 160.01 (C2), 158.38 (C17), 154.83 (C6), 150.60 (C26), 150.43 (C4), 141.38 (C13), 136.25 (C16), 134.65 (C8), 128.22 (C14), 127.14 (C15), 123.09 (C27), 121.90 (C29), 121.18 (C28), 116.74 (C30), 114.63 (C5), 68.16 (C21), 56.21 (C20), 46.46 (C18), 43.97 (C12), 24.96 (C22), 22.58 (C19), 22.51 (C19), 10.93 (C23).

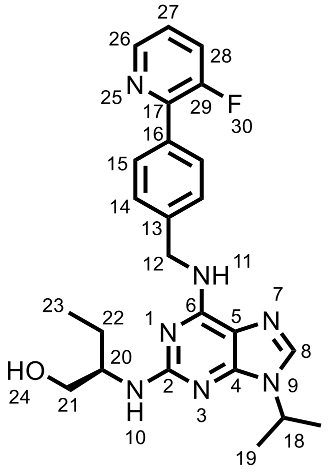

(2R)-2-[[6-[[4-(3-fluoro-2-pyridyl)phenyl]methylamino]-9-isopropyl-purin-2-yl]amino]butan-1-ol (18)

(2R)-2-[[9-isopropyl-6-[[4-(4,4,5,5-tetramethyl-1,3,2-dioxaborolan-2-yl)phenyl]methylamino]purin-2-yl]amino]butan-1-ol (40 mg, 0.08 mmol) was coupled with 2-bromo-3-fluoro-pyridine (21 mg, 0.12 mmol) according to general procedure **4**. The reaction was filtered through celite, purified by reverse phase column (30 → 60% MeOH in water (0.1% formic acid)). Further purification by preparative HPLC was performed and the desired fractions were passed through an SCX cartridge (washing with MeOH and eluting with 2M NH_3/MeOH). Concentration *in vacuo* afforded the title compound (24 mg, 65%) as a white solid. LCMS purity >95%, ret. time 1.18 mins. HRMS (ESI +ve): $C_{24}H_{29}FN_7O$ $[M+H]^+$: 450.2418 (Found: 450.2411). ^{1}H NMR (600 MHz, $CDCl_3$) δ 8.53 (dt, J = 4.6, 1.6 Hz, 1H, H26), 7.98 (dd, J = 8.3, 1.7 Hz, 2H, H15), 7.56 (s, 1H, H8), 7.54 – 7.47 (m, 3H, H14, H28), 7.30 – 7.26 (m, 1H, H27), 4.88 (s, 1H, H12), 4.65 (p, J = 6.7 Hz, 1H, H18), 3.95 (s, 1H, H20), 3.83 (dd, J = 10.9, 2.8 Hz, 1H, H21), 3.66 (dd, J = 10.8, 7.6 Hz, 1H, H21), 1.65 (ddt, J = 37.8, 14.6, 7.0 Hz, 2H, H22), 1.57 (dd, J = 6.8, 1.5 Hz, 6H, H19), 1.05 (t, J = 7.5 Hz, 3H, H23). ^{13}C NMR (151 MHz, $CDCl_3$) δ 158.42 (C2, C29), 156.85 (C17), 154.37 (C6), 150.43 (C4), 145.40 (d, J = 5.1 Hz, C26), 139.37 (C13), 136.00 (C16), 134.61 (C8), , 129.10 (d, J = 5.9 Hz, C15), 127.75 (C14), 124.10 (d, J = 20.7 Hz, C28), 123.45 (d, J = 3.8 Hz, C27), 114.63 (C5), 68.31 (C21), 56.35 (C20), 46.13 (C18), 44.32 (C12), 24.91 (C22), 22.59 (C19), 22.52 (C19), 10.87 (C23).

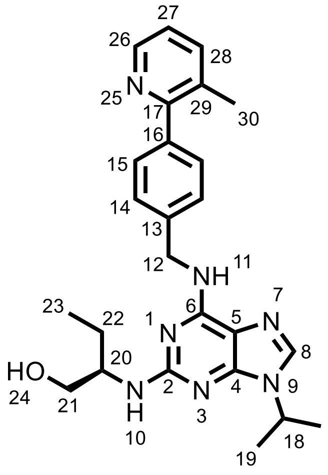

(2R)-2-[[9-isopropyl-6-[[4-(3-methyl-2-pyridyl)phenyl]methylamino]purin-2-yl]amino]butan-1-ol (19)

(R)-2-((9-isopropyl-6-((4-(4,4,5,5-tetramethyl-1,3,2-dioxaborolan-2-yl)benzyl)amino)-*9H*-purin-2-yl)amino)butan-1-ol (50 mg, 0.10 mmol) was coupled with 2-bromo-3-methylpyridine (0.017 mL, 0.16 mmol) according to general procedure **4**. The residue was purified by reverse phase column (30 → 60% MeOH in water (0.1% formic acid)) and the desired fractions were passed through an SCX cartridge (washing with MeOH and eluting with 2M NH_3/MeOH). Concentration *in vacuo* afforded the title compound (10 mg, 22%) as a white solid. LCMS purity >95%, ret. time 1.06 mins. HRMS (ESI +ve): $C_{25}H_{32}N_7O$ $[M+H]^+$: 446.2668 (Found: 446.2667). ^1H NMR (600 MHz, $CDCl_3$) δ 8.53 (dd, J = 4.8, 1.6 Hz, 1H, H26), 7.59 (d, J = 7.6 Hz, 1H, H28), 7.54 – 7.50 (m, 3H, H8, H15), 7.47 (d, J = 8.0 Hz, 2H, H14), 7.19 (dd, J = 7.6, 4.7 Hz, 1H, H27), 4.93 (s, 1H, H11), 4.85 (s, 2H, H12), 4.64 (h, J = 6.8 Hz, 1H, H18), 3.95 – 3.88 (m, 1H, H20), 3.82 (dd, J = 10.7, 2.8 Hz, 1H, H21), 3.63 (dd, J = 10.7, 7.7 Hz, 1H, H21), 2.37 (s, 3H, H30), 1.70 – 1.57 (m, 2H, H22), 1.56 (d, J = 6.8 Hz, 6H, H19), 1.05 (t, J = 7.5 Hz, 3H, H23). ^{13}C NMR (151 MHz, $CDCl_3$) δ 158.38 (C2, C17), 154.80 (C6), 150.15 (C4), 146.98 (C26), 139.74 (C13), 138.50 (C28), 138.46 (C16), 134.65 (C8), 130.85 (C29), 129.24 (C14), 127.49 (C15), 122.08 (C27), 114.86 (C5), 68.39 (C21), 56.33 (C20), 46.48 (C18), 44.36 (C12), 25.00 (C22), 22.62 (C19), 22.56 (C19), 20.09 (C30), 10.92 (C23).

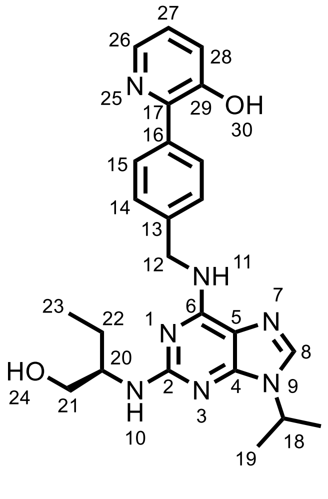

2-[4-[[[2-[[(1R)-1-(hydroxymethyl)propyl]amino]-9-isopropyl-purin-6-yl]amino]methyl]phenyl]pyridin-3-ol (20)

(2R)-2-[[9-isopropyl-6-[[4-(4,4,5,5-tetramethyl-1,3,2-dioxaborolan-2-yl)phenyl]methylamino]purin-2-yl]amino]butan-1-ol (50 mg, 0.10 mmol) was coupled with 3-benzyloxy-2-bromo-pyridine (41 mg, 0.16 mmol) according to general procedure **4.** purified by reverse phase column (30 → 60% MeOH in water (0.1% formic acid) and the desired fractions were passed through an SCX cartridge (washing with MeOH and eluting with 2M NH_3/MeOH). Concentration *in vacuo* afforded (2R)-2-[[6-[[4-(3-benzyloxy-2-pyridyl)phenyl]methylamino]-9-isopropyl-purin-2-yl]amino]butan-1-ol (31 mg, 55%) as a white solid. To a degassed (N_2) solution of (2R)-2-[[6-[[4-(3-benzyloxy-2-pyridyl)phenyl]methylamino]-9-isopropyl-purin-2-yl]amino]butan-1-ol2R)-2-[[9-isopropyl-6-[[4-

(4,4,5,5-tetramethyl-1,3,2-dioxaborolan-2-yl)phenyl]methylamino]purin-2-yl]amino]butan-1-ol (25 mg, 0.05 mmol) in ethanol (1 mL, 0.05 M) was added Palladium (11 mg, 0.005 mmol) (10% on carbon). The solution was purged with hydrogen 3x before leaving under a H_2 atmosphere at rt for 16 hours. The reaction was filtered through celite, and the product purified by column chromatography (0 → 20% MeOH in DCM). Concentration *in vacuo* afforded the title compound (10 mg, 50%) as a yellow solid. LCMS purity >95%, ret. time 0.89 mins. HRMS (ESI +ve): $C_{24}H_{30}N_7O_2$ $[M+H]^+$: 448.2448 (Found: 448.2448). ^{1}H NMR (600 MHz, $CDCl_3$) δ 8.23 (dd, J = 4.7, 1.4 Hz, 1H, H26), 7.72 (d, J = 7.8 Hz, 2H, H15), 7.60 (s, 1H, H8), 7.30 (d, J = 8.1 Hz, 1H, H28), 7.26 (d, J = 7.8 Hz, 2H, H14), 7.12 (dd, J = 8.2, 4.6 Hz, 1H, H27), 4.79 – 4.57 (m, 3H, H12, H18), 3.95 – 3.88 (m, 1H, H20), 3.75 (dd, J = 10.9, 2.8 Hz, 1H, H21), 3.59 – 3.54 (m, 1H, H21), 1.66 – 1.51 (m, 9H, H19, H22), 1.02 (t, J = 7.4 Hz, 3H, H23). 13C NMR (151 MHz, CDCl3) δ 159.79 (C2), 153.89 (C6), 150.98 (C29), 149.91 (C4), 146.21 (C17), 141.00 (C26), 138.47 (C13), 136.40 (C16), 134.09 (C8), 129.25 (C15), 127.18 (C14), 124.17 (C28), 123.22 (C27), 113.81 (C5), 68.01 (C21), 55.90 (C20), 46.72 (C18), 44.24 (C12), 24.87 (C22), 22.59 (C19), 22.50 (C19), 10.85 (C23).

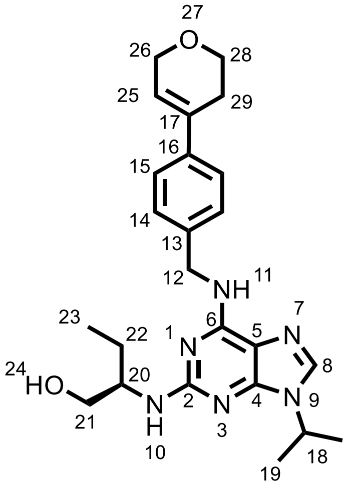

(R)-2-((6-((4-(3,6-dihydro-*2H*-pyran-4-yl)benzyl)amino)-9-isopropyl-*9H*-purin-2-yl)amino)butan-1-ol (21)

((R)-2-((6-((4-bromobenzyl)amino)-9-isopropyl-*9H*-purin-2-yl)amino)butan-1-ol (30 mg, 0.07 mmol) was coupled with 3,6-Dihydro-*2H*-pyran-4-boronic acid pinacol ester (17 mg, 0.08 mmol) according to general procedure **4**. The residue was purified by reverse phase chromatography (30 → 60% MeOH in water (0.1% formic acid)) and SCX cartridge (washing with MeOH and eluting with 2M NH_3/MeOH). Concentration *in vacuo* afforded the title compound (6 mg, 19%) as a white solid. LCMS purity >95%, ret. time 1.14 mins. HRMS (ESI +ve): $C_{24}H_{33}N_6O_2$ $[M+H]^+$: 437.2665 (Found: 437.2647). ^{1}H NMR (600 MHz, $CDCl_3$) δ 7.50 (s, 1H, H8), 7.39 – 7.34 (m, 4H, H14, H15), 6.15 – 6.11 (m, 1H, H25), 6.07 (s, 1H, H24), 4.91 (d, *J* = 6.0 Hz, 1H, H10), 4.84 – 4.67 (m, 2H, H12), 4.62 (hept, *J* = 6.8 Hz, 1H, H18), 4.36 – 4.31 (m, 2H, H26), 3.95 (t, *J* = 5.5 Hz, 2H, H28), 3.93 – 3.88 (m, 1H, H20), 3.84 (dd, *J* = 10.7, 2.6 Hz, 1H, H21), 3.65 (dd, *J* = 10.7, 7.8 Hz, 1H, H21), 2.55 – 2.50 (m, 2H, H29), 1.69 – 1.56 (m, 2H, H22), 1.55 (d, *J* = 6.8 Hz, 6H, H19), 1.04 (t, *J* = 7.5 Hz, 3H, H23). ^{13}C NMR (151 MHz, $CDCl_3$) δ

160.04 (C2), 154.82 (C6), 150.11 (C4), 139.42 (C16), 137.80 (C13), 134.61 (C8), 133.80 (C17), 127.82 (C14), 124.91 (C15), 122.40 (C25), 114.71 (C5), 68.58 (C21), 65.87 (C26), 64.47 (C28), 56.38 (C20), 46.45 (C18), 44.10 (C12), 27.20 (C29), 25.03 (C22), 22.60 (C19), 22.54 (C19), 10.95 (C23).

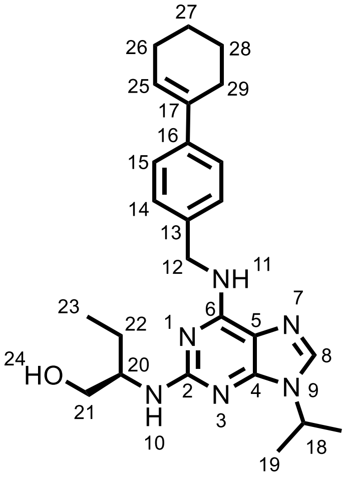

(R)-2-((9-isopropyl-6-(((2',3',4',5'-tetrahydro-[1,1'-biphenyl]-4-yl)methyl)amino)-9H-purin-2-yl)amino)butan-1-ol (22)

((R)-2-((6-((4-bromobenzyl)amino)-9-isopropyl-*9H*-purin-2-yl)amino)butan-1-ol (50 mg, 0.11 mmol) was coupled with 1-cyclohexen-1-yl-boronic acid pinacol ester (0.03 mL, 0.14 mmol) according to general procedure **4**. The residue was purified by column chromatography (0 → 15% MeOH in DCM). Concentration *in vacuo* afforded the title compound (30 mg, 60%) as a white solid. LCMS purity >95%, ret. time 1.41 mins. HRMS (ESI +ve): $C_{25}H_{35}N_6O$ $[M+H]^+$: 435.2872 (Found: 435.2869). ^{1}H NMR (600 MHz, $CDCl_3$) δ 7.53 (s, 1H, H8), 7.36 (d, *J* = 8.3 Hz, 2H, H15), 7.32 (d, *J* = 8.4 Hz, 2H, H14), 6.14 – 6.11 (m, 1H, H25), 5.89 (s, 1H, H24), 4.89 (s, 1H, H10), 4.76 (s, 2H, H12), 4.63 (hept, *J* = 6.8 Hz, 1H, H18), 3.94 – 3.89 (m, 1H, H20), 3.85 (dd, *J* = 10.7, 2.5 Hz, 1H, H21), 3.66 (dd, *J* = 10.7, 7.8 Hz, 1H, H21), 2.43 – 2.39 (m, 2H, H29), 2.24 – 2.20 (m, 2H, H26), 1.82 – 1.77 (m, 2H, H28), 1.70 – 1.65 (m, 4H, H27, H22), 1.56 (d, *J* = 6.8 Hz, 6H, H19), 1.05 (t, *J* = 7.5 Hz, 3H, H23). ^{13}C NMR (151 MHz, $CDCl_3$) δ 160.01 (C2), 155.10 (C6), 150.02 (C4), 141.68 (C16), 136.70 (C13), 136.23 (C17), 134.36 (C8), 127.65 (C14), 125.17 (C15), 124.80 (C25), 114.76 (C5), 68.78 (C21), 56.45 (C20), 46.46 (C18), 44.18 (C12), 27.39 (C29), 25.87 (C26), 25.04 (C22), 23.04 (C28), 22.61 (C19), 22.55 (C19), 22.13 (C27), 10.95 (C23).

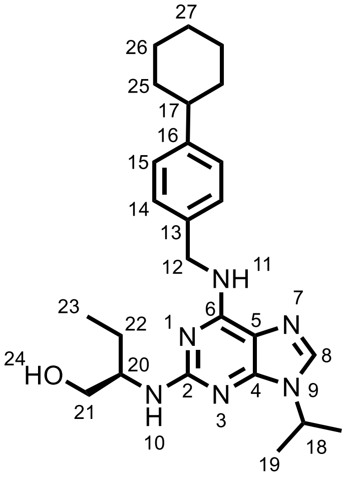

(R)-2-((6-((4-cyclohexylbenzyl)amino)-9-isopropyl-*9H*-purin-2-yl)amino)butan-1-ol (23)

To a degassed (N_2) solution of (R)-2-((9-isopropyl-6-(((2',3',4',5'-tetrahydro-[1,1'-biphenyl]-4-yl)methyl)amino)-9H-purin-2-yl)amino)butan-1-ol (30 mg, 0.07 mmol) in ethanol (1 mL, 0.07 M) and was added Palladium (7.4 mg, 0.007 mmol) (10% on carbon). The solution was purged with hydrogen 3x before leaving under a H_2 atmosphere at rt for 16 hours. The reaction was filtered through celite, and the product purified by reverse phase column (45 → 75% MeOH in water (0.1% formic acid)). Concentration *in vacuo* afforded the title compound (1.6 mg, 9%) as a white solid. LCMS purity >95%, ret. time 1.44 mins. HRMS (ESI +ve): $C_{25}H_{37}N_6O$ $[M+H]^+$: 437.3029 (Found: 437.2989). ^{1}H NMR (600 MHz, $CDCl_3$) δ 7.51 (s, 1H, H8), 7.31 (d, *J* = 8.0 Hz, 2H, H14), 7.19 (d, *J* = 8.0 Hz, 2H, H15), 5.86 (s, 1H, H24), 4.89 (d, *J* = 6.0 Hz, 1H, H10), 4.82 – 4.65 (m, 2H, H12), 4.62 (h, *J* = 6.8 Hz, 1H, H18), 3.95 – 3.88 (m, 1H, H20), 3.85 (dd, *J* = 10.7, 2.5 Hz, 1H, H21), 3.65 (dd, *J* = 10.6, 7.8 Hz, 1H, H21), 2.54 – 2.47 (m, 1H, H17), 1.91 – 1.82 (m, 4H, H25), 1.79 – 1.74 (m, 2H, H27), 1.68 – 1.56 (m, 2H, H22), 1.56 (d, *J* = 6.9, 0.9 Hz, 6H, H19), 1.45 – 1.37 (m, 4H, H26), 1.05 (t, *J* = 7.5 Hz, 3H, H23). ^{13}C NMR (151 MHz, $CDCl_3$) δ 160.08 (C2), 154.84 (C6), 150.68 (C4), 147.38 (C13), 136.00 (C6), 134.53 (C8), 127.82 (C14), 127.08 (C15), 115.82 (C5), 68.75 (C21), 56.45 (C20), 46.43 (C18), 44.29 (C17), 44.18 (C12), 34.47 (C25), 26.90 (C26), 26.15 (C27), 25.05 (C22), 22.62 (C19), 22.56 (C19), 10.96 (C23).

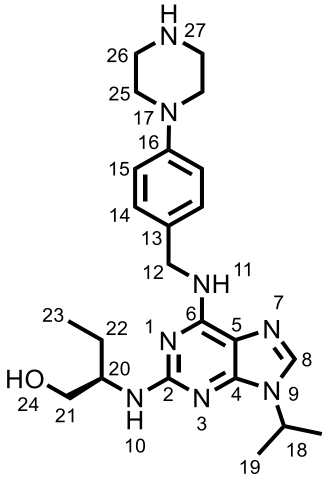

(R)-2-((9-isopropyl-6-((4-(piperazin-1-yl)benzyl)amino)-*9H*-purin-2-yl)amino)butan-1-ol (24)

To a degassed (N_2) solution of (R)-2-((6-((4-bromobenzyl)amino)-9-isopropyl-*9H*-purin-2-yl)amino)butan-1-ol (80.00 mg, 0.18 mmol), t-butyl-1-piperazinecarboxylate (44 mg, 0.24 mmol) and caesium carbonate (90 mg, 0.28 mmol) in toluene (1.5 mL, 0.1 M) was added Bis(dibenzylideneacetone)palladium (5.3 mg, 0.009 mmol) and 2-dicyclohexylphosphino-2',4',6'-triisopropylbiphenyl (8.8 mg, 0.02 mmol). The vial was sealed and heated to 110°C overnight. Conversion was incomplete so t-Butyl-1-piperazinecarboxylate (17 mg, 0.09 mmol), Bis(dibenzylideneacetone)palladium (2.7 mg, 0.005 mmol), caesium carbonate (30 mg, 0.09 mmol) and 2-dicyclohexylphosphino-2',4',6'-triisopropylbiphenyl (4.4 mg, 0.009 mmol) were added and the reaction was heated to 110°C for 18 hours. The reaction was cooled to rt, filtered through celite and purified by column chromatography (0 → 15% MeOH in DCM). Concentration *in vacuo* afford a brown solid. The solid was dissolved in DCM (1.50 mL, 0.11 M) and hydrogen chloride (4M in dioxane) (0.40 mL, 1.59 mmol) was added. The reaction was stirred at rt for 3 hours. The solution was concentrated *in vacuo* and purified by reverse phase column (20 → 45% MeOH in water (0.1%

formic acid)) Concentration *in vacuo* afforded the title compound (6.5 mg, 10%) as a white solid (formate salt). LCMS purity >95%, ret. time 0.94 mins. HRMS (ESI +ve): $C_{23}H_{35}N_8O$ $[M+H]^+$: 439.2928 (Found: 439.2895). ^1H NMR (600 MHz, $CDCl_3$) δ 7.52 (s, 1H, H8), 7.31 – 7.28 (m, 2H, H15), 6.89 (d, *J* = 8.3 Hz, 2H, H14), 6.17 (s, 1H, H24), 5.12 – 4.92 (m, 1H, H10), 4.76 – 4.65 (m, 2H, H12), 4.65 – 4.59 (m, 1H, H18), 3.95 – 3.89 (m, 1H, H20), 3.84 (dd, *J* = 10.8, 2.6 Hz, 1H, H21), 3.65 (dd, *J* = 10.7, 7.7 Hz, 1H, H21), 3.29 – 3.23 (m, 4H, H25), 3.20 – 3.14 (m, 4H, H26), 1.70 – 1.57 (m, 2H, H22), 1.55 (d, *J* = 6.8 Hz, 6H, H19), 1.04 (t, *J* = 7.4 Hz, 3H, H23). 13C NMR (151 MHz, CDCl3) δ 154.72 (C6), 150.31 (C4, C13), 134.51 (C8), 130.71 (C16), 128.89 (C15), 116.76 (C14), 114.66 (C5), 68.36 (C21), 56.32 (C20), 48.67 (C25), 46.48 (C18), 44.39 (C26), 44.02 (C12), 24.99 (C22), 22.60 (C19), 22.53 (C19), 10.95 (C23).

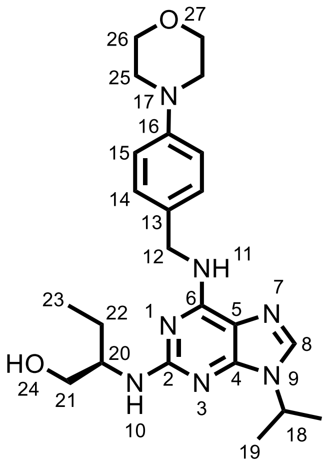

(R)-2-((9-isopropyl-6-((4-morpholinobenzyl)amino)-9H-purin-2-yl)amino)butan-1-ol (25)

To a degassed (N_2) solution of (R)-2-((6-((4-bromobenzyl)amino)-9-isopropyl-*9H*-purin-2-yl)amino)butan-1-ol (50.00 mg, 0.11 mmol), morpholine (0.013 mL, 0.15 mmol) and caesium carbonate (56 mg, 0.17 mmol) in toluene (1 mL, 0.1 M) was added Bis(dibenzylideneacetone)palladium (3.5 mg, 0.006 mmol) and 2-dicyclohexylphosphino-2',4',6'-triisopropylbiphenyl (5 mg, 0.011 mmol). The vial was sealed and heated to 110°C overnight. Conversion was incomplete so morpholine (0.013 mL, 0.15 mmol), Bis(dibenzylideneacetone)palladium (2.7 mg, 0.005 mmol), caesium carbonate (30 mg, 0.09 mmol) and 2-dicyclohexylphosphino-2',4',6'-triisopropylbiphenyl (4.4 mg, 0.009 mmol) were added and the reaction was heated to 110°C for 72 hours. The reaction was cooled to rt, filtered through celite and purified by reverse phase column (30 → 50% MeOH in water (0.1% formic acid). The product was passed through an SCX cartridge (washing with MeOH and eluting with 2M NH_3/MeOH). Concentration *in vacuo* afforded the title compound (1.5 mg, 3%) as a white solid. LCMS purity >95%, ret. time 1.05 mins. HRMS (ESI +ve $C_{23}H_{34}N_7O_2$ $[M+H]^+$: 440.2774 (Found: 440.2752). ^1H NMR (600 MHz, $CDCl_3$) δ 7.52 (s, 1H, H8), 7.30 (d, *J* = 8.4 Hz, 2H, H15), 6.90 (d, *J* = 8.3 Hz, 2H, H14), 5.81 (s, 1H, H24), 4.88 (d, *J* = 6.0 Hz, 1H, H10), 4.77 – 4.59 (m, 3H, H12, H18), 3.95 – 3.91 (m, 1H, H20), 3.89 – 3.83 (m, 5H, H21, H25), 3.66 (dd, *J* = 10.7, 7.9 Hz, 1H, H21), 3.18 – 3.14 (m, 4H, H26), 1.70 – 1.58 (m, 2H, H22), 1.56 (d, *J* = 6.8 Hz, 6H, H19), 1.06 (t, *J* = 7.5 Hz, 3H, H23). ^{13}C NMR (151 MHz, $CDCl_3$) δ 160.46 (C2), 154.81 (C6), 150.73 (C13), 150.07 (C4), 134.53 (C8), 130.05 (C16), 128.92 (C15), 115.85 (C14), 114.80 (C5), 68.84 (C21), 66.90 (C25), 56.48 (C20), 49.43 (C26), 46.43 (C18), 43.77 (C12), 25.07 (C22), 22.62 (C19), 22.56 (C19), 10.98 (C23).

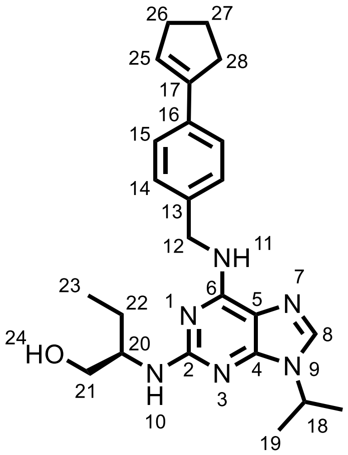

(R)-2-((6-((4-(cyclopent-1-en-1-yl)benzyl)amino)-9-isopropyl-*9H*-purin-2-yl)amino)butan-1-ol (26)

((R)-2-((6-((4-bromobenzyl)amino)-9-isopropyl-*9H*-purin-2-yl)amino)butan-1-ol (50 mg, 0.12 mmol) was coupled with 2-(1-Cyclopenten-1-yl)-4,4,5,5-tetramethyl-1,3,2-dioxaborolane (0.03 mL, 0.14 mmol) according to general procedure **4**. The residue was purified by column chromatography (0 → 10% MeOH in DCM) and concentrated *in vacuo* to afford the title compound (46 mg, 90%) as a white solid. LCMS purity >95%, ret. time 1.38 mins. HRMS (ESI +ve): $C_{24}H_{33}N_6O$ $[M+H]^+$: 421.2716 (Found: 421.2697). ^1H NMR (600 MHz, $CDCl_3$) δ 7.52 (s, 1H, H8), 7.42 (d, *J* = 8.5 Hz, 2H, H15), 7.33 (d, *J* = 8.3 Hz, 2H, H14), 6.20 – 6.18 (m, 1H, H25), 5.93 (s, 1H, H24), 4.88 (d, *J* = 5.9 Hz, 1H, H10), 4.77 (s, 2H, H12), 4.63 (hept, *J* = 6.8 Hz, 1H, H18), 3.95 – 3.88 (m, 1H, H20), 3.85 (dd, *J* = 10.7, 2.5 Hz, 1H, H21), 3.65 (dd, *J* = 10.7, 7.9 Hz, 1H, H21), 2.74 – 2.69 (m, 2H, H28), 2.57 – 2.52 (m, 2H, H26), 2.07 – 2.00 (m, 2H, H27), 1.69 – 1.57 (m, 2H, H22), 1.56 (d, *J* = 6.7, 1.1 Hz, 6H, H19), 1.05 (t, *J* = 7.5 Hz, 3H, H23). ^{13}C NMR (151 MHz, $CDCl_3$) δ 160.10 (C2), 154.87 (C6), 150.15 (C4) 142.08 (C16), 137.15 (C17), 136.04 (C13), 134.58 (C8), 127.71 (C14), 126.15 (C25), 125.79 (C15), 114.75 (C5), 68.73 (C21), 56.44 (C20), 46.44 (C18), 44.07 (C12), 33.35 (C26), 33.21 (C28), 25.06 (C22), 23.33 (C27), 22.61 (C19), 22.55 (C19), 10.96 (C23).

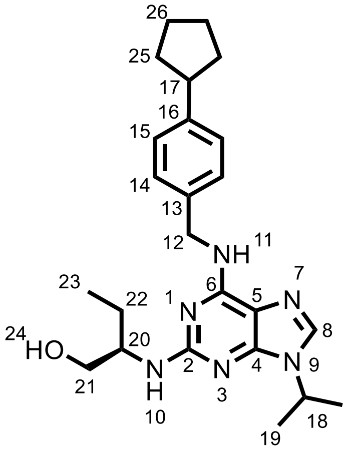

(R)-2-((6-((4-cyclopentylbenzyl)amino)-9-isopropyl-*9H*-purin-2-yl)amino)butan-1-ol (27)

To a degassed (N_2) solution of (R)-2-((6-((4-(cyclopent-1-en-1-yl)benzyl)amino)-9-isopropyl-*9H*-purin-2-yl)amino)butan-1-ol (30 mg, 0.07 mmol) in methanol (0.50 mL, 0.07 M) and ethyl acetate (0.50 mL, 0.07 M) was added Palladium (7.6 mg, 0.007 mmol) (10% on carbon). The solution was purged with hydrogen 3x before leaving under a H_2 atmosphere at rt for 16 hours. The reaction was filtered through celite and the product purified by column chromatography (0 → 15% MeOH in DCM). The product was further purified by reverse phase column (30 → 100% MeOH in water (0.1% formic acid)) and SCX cartridge (washing with MeOH and eluting with 2M NH_3/MeOH). Concentration *in vacuo* afforded the title compound (3 mg, 10%) as a yellow solid. LCMS purity >95%, ret. time 1.40 mins. HRMS (ESI +ve): $C_{24}H_{35}N_6O$ $[M+H]^+$: 423.2872 (Found: 423.2855). ^{1}H NMR (600 MHz, $CDCl_3$) δ 7.50 (s, 1H, H8), 7.30 (d, *J* = 8.1 Hz, 2H, H14), 7.22 (d, *J* = 8.1 Hz, 2H, H15), 5.94 (s, 1H, H24), 4.93 – 4.84 (m, 1H, H10), 4.75 (s, 2H, H12), 4.62 (hept, *J* = 6.8 Hz, 1H, H18), 3.95 – 3.87 (m, 1H, H20), 3.85 (dd, *J* = 10.7, 2.5 Hz, 1H, H21), 3.65 (dd, *J* = 10.7, 7.8 Hz, 1H, H21), 3.00 (tt, *J* = 9.8, 7.5 Hz, 1H, H17), 2.10 – 2.04 (m, 2H, H25), 1.87 – 1.79 (m, 2H, H26), 1.74 – 1.67 (m, 2H, H26), 1.67 – 1.57 (m, 2H, H22), 1.55 (dd, *J* = 6.8, 1.0 Hz, 6H, H19), 1.05 (t, *J* = 7.5 Hz, 3H, H23). ^{13}C NMR (151 MHz, $CDCl_3$) δ 160.09 (C2), 154.85 (C6), 150.93 (C4), 145.77 (C16), 135.96 (C13), 134.54 (C8), 127.76 (C14), 127.35 (C15), 114.75 (C5), 68.71 (C21), 56.43 (C20), 46.42 (C18), 45.65 (C17), 44.19 (C12), 34.64 (C25), 25.50 (C26), 25.05 (C22), 22.61 (C19), 22.55 (C19), 10.96 (C23).

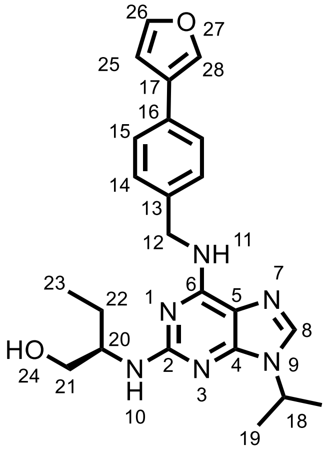

(R)-2-((6-((4-(furan-3-yl)benzyl)amino)-9-isopropyl-*9H*-purin-2-yl)amino)butan-1-ol (28)

(R)-2-((6-((4-bromobenzyl)amino)-9-isopropyl-*9H*-purin-2-yl)amino)butan-1-ol (40 mg, 0.09 mmol) was coupled with 3-Furanylboronic acid (12 mg, 0.11 mmol) according to general procedure **4**. The residue was purified by column chromatography (0 → 10% MeOH in DCM). The compound was further purified by reverse phase column (40 → 60% MeOH in water (0.1% formic acid)) and passed through an SCX cartridge (washing with MeOH and eluting with 2M NH_3/MeOH). Concentration *in vacuo* afforded the title compound (12 mg, 29%) as a white solid. LCMS purity >95%, ret. time 1.22 mins. HRMS (ESI +ve $C_{23}H_{29}N_6O_2$ $[M+H]^+$: 421.2352 (Found: 421.2336). ^{1}H NMR (600 MHz, $CDCl_3$) δ 7.73 (s, 1H, H28), 7.49 – 7.44 (m, 4H, H8, H15, H26), 7.40 – 7.37 (m, 2H, H14), 6.70 (dd, *J* = 1.9, 0.9 Hz, 1H, H25), 6.21 (s, 1H, H24), 4.93 (d, *J* = 6.0 Hz, 1H, H10), 4.85 – 4.68 (m, 2H, H12), 4.61 (p, *J* = 6.8 Hz, 1H, H18), 3.95 – 3.88 (m, 1H, H20), 3.84 (dd, *J* = 10.7, 2.6 Hz, 1H, H21), 3.65 (dd, *J* = 10.7, 7.7 Hz, 1H, H21), 1.69 – 1.56 (m, 2H, H22), 1.54 (dd, *J* = 6.8, 1.0 Hz, 6H, H19), 1.04 (t, *J* = 7.4 Hz, 3H, H23). ^{13}C NMR (151 MHz, $CDCl_3$) δ 160.06 (C2), 154.87 (C6), 150.18 (C4), 143.67 (C26), 138.47 (C28), 137.57 (C13), 134.60 (C8), 131.51 (C6), 128.20 (C14), 126.14 (C17), 126.04 (C15), 114.71 (C5), 108.84 (C25), 68.52 (C21), 56.36 (C20), 46.44 (C18), 44.10 (C12), 25.03 (C22), 22.59 (C19), 22.53 (C19), 10.95 (C23).

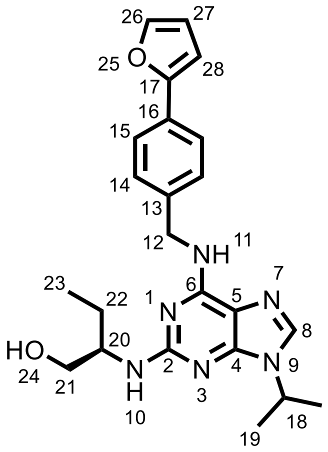

(R)-2-((6-((4-(furan-2-yl)benzyl)amino)-9-isopropyl-*9H*-purin-2-yl)amino)butan-1-ol (29)

(2R)-2-[[9-isopropyl-6-[[4-(4,4,5,5-tetramethyl-1,3,2-dioxaborolan-2-yl)phenyl]methylamino]purin-2-yl]amino]butan-1-ol (35 mg, 0.0729 mmol)) was coupled with 2-Bromofuran (0.01 mL, 0.087 mmol) according to general procedure **4**. The residue was purified by reverse phase column (50 → 70% MeOH in water (0.1% formic acid)) and passed through an SCX cartridge (washing with MeOH and eluting with 2M NH_3/MeOH). Concentration *in vacuo* afforded the title compound (5.5 mg, 17%) as a white solid. LCMS purity >95%, ret. time 1.26 mins. HRMS (ESI +ve $C_{23}H_{29}N_6O_2$ $[M+H]^+$: 421.2346 (Found: 421.2349). ^{1}H NMR (600 MHz, $CDCl_3$) δ 7.65 (d, *J* = 8.3 Hz, 2H, H15), 7.49 (s, 1H, H8), 7.48 (d, *J* = 1.1 Hz, 1H, H26), 7.39 (d, *J* = 8.3 Hz, 2H, H14), 6.65 (dd, *J* = 3.4, 0.8 Hz, 1H, H28), 6.48 (dd, *J* = 3.4, 1.8 Hz, 1H, H27), 6.10 (s, 1H, H24), 4.92 (d, *J* = 6.1 Hz, 1H, H10), 4.80 (s, 2H, H12), 4.61 (h, *J* = 6.8 Hz, 1H, H18), 3.94 – 3.89 (m, 1H, H20), 3.84 (dd, *J* = 10.7, 2.6 Hz, 1H, H21), 3.65 (dd, *J* = 10.7, 7.8 Hz, 1H, H21), 1.69 – 1.57 (m, 2H, H22), 1.55 (dd, *J* = 6.8, 1.0 Hz, 6H, H19), 1.04 (t, *J* = 7.5 Hz, 3H, H23). ^{13}C NMR (151 MHz, $CDCl_3$) δ 160.04 (C2), 154.85 (C6), 153.78 (C17), 150.10 (C4), 142.03 (C26), 137.86 (C13), 134.64 (C8), 130.05, (C16), 128.07 (C14), 124.01 (C15), 114.74 (C5), 111.64 (C27), 104.95 (C28), 68.58 (C21), 56.40 (C20), 46.46 (C18), 44.18 (C12), 25.03 (C22), 22.60 (C19), 22.54 (C19), 10.95 (C23).

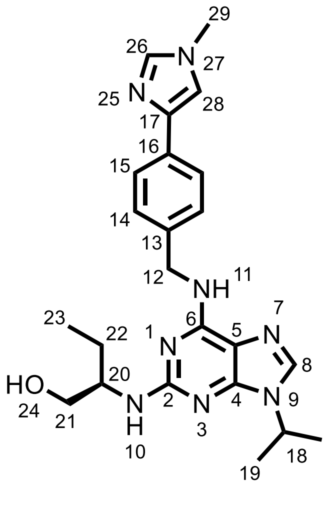

(R)-2-((9-isopropyl-6-((4-(1-methyl-1H-imidazol-4-yl)benzyl)amino)-9H-purin-2-yl)amino)butan-1-ol (30)

(R)-2-((9-isopropyl-6-((4-(4,4,5,5-tetramethyl-1,3,2-dioxaborolan-2-yl)benzyl)amino)-*9H*-purin-2-yl)amino)butan-1-ol (40 mg, 0.083 mmol) was coupled with 4-Bromo-1-methyl-1H-imidazole (0.01 mL, 0.20 mmol) according to general procedure **4**. The residue was purified by reverse phase column (20 → 40% MeOH in water (0.1% formic acid)) and passed through an SCX cartridge (washing with MeOH and eluting with 2M NH_3/MeOH). Concentration *in vacuo* afforded the title compound (5.5 mg, 14%) as a white solid. LCMS purity >95%, ret. time 1.25 mins. HRMS (ESI +ve): $C_{23}H_{31}N_8O$ $[M+H]^+$: 435.2621 (Found: 435.2065). ^1H NMR (600 MHz, $CDCl_3$) δ 7.73 (d, *J* = 8.2 Hz, 2H, H15), 7.50 (s, 1H, H8), 7.48 (s, 1H, H26), 7.38 (d, *J* = 8.2 Hz, 2H, H14), 7.17 (s, 1H, H28), 4.93 (d, *J* = 6.0 Hz, 1H, H10), 4.78 (s, 2H, H12), 4.62 (h, *J* = 6.8 Hz, 1H, H18), 3.97 – 3.86 (m, 1H, H20), 3.84 (dd, *J* = 10.7, 2.6 Hz, 1H, H21), 3.74 (s, 3H, H29), 3.68 – 3.63 (dd, *J* = 7.7 Hz, 1H, H21), 1.68 – 1.57 (m, 2H, H22), 1.56 (d, *J* = 6.9 Hz, 6H, H19), 1.04 (t, *J* = 7.5 Hz, 3H, H23).

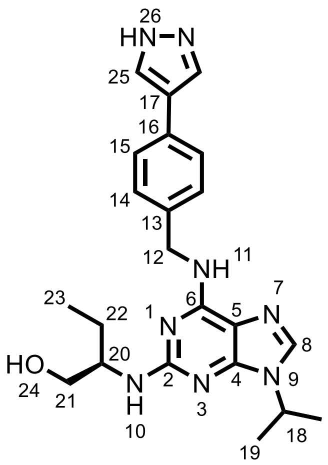

(R)-2-((6-((4-(1H-pyrazol-4-yl)benzyl)amino)-9-isopropyl-9H-purin-2-yl)amino)butan-1-ol (31)

(R)-2-((6-((4-bromobenzyl)amino)-9-isopropyl-*9H*-purin-2-yl)amino)butan-1-ol (40 mg, 0.09 mmol) was coupled with 1-Boc-pyrazole-4-boronic acid pinacol ester (40 mg, 0.14 mmol) according to general procedure **4**. The residue was purified by column chromatography (0 → 20% MeOH in DCM). The de-boc compound was further purified by reverse phase column (40 → 55% MeOH in water (0.1% formic acid)) and passed through an SCX cartridge (washing with MeOH and eluting with 2M NH_3/MeOH). Concentration *in vacuo* afforded the title compound (4.8 mg, 9%) as a white solid. LCMS purity >95%, ret. time 1.03 mins. HRMS (ESI +ve) $C_{22}H_{29}N_8O$ $[M+H]^+$: 421.2464 (Found: 421.2457). ^1H NMR (600 MHz, DMSO- d_6) δ 12.88 (s, 1H, H26), 8.00 (s, 2H, H25), 7.78 (s, 1H, H8), 7.52 (d, *J* = 8.2 Hz, 2H, H15), 7.34 (d, *J* = 8.0 Hz, 2H, H14), 5.84 (s, 1H, H24), 4.69 – 4.47 (m, 3H, H12, H18), 3.85 – 3.77 (m, 1H, H20), 3.50 – 3.44 (m, 1H, H21), 3.40 – 3.35 (m, 1H, H21), 1.61 (ddd, *J* = 13.4, 7.7, 5.9 Hz, 1H, H22), 1.49 – 1.41 (m, 7H, H22, H19), 0.85 (t, *J* = 7.4 Hz, 3H, H23). ^{13}C NMR (151 MHz, DMSO-d_6) δ 159.48 (C2), 154.91 (C6), 151.12 (C4), 138.77 (C17), 135.45 (C8), 131.65 (C16), 128.37 (C14), 125.30 (C15, C25), 121.54 (C13), 114.20 (C4), 63.54 (C21), 54.55 (C20), 46.08 (C18), 42.99 (C12), 24.36 (C22), 22.58 (C19), 22.49 (C19), 11.17 (C23).

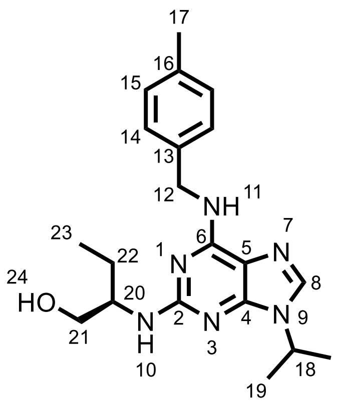

(R)-2-((9-isopropyl-6-((4-methylbenzyl)amino)-*9H*-purin-2-yl)amino)butan-1-ol (32)

The product was synthesised following general procedure **3**, using 2-chloro-9-isopropyl-N-(4-methylbenzyl)-*9H*-purin-6-amine (100 mg, 0.32 mmol) (R)-(-)-2-amino-1-butanol (0.18 mL, 1.8999 mmol). The residue was purified by column chromatography (60 → 100% EtOAc in cyclohexane) to afford the title compound (587 mg, 95%) as a white solid. LCMS purity >95%, ret. time 1.33 mins. HRMS (ESI +ve) $C_{20}H_{29}N_6O$ $[M+H]^+$: 369.2397 (Found: 369.2382). ^{1}H NMR (600 MHz, DMSO-d$_6$) δ 7.77 (s, 1H, H8), 7.24 (d, *J* = 7.8 Hz, 2H, H15), 7.09 (d, *J* = 7.8 Hz, 2H, H14), 5.86 – 5.76 (m, 1H, H24), 4.58 – 4.45 (m, 3H, H12, H18), 3.84 – 3.76 (m, 1H, H20), 3.49 – 3.42 (m, 1H, H21), 3.39 – 3.34 (m, 1H, H21), 2.25 (s, 3H, H17), 1.65 – 1.38 (m, 2H, H22), 1.46 (dd, *J* = 6.8, 4.9 Hz, 6H, H19), 0.85 (t, *J* = 7.4 Hz, 3H, H23). ^{13}C NMR (151 MHz, DMSO-d$_6$) δ 159.46 (C2), 154.80 (C6), 151.18 (C1), 138.19 (C16), 135.85 (C13), 135.41 (C8), 129.05 (C14), 127.84 (C15), 114.11 (C15), 63.54 (C21), 54.52 (C20), 46.06 (C18), 42.80 (C12), 24.35 (C22), 22.49 (C19), 21.14 (C19), 11.15 (C23).

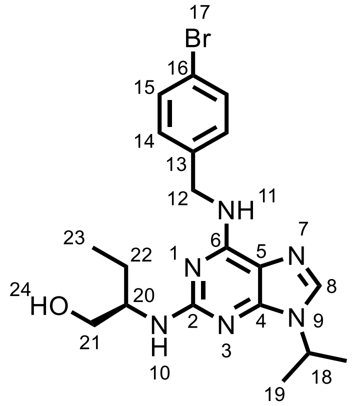

(R)-2-((6-((4-bromobenzyl)amino)-9-isopropyl-*9H*-purin-2-yl)amino)butan-1-ol (33)

The product was synthesised following general procedure **3**, using N-(4-bromobenzyl)-2-chloro-9-isopropyl-*9H*-purin-6-amine (540 mg, 1.42 mmol) and (R)-2-amino-1-butanol (1.1 mL, 11.35 mmol). The residue was purified by column chromatography (60 → 100% EtOAc in cyclohexane) to afford the title compound (587 mg, 95%) as an orange oil that solidified upon standing. HRMS (ESI +ve): $C_{19}H_{26}BrN_6O$ $[M+H]^+$: 433.151 (Found: 433.1348). ^{1}H NMR (600 MHz, $CDCl_3$) δ 7.51 (s, 1H, H8), 7.46 (d, *J* = 8.4 Hz, 2H, H15), 7.26 (d, *J* = 8.3 Hz, 2H, H14), 6.02 (s, 1H, H24), 4.88 (d, *J* = 6.1 Hz, 1H, H10), 4.81 – 4.67 (m, 2H, H12), 4.66 – 4.58 (m, 1H, H18), 3.92 – 3.87 (m, 1H, H20), 3.84 (dd, *J* = 10.8, 2.6 Hz, 1H, H21), 3.65 (dd, *J* = 10.7, 7.8 Hz, 1H, H21), 1.69 – 1.57 (m, 2H, H22), 1.55 (d, *J* = 6.8, 1.2 Hz, 6H,

H19), 1.04 (t, *J* = 7.4 Hz, 3H, H23). ^{13}C NMR (151 MHz, CDCl3) δ 160.03 (C2), 154.77 (C6), 150.03 (C4), 137.95 (C16), 134.73 (C8), 131.65 (C15), 129.35 (C14), 121.14 (C13), 114.66 (C5), 68.53 (C21), 56.37 (C20), 46.48 (C18), 43.53 (C12), 25.02 (C22), 22.60 (C19), 10.94 (C23).

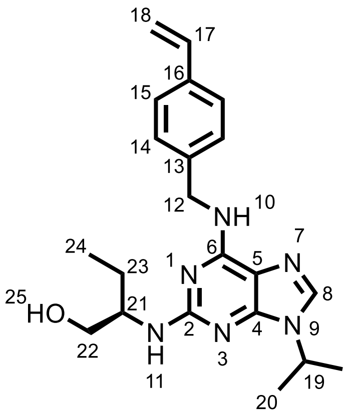

(R)-2-((9-isopropyl-6-((4-vinylbenzyl)amino)-9H-purin-2-yl)amino)butan-1-ol (34)

The product was synthesised following general procedure **3**, using 2-chloro-9-isopropyl-N-[(4-vinylphenyl)methyl]purin-6-amine (500 mg, 1.52 mmol) and (R)-2-amino-1-butanol (0.86 mL, 9.15 mmol). The residue was purified using column chromatography (70 → 100% EtOAc in cyclohexane) and concentration in vacuo afforded the title compound (426 mg, 73%) as a white solid. LCMS purity >95%, ret. time 1.36 mins. HRMS (ESI +ve $C_{21}H_{29}N_6O$ $[M+H]^+$: 381.2397 (Found: 381.2387). ^{1}H NMR (600 MHz, $CDCl_3$) δ 7.50 (s, 1H, H8), 7.38) (s, 2H, H15), 7.34 (d, *J* = 7.8 Hz, 2H, H14), H14, 6.72 (dd, *J* = 17.6, 10.8 Hz, 1H, H17), 5.75 (d, *J* = 17.6 Hz, 1H, H18), 5.25 (d, *J* = 10.8 Hz, 1H, H19), 4.89 (d, *J* = 6.0 Hz, 1H, H10), 4.84 – 4.68 (m, 2H, H12), 4.62 (p, *J* = 6.8 Hz, 1H, H18), 3.94 – 3.87 (m, 1H, H21), 3.84 (d, *J* = 10.0 Hz, 1H, H22), 3.65 (dd, *J* = 10.6, 7.9 Hz, 1H, H22), 1.69 – 1.57 (m, 2H, H23), 1.55 (d, *J* = 6.9 Hz, 6H, H20), 1.04 (t, *J* = 7.5 Hz, 3H, H24). ^{13}C NMR (151 MHz, $CDCl_3$) δ 160.08 (C2), 154.86 (C6), 150.08 (C4), 138.36 (C13), 136.75 (C16), 136.46 (C17), 134.60 (C8), 127.90 (C14), 126.43 (C8), 114.73 (C5), 113.78 (C18), 68.65 (C22), 56.41 (C21), 46.44 (C19), 44.20 (C12), 25.04 (C23), 22.61 (C20), 22.54 (C20), 10.95 (C24).

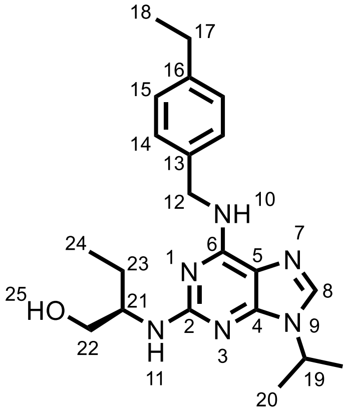

(R)-2-((6-((4-ethylbenzyl)amino)-9-isopropyl-*9H*-purin-2-yl)amino)butan-1-ol (35)

To a degassed (N_2) solution of (R)-2-((9-isopropyl-6-((4-vinylbenzyl)amino)-9H-purin-2-yl)amino)butan-1-ol (40 mg, 0.10 mmol) in ethanol (1 mL, 0.1 M) was added Palladium (11 mg, 0.01 mmol) (10% on carbon). The solution was purged with hydrogen 3x before leaving under a H_2 atmosphere at rt for 16 hours. The reaction was filtered through celite and the product purified by column chromatography (0 → 15% MeOH in DCM). Concentration *in vacuo* afforded the title compound (32 mg, 79%) as a colourless oil that solidified upon standing. LCMS purity >95%, ret. time 1.41 mins. HRMS (ESI +ve): $C_{21}H_{31}N_6O$ $[M+H]^+$: 383.2553 (Found: 383.2541). ^1H NMR (600 MHz, $CDCl_3$) δ 7.48 (s, 1H, H8), 7.30 (d, *J* = 7.7 Hz, 2H, H14), 7.18 (d, *J* = 7.7 Hz, 2H, H15), 6.04 (s, 1H, H24), 4.90 (d, *J* = 6.1 Hz, 1H, H10), 4.83 – 4.66 (m, 2H, H12), 4.61 (hept, *J* = 6.9 Hz, 1H, H19), 3.95 – 3.88 (m, 1H, H21), 3.84 (dd, *J* = 10.9, 2.5 Hz, 1H, H22), 3.65 (dd, *J* = 10.7, 7.8 Hz, 1H, H22), 2.65 (q, *J* = 7.6 Hz, 2H, H17), 1.63 (ddt, *J* = 30.3, 14.7, 7.1 Hz, 2H, H23), 1.54 (d, *J* = 6.9 Hz, 6H, H20), 1.26 (ddd, *J* = 22.9, 7.9, 6.8 Hz, 3H, H18), 1.04 (t, *J* = 7.5 Hz, 3H, H24). ^{13}C NMR (151 MHz, $CDCl_3$) δ 160.10 (C2), 154.87 (C6), 150.20 (C4), 143.45 (C16), 135.92 (C13), 134.51 (C8), 128.10 (C15), 127.81 (C13), 114.71 (C5), 68.64 (C22), 56.40 (C21), 46.41 (C19), 44.18 (C12), 28.54 (C17), 25.04 (C23), 22.60 (C20), 22.54 (C20), 15.66 (C18), 10.95 (C24).

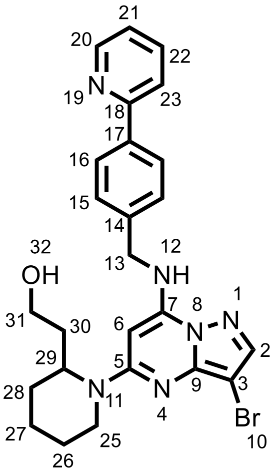

2-(1-(3-bromo-7-((4-(pyridin-2-yl)benzyl)amino)pyrazolo[1,5-a]pyrimidin-5-yl)piperidin-2-yl)ethan-1-ol (36)

3-Bromo-5,7-dichloropyrazolo[1,5-a]pyrimidine (36 mg, 0.13 mmol) was coupled (4-(pyridin-2-yl)phenyl)methanamine (25 mg, 0.13 mmol) according to general procedure **5**. The residue was purified by column chromatography (0 → 20% MeOH in DCM) and concentration *in vacuo* afforded the 3-bromo-5-chloro-N-(4-(pyridin-2-yl)benzyl)pyrazolo[1,5-a]pyrimidin-7-amine (40 mg, 71%) as a yellow solid. 3-bromo-5-chloro-N-(4-(pyridin-2-yl)benzyl)pyrazolo[1,5-a]pyrimidin-7-amine (40 mg, 0.097 mmol) was coupled with 2-piperidineethanol (0.04 mL, 0.28 mmol) according to general procedure **8**. The reside was purified by column chromatography (0 → 10% MeOH In DCM) followed by further purification by reverse phase column (50 – 80% MeOH in water (0.1% formic acid)). The product was passed through an SCX cartridge (washing with MeOH and eluting with 2M NH_3/MeOH). A second purification was performed using preparative HPLC (40 → 100% MeOH in water (0.1% formic acid)) and the product was passed through an SCX cartridge (washing with MeOH and eluting with 2M NH_3/MeOH). Concentration *in vacuo* afforded the title compound (4 mg, 3%) as a yellow solid. LCMS purity >95%, ret. time 1.41 mins. HRMS (ESI +ve) $C_{25}H_{28}BrN_6O$ $[M+H]^+$: 507.1508 (Found:

507.0924). ^{1}H NMR (600 MHz, $CDCl_3$) δ 8.72 (d, *J* = 5.2 Hz, 1H, H20), 8.04 (dd, *J* = 8.4, 1.8 Hz, 2H, H16), 7.79 (td, *J* = 7.6, 1.8 Hz, 1H, H22), 7.75 (d, *J* = 9.2 Hz, 2H, H2, H23), 7.51 – 7.48 (m, 2H, H15), 7.29 – 7.27 (m, 1H, H21), 6.47 (t, *J* = 5.8 Hz, 1H, H12), 5.33 (s, 1H, H6), 5.19 – 5.13 (m, 1H, H29), 4.62 (d, *J* = 5.8 Hz, 2H, H13), 3.69 (d, *J* = 14.3 Hz, 1H, H31), 3.60 (d, *J* = 11.9 Hz, 1H, H25), 3.33 (td, *J* = 11.8, 2.3 Hz, 1H, H25), 3.04 (td, *J* = 13.4, 2.9 Hz, 1H, H31), 2.09 – 2.03 (m, 1H, H26), 1.84 – 1.77 (m, 1H, H28), 1.76 – 1.63 (m, 5H, H26, H27, H28, H30), 1.55 – 1.49 (m, 1H, H30). ^{13}C NMR (151 MHz, $CDCl_3$) δ 158.18 (C5), 156.76 (C18), 149.78 (C20), 147.08 (C7), 145.18 (C9), 143.16 (C2), 139.33 (C17), 137.01 (C14), 136.90 (C22), 127.64 (C15), 127.56 (C16), 122.39 (C22), 120.57 (C24), 78.18 (C3), 71.25 (C6), 58.01 (C25), 47.19 (C29), 46.00 (C13), 41.02 (C31), 32.49 (C26), 29.66 (C28), 25.64 (C30), 19.54 (C27).

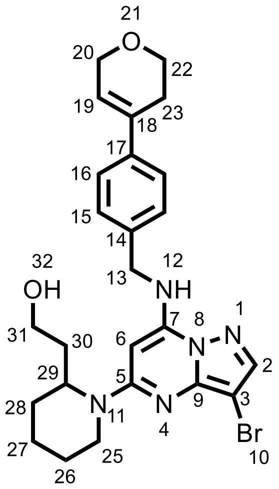

2-(1-(3-bromo-7-((4-(3,6-dihydro-2H-pyran-4-yl)benzyl)amino)pyrazolo[1,5-a]pyrimidin-5-yl)piperidin-2-yl)ethan-1-ol (37)

3-Bromo-5,7-dichloropyrazolo[1,5-a]pyrimidine (100 mg, 0.37 mmol) was coupled with (4-(3,6-dihydro-2H-pyran-4-yl)phenyl)methanamine (70 mg, 0.37 mmol) according to general procedure **5**. The residue was purified by column chromatography (0 → 10% MeOH in DCM) and concentration *in vacuo* afforded 3-bromo-5-chloro-N-(4-(3,6-dihydro-2H-pyran-4-yl)benzyl)pyrazolo[1,5-a]pyrimidin-7-amine (115 mg, 73%) as a yellow solid. 3-bromo-5-chloro-N-(4-(pyridin-2-yl)benzyl)pyrazolo[1,5-a]pyrimidin-7-amine (40 mg, 0.097 mmol) was coupled with 2-piperidineethanol (0.04 mL, 0.28 mmol) according to general procedure **8**. The reside was purified by column chromatography (0 → 10% MeOH In DCM) followed by further purification by reverse phase column (50 – 80% MeOH in water (0.1% formic acid)). The product was passed through an SCX cartridge (washing with MeOH and eluting with 2M NH_3/MeOH). A second purification was performed using preparative HPLC (40 → 100% MeOH in water (0.1% formic acid)) and the product was passed through an SCX cartridge (washing with MeOH and eluting with 2M NH_3/MeOH). Concentration *in vacuo* afforded the title compound (3.5 mg, 7%) as a yellow solid. LCMS purity >95%, ret. time 1.41 mins. HRMS (ESI +ve) $C_{25}H_{31}BrN_5O_2$ $[M+H]^+$: 512.1661 (Found: 512.1652). ^{1}H NMR (600 MHz, $CDCl_3$) δ 7.74 (s, 1H, H2), 7.45 – 7.42 (m, 2H, H15), 7.36 (d, *J* = 8.3 Hz, 2H, H16), 6.38 (t, *J* = 5.7 Hz, 1H, H12), 6.19 – 6.15 (m, 1H, H19), 5.32 (s, 1H, H6), 5.21 – 5.14 (m, 1H, H29), 4.53 (d, *J* = 5.7 Hz, 2H, H13), 4.35 (q, *J* = 2.8 Hz, 2H, H20), 3.96 (t, *J* = 5.5 Hz, 2H, H22), 3.73 – 3.66 (m, 1H, H26), 3.65 – 3.59 (m, 1H, H31), 3.34 (td, *J* = 11.9, 2.1 Hz, 1H, H25), 3.05 (td, *J* = 13.4, 2.9 Hz, 1H, H31), 2.56 – 2.52 (m, 2H, H23), 2.11 – 2.05 (m, 1H, H26), 1.85 – 1.62 (m, 5H, H26, H27, H28, H30). ^{13}C NMR (151 MHz, $CDCl_3$) δ 158.21 (C5), 147.05 (C7), 145.16 (C9), 143.13 (C2), 140.21 (C17), 135.15 (C14), 133.55 (C18), 127.54 (C15), 125.29 (C16),

123.01 (C19), 78.16 (C3), 71.10 (C6), 65.85 (C20), 64.42 (C22), 57.99 (C25), 47.16 (C29), 45.99 (C13), 41.05 (C31), 32.48 (C26), 29.67 (C28), 27.16 (C23), 25.66 (C30), 19.56 (C27).

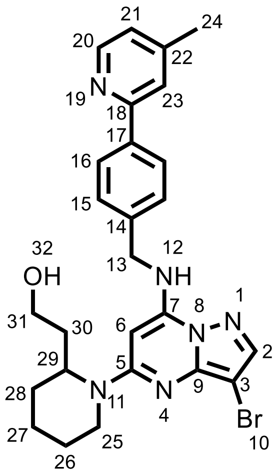

2-(1-(3-bromo-7-((4-(4-methylpyridin-2-yl)benzyl)amino)pyrazolo[1,5-*a*]pyrimidin-5-yl)piperidin-2-yl)ethan-1-ol (38)

The product was synthesised from 3-Bromo-5,7-dichloropyrazolo[1,5-*a*]pyrimidine (80 mg, 0.30 mmol) and [4-(4-methyl-2-pyridyl)phenyl]methanamine (59 mg, 0.30 mmol) according to general procedure **5**. This afforded 3-bromo-5-chloro-N-(4-(4-methylpyridin-2-yl)benzyl)pyrazolo[1,5-a]pyrimidin-7-amine (63 mg, 49%) as a yellow solid. 3-bromo-5-chloro-*N*-(4-(4-methylpyridin-2-yl)benzyl)pyrazolo[1,5-*a*]pyrimidin-7-amine (60 mg, 0.14 mmol) was coupled with 2-piperidineethanol (0.04 mL, 0.28 mmol) according to general procedure **8**. The reside was purified by column chromatography (0 → 10% MeOH In DCM) followed by further purification by reverse phase column (40 – 70% MeOH in water (0.1% formic acid)). The product was passed through an SCX cartridge (washing with MeOH and eluting with 2M NH3/MeOH) and concentrated *in vacuo* to afford the title compound (11 mg, 15%) as an orange solid. LCMS purity >95%, ret. time 1.30 mins. HRMS (ESI +ve $C_{26}H_{30}BrN_6O$ $[M+H]^+$: 521.1664 (Found: 521.1657). ^1H NMR (600 MHz, $CDCl_3$) δ 8.57 (d, *J* = 5.0 Hz, 1H, H20), 8.01 (d, *J* = 8.3 Hz, 2H, H16), 7.76 (s, 1H, H2), 7.56 (s, 1H, H23), 7.48 (d, *J* = 8.1 Hz, 2H, H15), 7.10 (d, *J* = 4.1 Hz, 1H, H21), 6.48 (t, *J* = 5.8 Hz, 1H, H12), 5.32 (s, 1H, H6), 5.15 (s, 1H, H29), 4.61 (d, *J* = 5.8 Hz, 2H, H13), 3.75 – 3.65 (m, 1H, H31), 3.64 – 3.57 (m, 1H, H25), 3.33 (td, *J* = 11.8, 2.3 Hz, 1H, H25), 3.03 (td, *J* = 13.4, 2.9 Hz, 1H, H31), 2.45 (s, 3H, H24), 2.06 (ddt, *J* = 14.4, 11.9, 2.5 Hz, 1H, H26), 1.83 – 1.77 (m, 1H, H28), 1.76 – 1.62 (m, 5H, H26, H27, H28, H30), 1.56 – 1.48 (m, 1H, H30). ^{13}C NMR (151 MHz, $CDCl_3$) δ 158.18 (C5), 156.64, (C18), 149.48 (C20), 148.00 (C22), 147.09 (C7), 145.18 (C9), 143.14 (C2), 139.43 (C17), 136.86 (C14), 127.58 (C15, C16), 123.42 (C21), 121.57 (C23), 78.16 (C3), 71.27 (C6), 58.01 (C25), 47.21 (C29), 46.01 (C13), 41.01 (C31), 32.47 (C26), 29.65 (C28), 25.63 (C30), 21.27 (C24), 19.53 (C27).

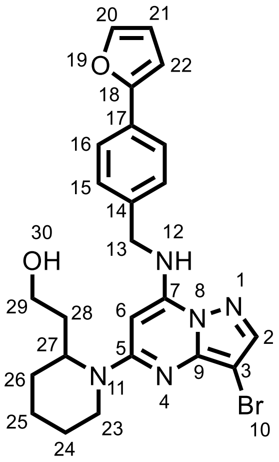

2-[1-[3-bromo-7-[[4-(2-furyl)phenyl]methylamino]pyrazolo[1,5-a]pyrimidin-5-yl]-2-piperidyl]ethanol (39)

The product was synthesised from 3-Bromo-5,7-dichloropyrazolo[1,5-a]pyrimidine (100 mg, 0.37 mmol) and [4-(2-furyl)phenyl]methanamine(79 mg, 0.37 mmol) according to general procedure **5**. This afforded 3-bromo-5-chloro-N-[[4-(2-furyl)phenyl]methyl]pyrazolo[1,5-a]pyrimidin-7-amine (105 mg, 80%) as a yellow solid. 3-bromo-5-chloro-N-[[4-(2-furyl)phenyl]methyl]pyrazolo[1,5-a]pyrimidin-7-amine (105 mg, 0.26 mmol) was coupled with 2-piperidineethanol (0.10 mL, 0.78 mmol) according to general procedure **8**. The reside was purified by reverse phase column (50 – 80% MeOH in water (0.1% formic acid)). The product was passed through an SCX cartridge (washing with MeOH and eluting with 2M NH_3/MeOH). Concentration *in vacuo* afforded the title compound (28 mg, 22%) as an orange solid. LCMS purity >95%, ret. time 1.68 mins. HRMS (ESI +ve) $C_{25}H_{27}BrN_5O_2$ $[M+H]^+$: 496.1348 (Found: 496.0665). ^1H NMR (600 MHz, $CDCl_3$) δ 7.75 (s, 1H, H2), 7.70 (d, J = 8.1 Hz, 2H, H16), 7.50 (d, J = 1.7 Hz, 1H, H20), 7.39 (d, J = 8.1 Hz, 2H, H15), 6.69 (d, J = 3.4 Hz, 1H, H22), 6.51 (dd, J = 3.4, 1.8 Hz, 1H, H21), 6.45 (t, J = 5.8 Hz, 1H, H12), 5.32 (s, 1H, H6), 5.17 (d, J = 11.6 Hz, 1H, H27), 4.55 (d, J = 5.7 Hz, 2H, H13), 3.68 (d, J = 13.9 Hz, 1H, H29), 3.64 – 3.58 (m, 1H, H23), 3.34 (td, J = 11.9, 2.1 Hz, 1H, H23), 3.04 (td, J = 13.4, 2.9 Hz, 1H, H29), 2.10 – 2.04 (m, 1H, H24), 1.84 – 1.77 (m, 1H H26), 1.77 – 1.62 (m, 5H, H24, H25, H26, H28), 1.58 – 1.46 (m, 1H, H28). ^{13}C NMR (151 MHz, $CDCl_3$) δ 158.19 (C5), 153.36 (C18), 147.06 (C7), 145.16 (C9), 143.13 (C2), 142.32 (C20), 135.15 (C14), 130.79 (C17), 127.70 (C15), 124.33 (C16), 111.77 (C21), 105.44 (C22), 78.16 (C3), 71.19 (C6), 57.99 (C23), 47.18 (C27), 46.04 (C13), 41.06 (C29), 32.49 (C24), 29.66 (C26), 25.64 (C28), 19.55 (C25).

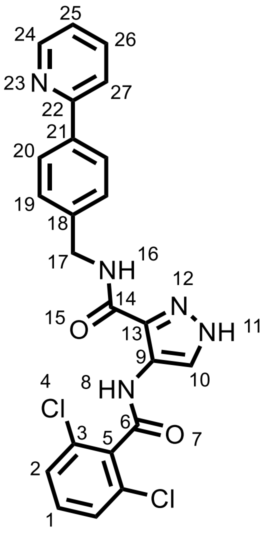

4-(2,6-dichlorobenzamido)-N-(4-(pyridin-2-yl)benzyl)-1H-pyrazole-3-carboxamide (40)

The product was synthesised following general procedure **7**, using 4-(2,6-dichlorobenzamido)-*1H*-pyrazole-3-carboxylic acid (40 mg, 0.13 mmol) and [4-(2-pyridyl)phenyl]methanamine (27 mg, 0.15 mmol). The residue was purified by column chromatography (40 → 70% EtOAc in cyclohexane) and concentrated *in vacuo* to afford the title compound (36 mg, 58%) as a white solid. LCMS purity >95%, ret. time 1.34 mins. HRMS (ESI +ve): $C_{23}H_{18}Cl_2N_5O_2$ $[M+H]^+$: 466.0837 (Found: 466.0834). ^{1}H NMR (600 MHz, DMSO-d_6) δ 13.44 (s, 1H, H11), 10.13 (s, 1H, H8), 9.12 (s, 1H, H16), 8.66 – 8.63 (m, 1H, H24), 8.38 (s, 1H, H10), 8.03 (d, *J* = 8.3 Hz, 2H, H20), 7.94 – 7.91 (m, 1H, H27), 7.87 (td, 1H, H26), 7.59 – 7.55 (m, 2H, H2), 7.53 – 7.50 (m, 1H, H1), 7.41 (d, *J* = 8.3 Hz, 2H, H19), 7.33 (ddd, *J* = 7.4, 4.8, 1.1 Hz, 1H, H25), 4.47 (d, *J* = 6.0 Hz, 2H, H17). ^{13}C NMR (151 MHz, DMSO-d_6) δ 163.88 (C14), 160.97 (C6), 156.30 (C22), 149.96 (C24), 140.87 (C18),137.66 (C26), 135.87 (C21), 133.41 (C9), 132.32 (C5), 131.73 (C3), 128.87 (C2), 128.13 (C19), 126.92 (C20), 122.94 (C25), 121.92 (C13), 121.33 (C10), 120.54 (C27), 41.99 (C17).

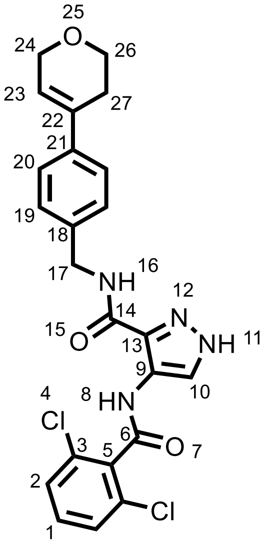

4-(2,6-dichlorobenzamido)-N-(4-(3,6-dihydro-2H-pyran-4-yl)benzyl)-1H-pyrazole-3-carboxamide (41)

The product was synthesised following general procedure **7**, using 4-(2,6-dichlorobenzamido)-*1H*-pyrazole-3-carboxylic acid (20 mg, 0.06 mmol) and [4-(3,6-dihydro-2H-pyran-4-yl)phenyl]methanamine (27 mg, 0.15 mmol). The residue was purified by column chromatography (20 → 60% EtOAc in cyclohexane) and concentrated *in vacuo* to afford the title compound (1.8 mg, 6%) as a white solid. LCMS purity >95%, ret. time 1.48 mins. HRMS (ESI +ve): C23H21Cl2N4O3 [M+H]+: 471.0963 (Found: 471.0964). ^{1}H NMR (600 MHz, DMSO-d$_6$) δ 13.42 (s, 1H, H1), 10.12 (s, 1H, H8), 9.05 (t, *J* = 6.4 Hz, 1H, H16), 8.37 (s, 1H, H10), 7.59 – 7.56 (m, 2H, H2), 7.53 – 7.50 (m, 1H, H1), 7.38 (d, *J* = 8.3 Hz, 2H, H20), 7.27 (d, *J* = 8.3 Hz, 2H, H19), 6.22 – 6.18 (m, 1H, H23), 4.39 (d, *J* = 6.3 Hz, 2H, H17), 4.23 – 4.19 (m, 2H, H24), 3.80 (t, *J* = 5.5 Hz, 2H, H26), 2.44 – 2.39 (m, 2H, H27). ^{13}C NMR (151 MHz, DMSO-d$_6$) δ 163.72 (C14), 160.86 (C6), 138.91 (C18), 138.68 (C21), 135.86 (C5), 133.43 (C9), 133.34 (C22), 132.33 (C1), 131.73 (C3), 128.87 (C2), 127.89 (C19), 124.82 (C20), 122.82 (C23), 121.90 (C13), 121.29 (C10), 65.51 (C24), 64.09 (C26), 41.91 (C17), 26.92 (C27).

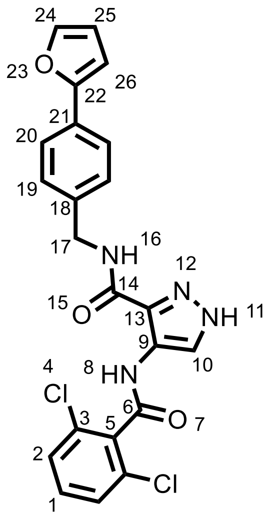

4-(2,6-dichlorobenzamido)-N-(4-(furan-2-yl)benzyl)-*1H*-pyrazole-3-carboxamide (42)

The product was synthesised following general procedure **7**, using 4-(2,6-dichlorobenzamido)-*1H*-pyrazole-3-carboxylic acid (40 mg, 0.13 mmol) (4-(furan-2-yl)phenyl)methanamine hydrochloride (25 mg, 0.15 mmol). The residue was purified by column chromatography (20 → 60% EtOAc in cyclohexane) and concentrated *in vacuo* to afford the title compound (6 mg, 10%) as a white solid. LCMS purity >95%, ret. time 1.59 mins. HRMS (ESI +ve): $C_{22}H_{17}Cl_2N_4O_3$ $[M+H]^+$: 455.0650 (Found: 455.0650). ^{1}H NMR (600 MHz, DMSO-d$_6$) δ 13.43 (s, 1H, H11), 10.13 (s, 1H, H8), 9.08 (t, *J* = 6.3 Hz, 1H, H16), 8.38 (s, 1H, H10), 7.72 (d, *J* = 1.1 Hz, 1H, H24), 7.64 (d, *J* = 8.3 Hz, 2H, H20), 7.59 – 7.56 (m, 2H, H2), 7.53 – 7.50 (m, 1H, H1), 7.35 (d, *J* = 8.3 Hz, 2H, H19), 6.89 (dd, *J* = 3.4, 0.7 Hz, 1H, H26), 6.58 (dd, *J* = 3.4, 1.8 Hz, 1H, H25), 4.42 (d, *J* = 6.3 Hz, 2H, H17). ^{13}C NMR (151 MHz, DMSO-d$_6$) δ 163.76 (C14), 160.88 (C6), 153.43 (C22), 143.19 (C24), 139.20 (C18), 135.86 (C5), 133.34 (C9), 132.32 (C1), 131.73 (C3), 129.44 (C21), 128.87 (C2), 128.32 (C19), 123.84 (C23), 121.91 (C13), 121.32 (C10), 112.48 (C25), 106.01 (C26), 41.98 (C17).

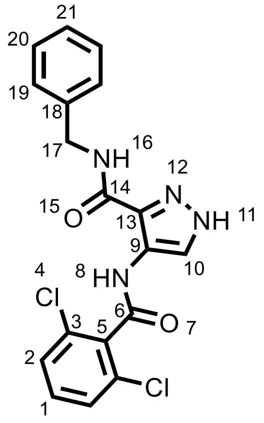

N-benzyl-3-[(2,6-dichlorobenzoyl)amino]-1H-pyrazole-4-carboxamide (44)

The product was synthesised by combining 3-amino-N-benzyl-1H-pyrazole-4-carboxamide (28.00 mg, 0.13 mmol), triethylamine (0.05 mL, 0.36 mmol) and 2,6-dichlorobenzoyl chloride (0.02 mL, 0.12 mmol) in DCM (1 mL, 0.12 M). The reaction was stirred at RT for two hours and then the product was diluted into water. The aqueous layer was extracted with DCM (x3) and the combined organics were washed with brine (x3). The product was purified by column chromatography (10 → 50% EtOAc in cyclohexane) and the desired fractions were concentrated *in vacuo* to afford the title compound (16 mg, 32%) as a white solid. ^{1}H NMR (600 MHz, $CDCl_3$) δ 9.91 (s, 1H, H8), 8.55 (s, 1H, H10), 7.39 – 7.30 (m, 8H, H1, H2, H19, H20, H21), 4.61 (d, *J* = 6.0 Hz, 2H, H17).

S2. ^{1}HNMR and ^{13}CNMR spectra of key compounds

^{1}HNMR and ^{13}CNMR spectra for compound **1**

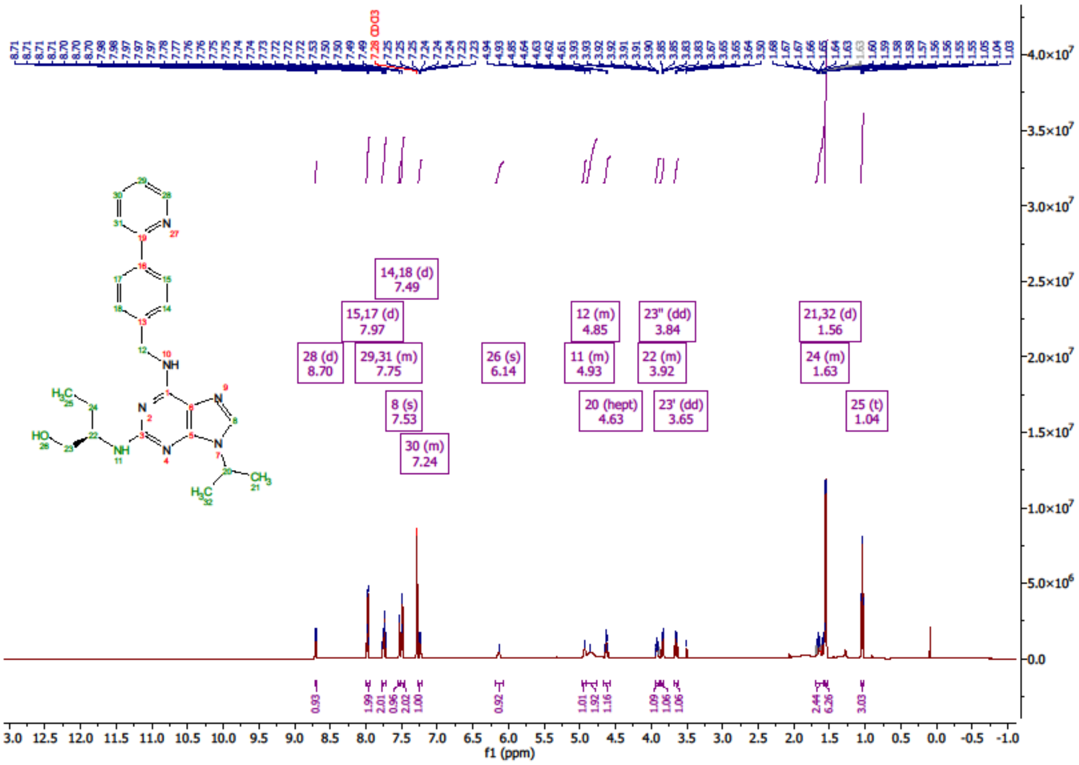

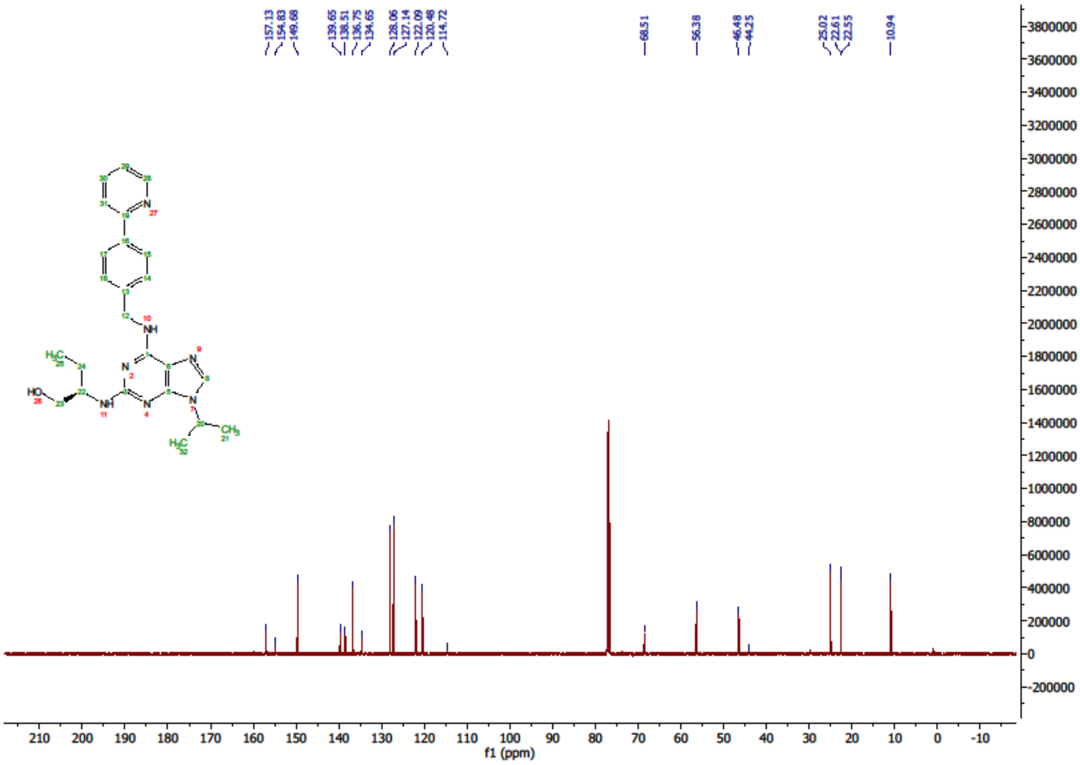

^{1}HNMR and ^{13}CNMR spectra for compound **12**

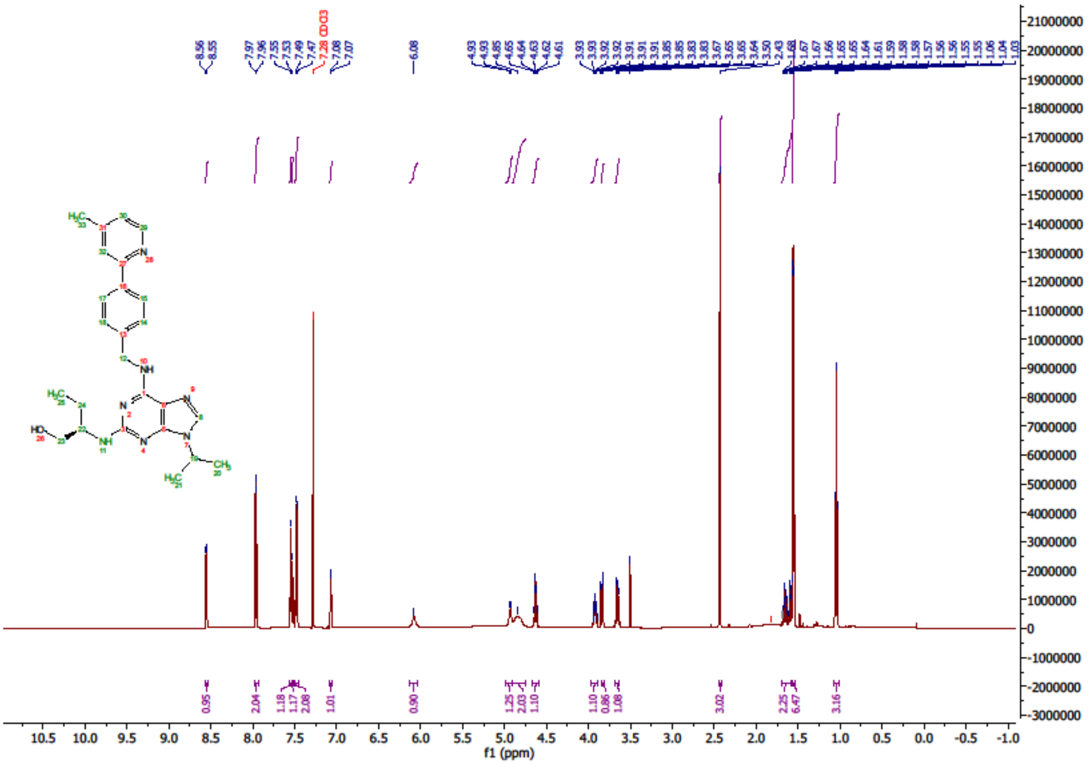

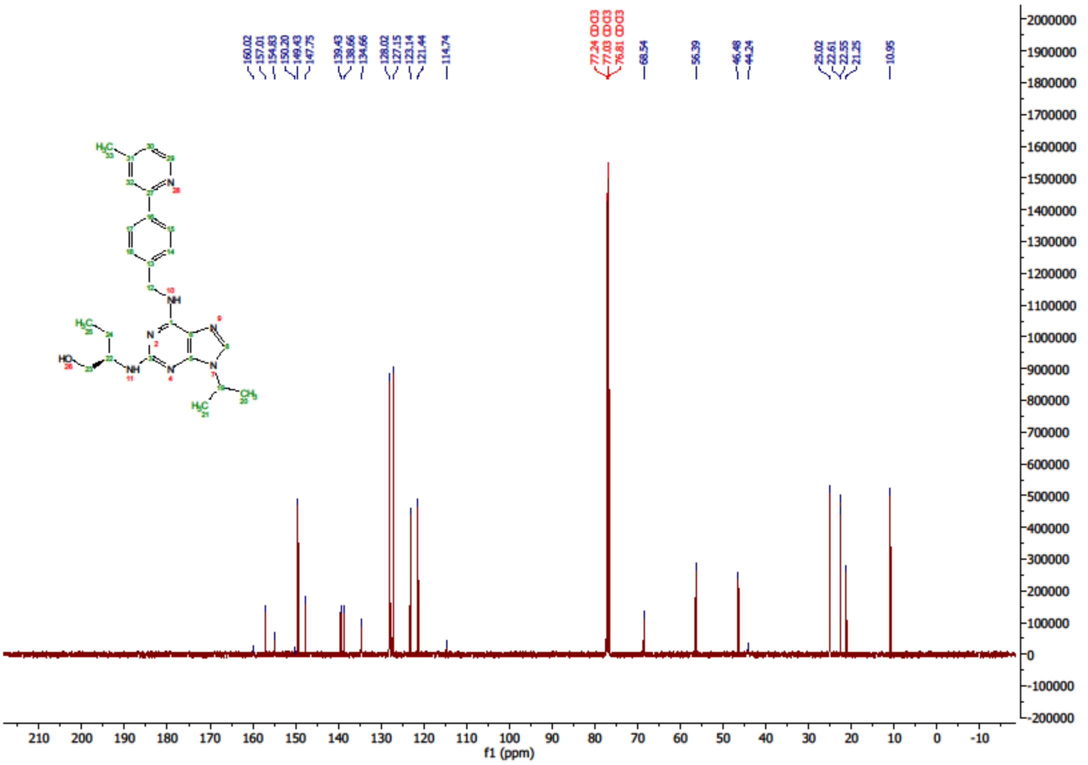

^{1}HNMR and ^{13}CNMR spectra for compound **15**

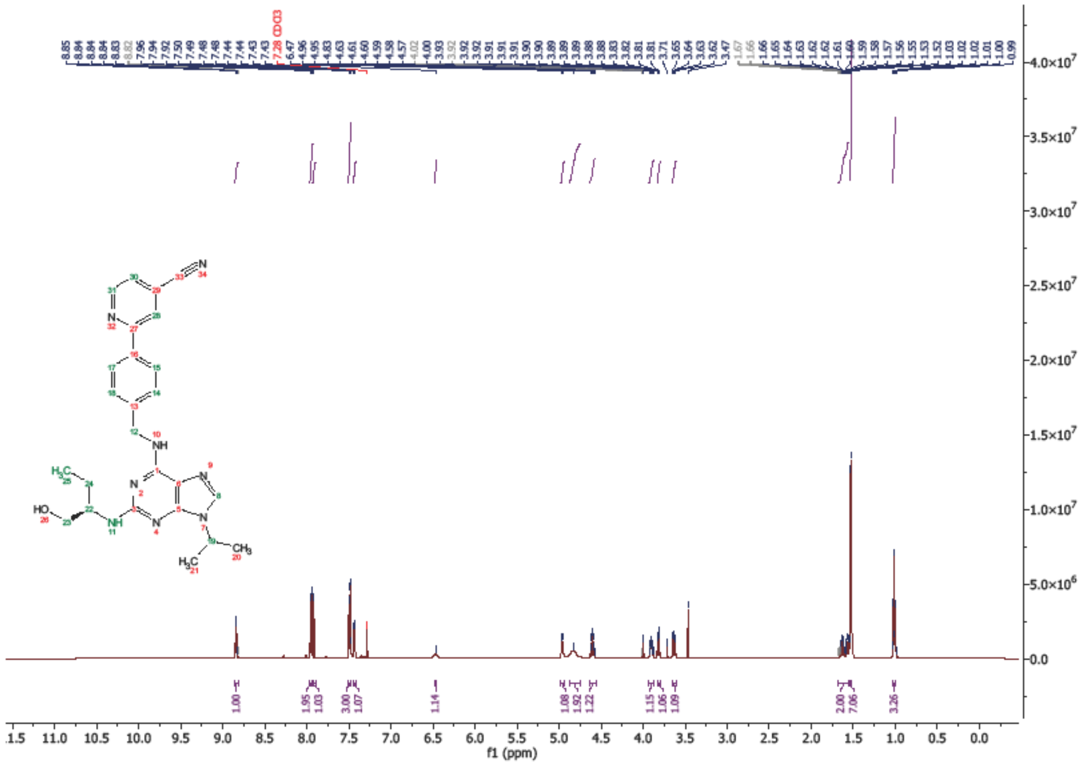

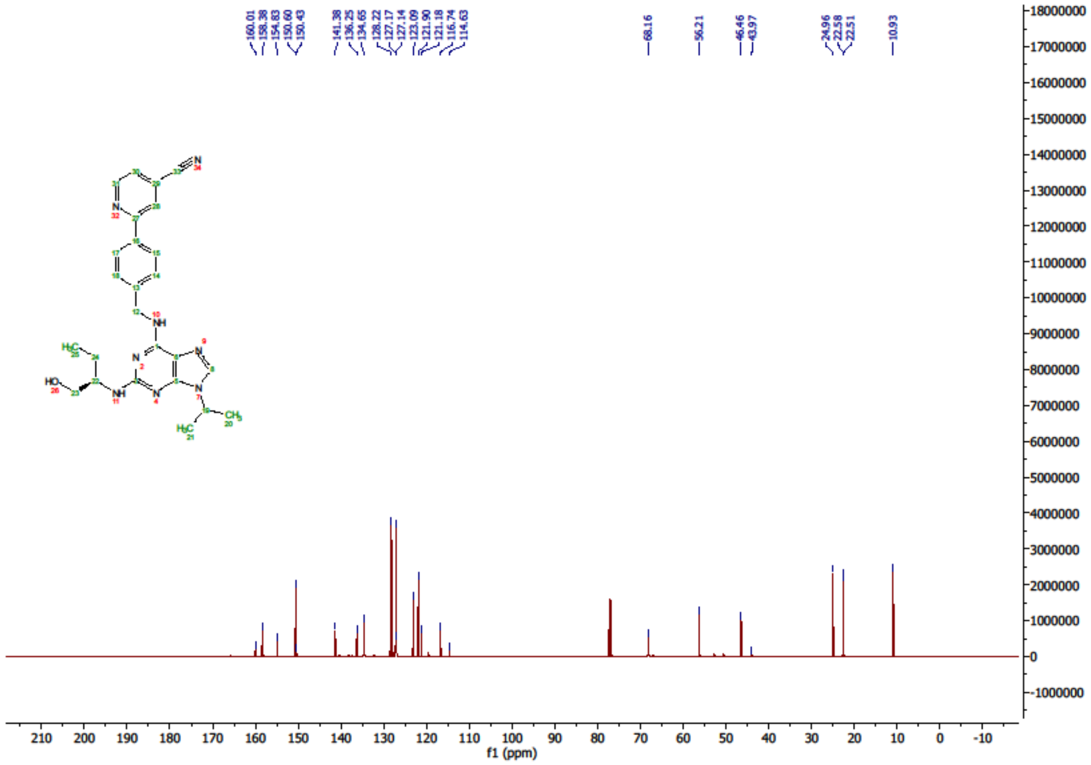

^{1}HNMR and ^{13}CNMR spectra for compound **21**

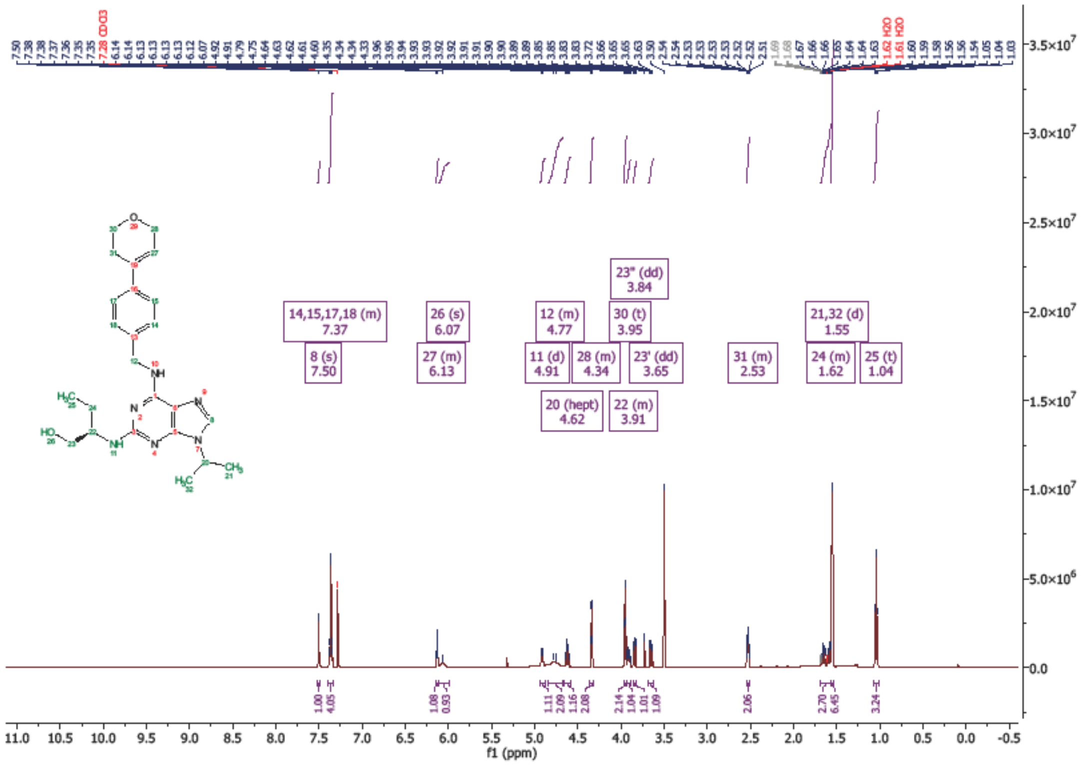

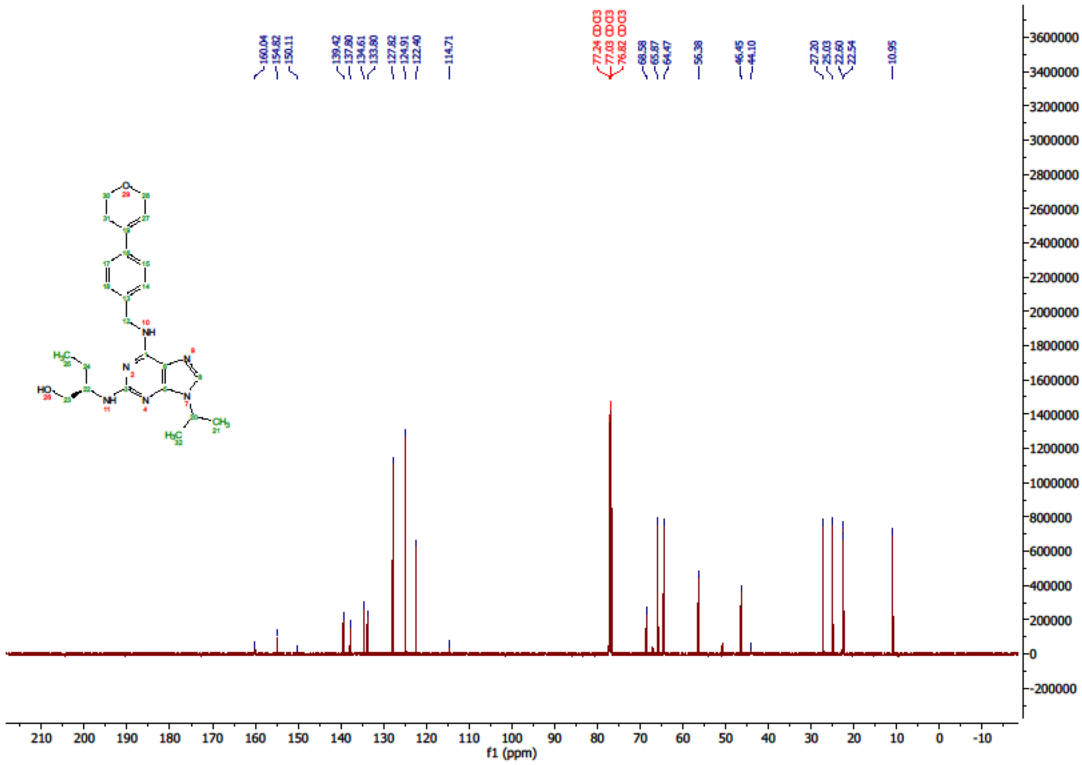

^{1}HNMR and ^{13}CNMR spectra for compound **29**

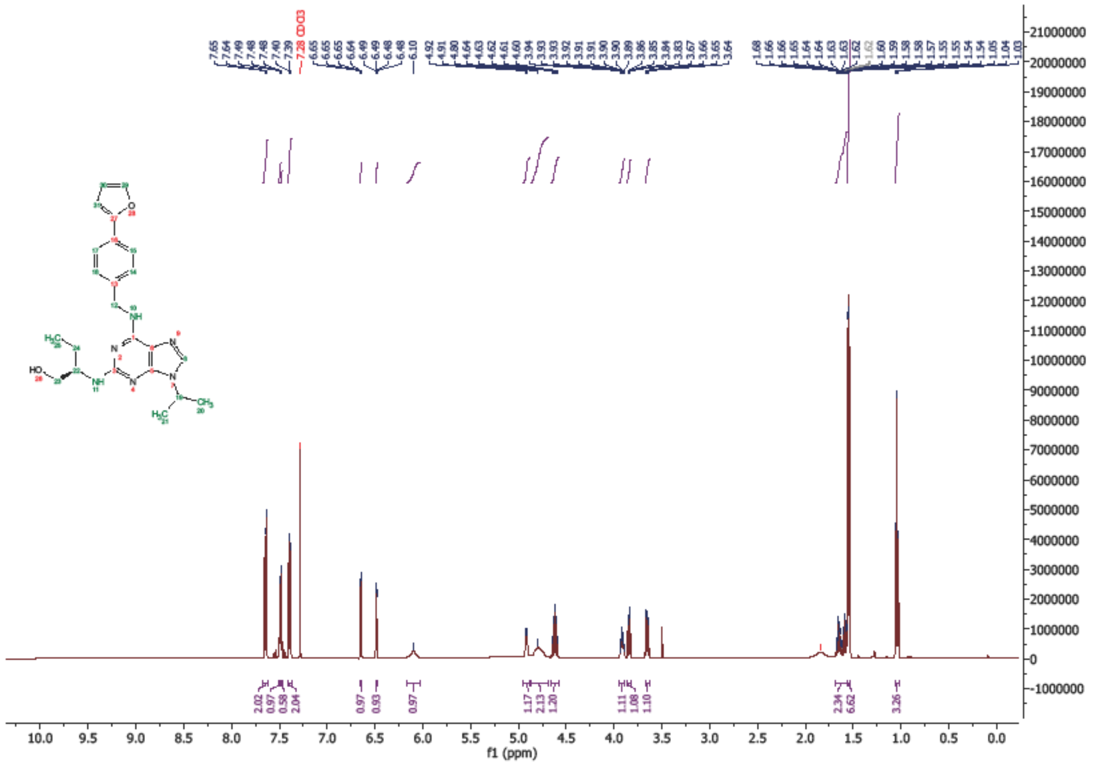

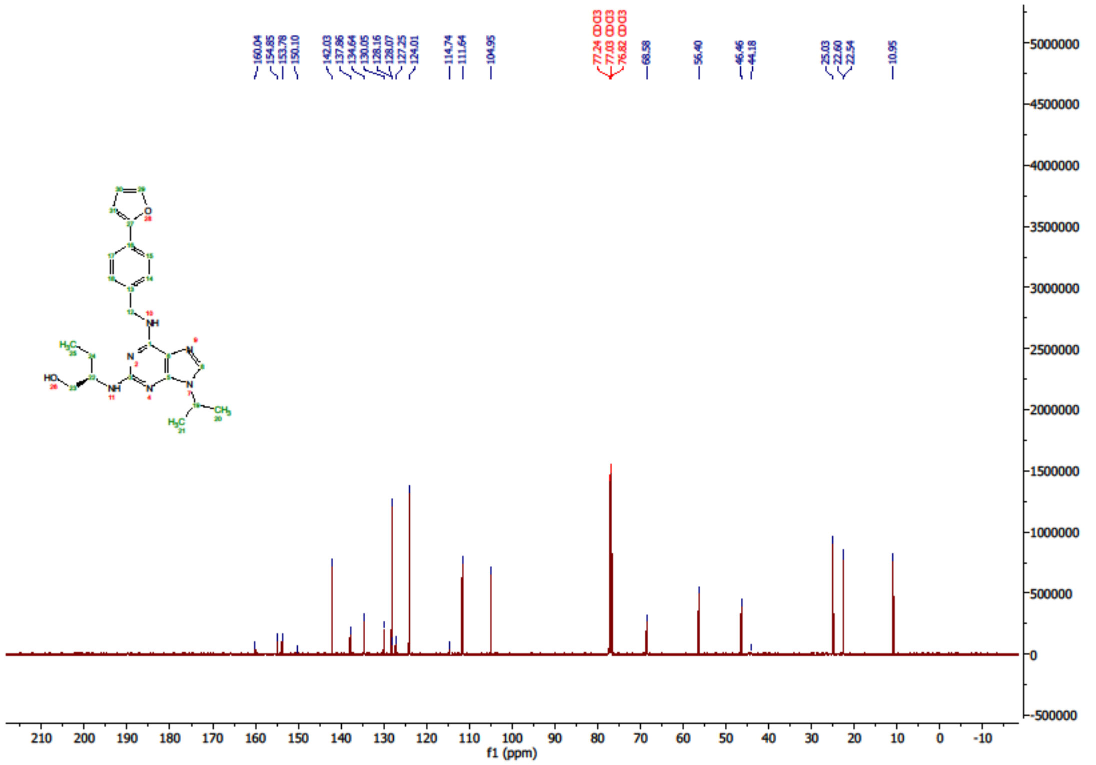

^{1}HNMR and ^{13}CNMR spectra for compound **36**

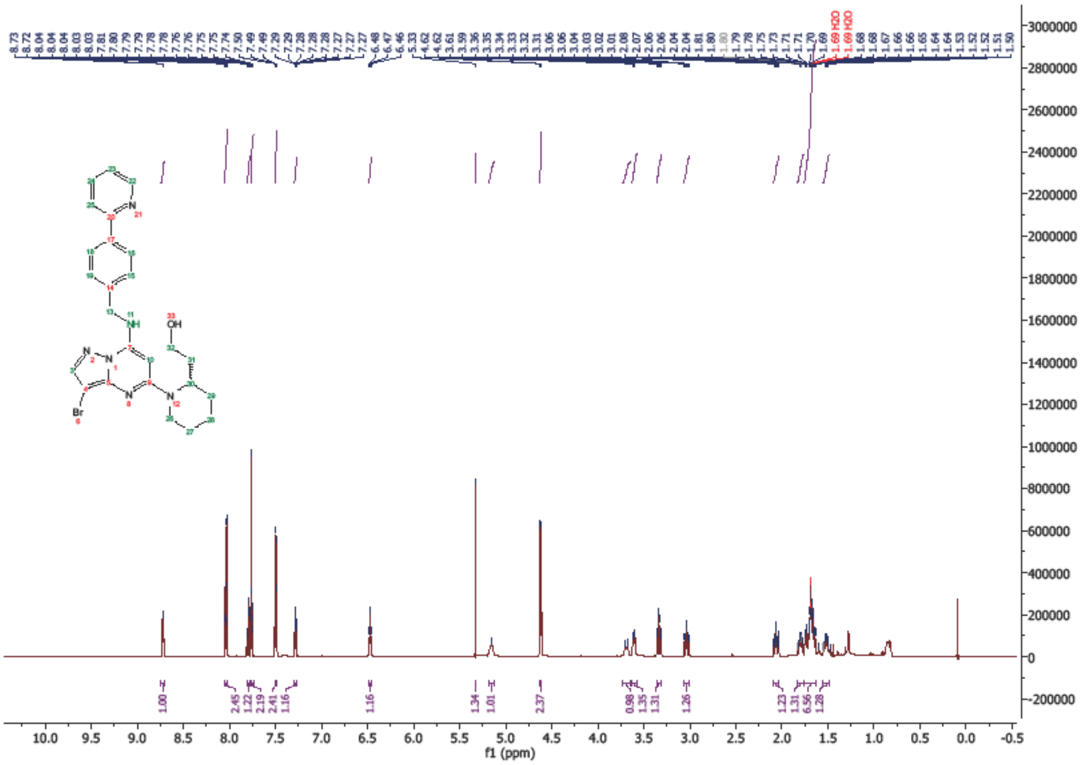

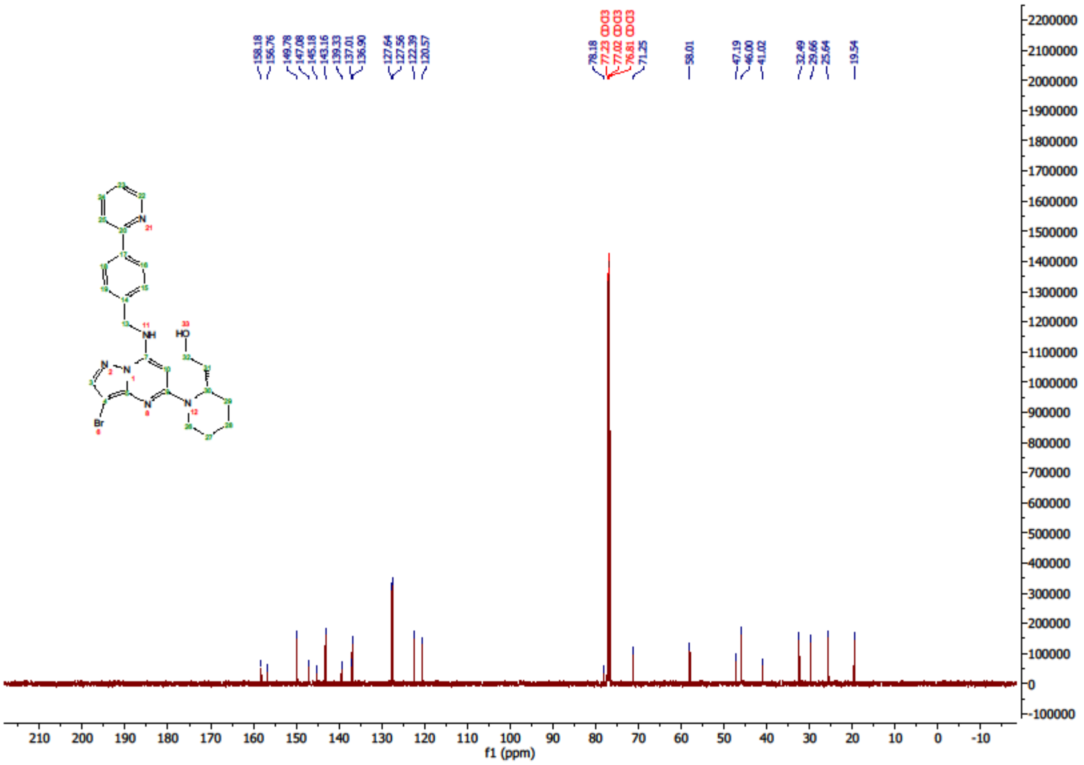

^{1}HNMR and ^{13}CNMR spectra for compound **37**

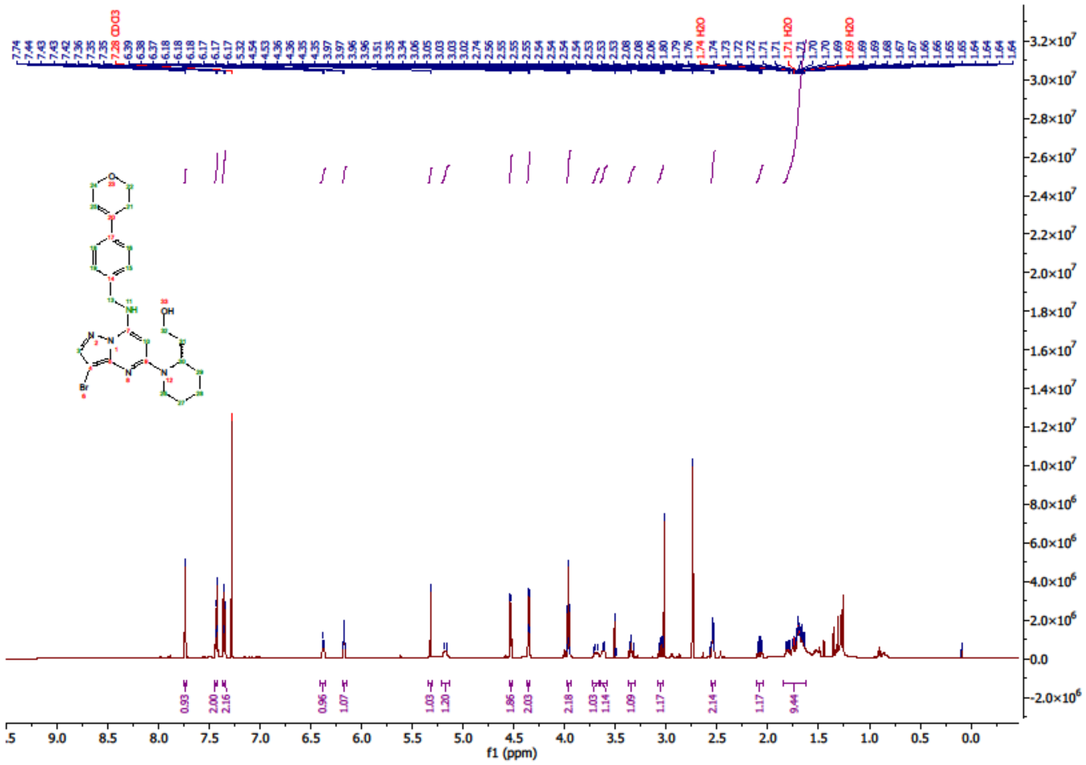

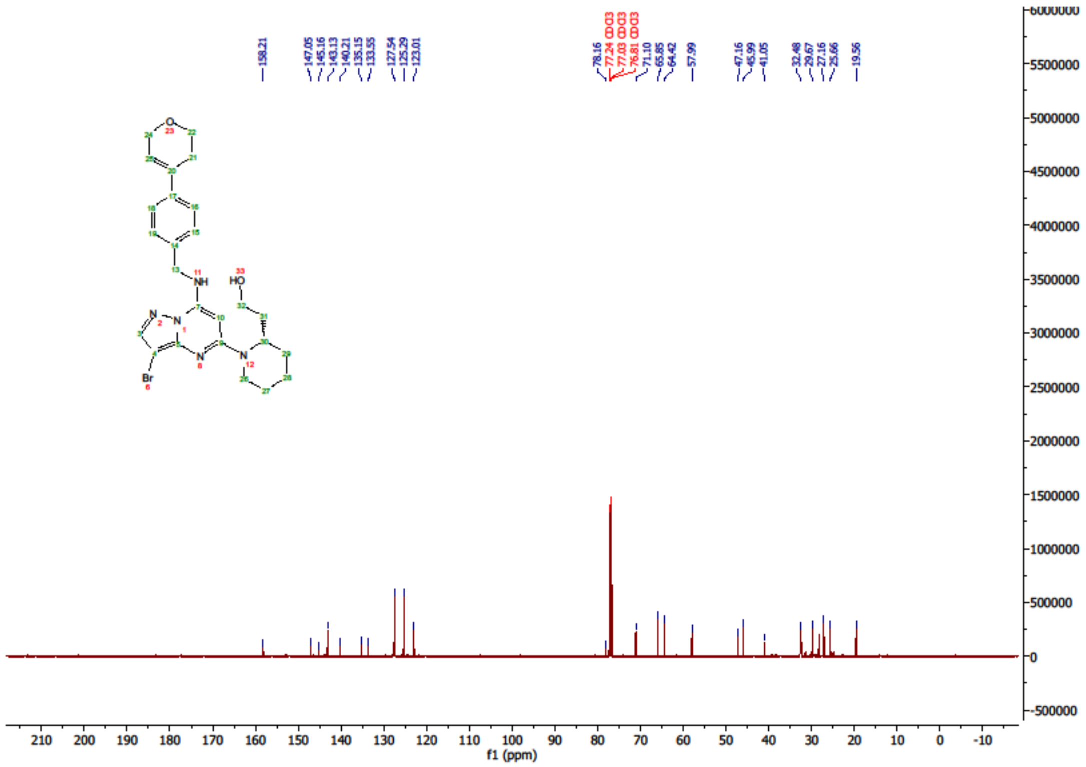

^{1}HNMR and ^{13}CNMR spectra for compound **39**

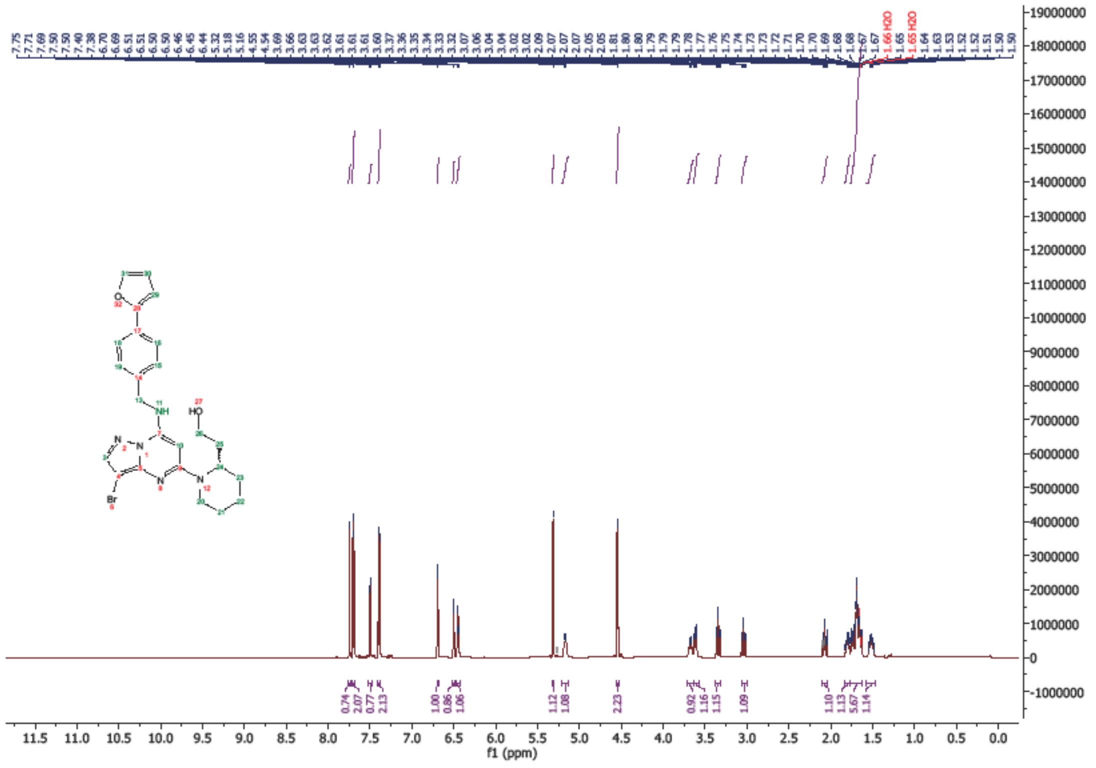

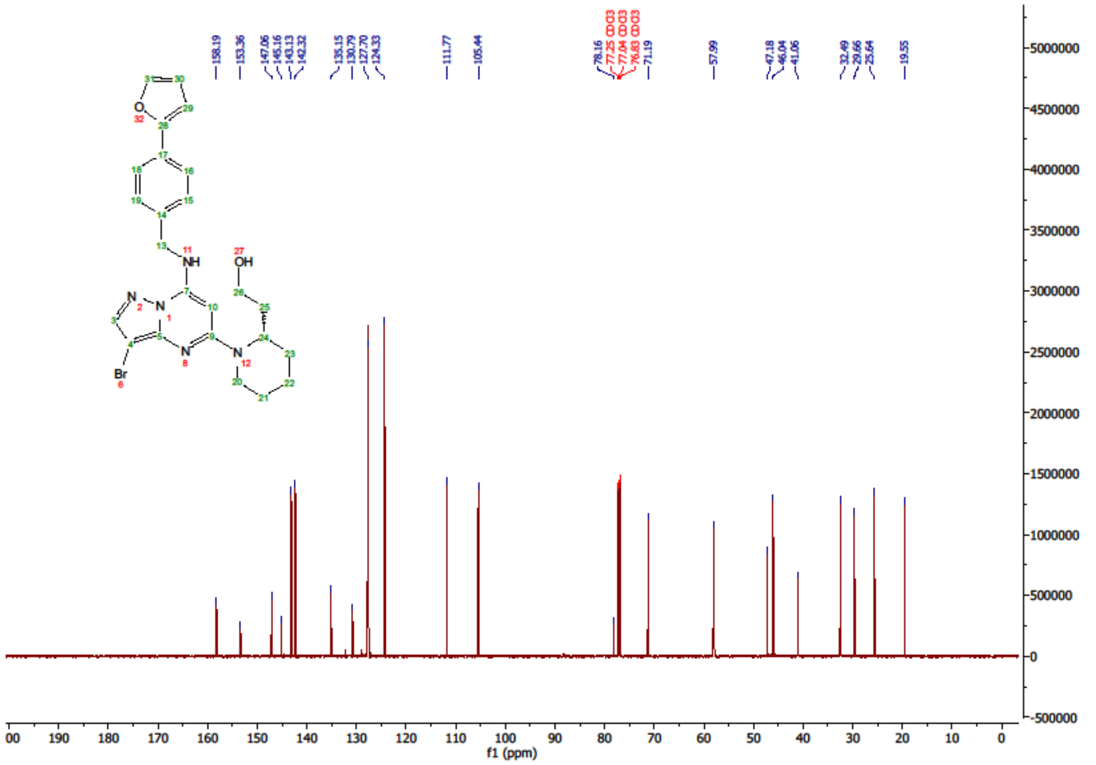

^{1}HNMR and ^{13}CNMR spectra for compound **40**

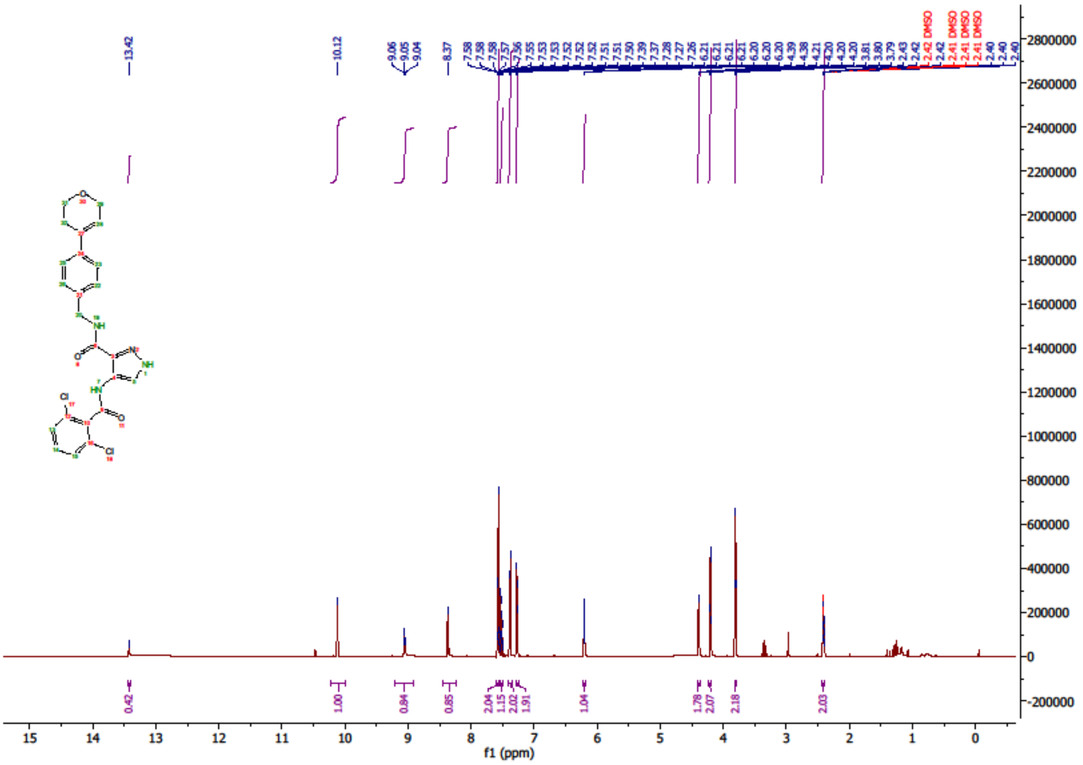

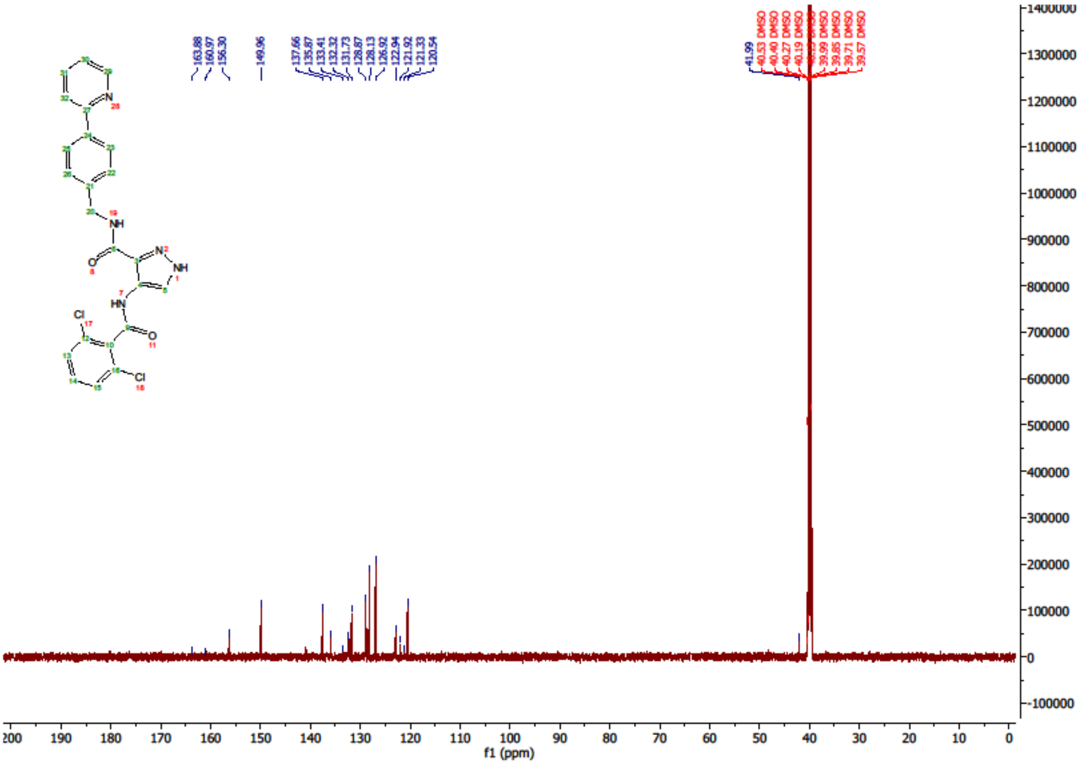

^{1}HNMR and ^{13}CNMR spectra for compound **41**

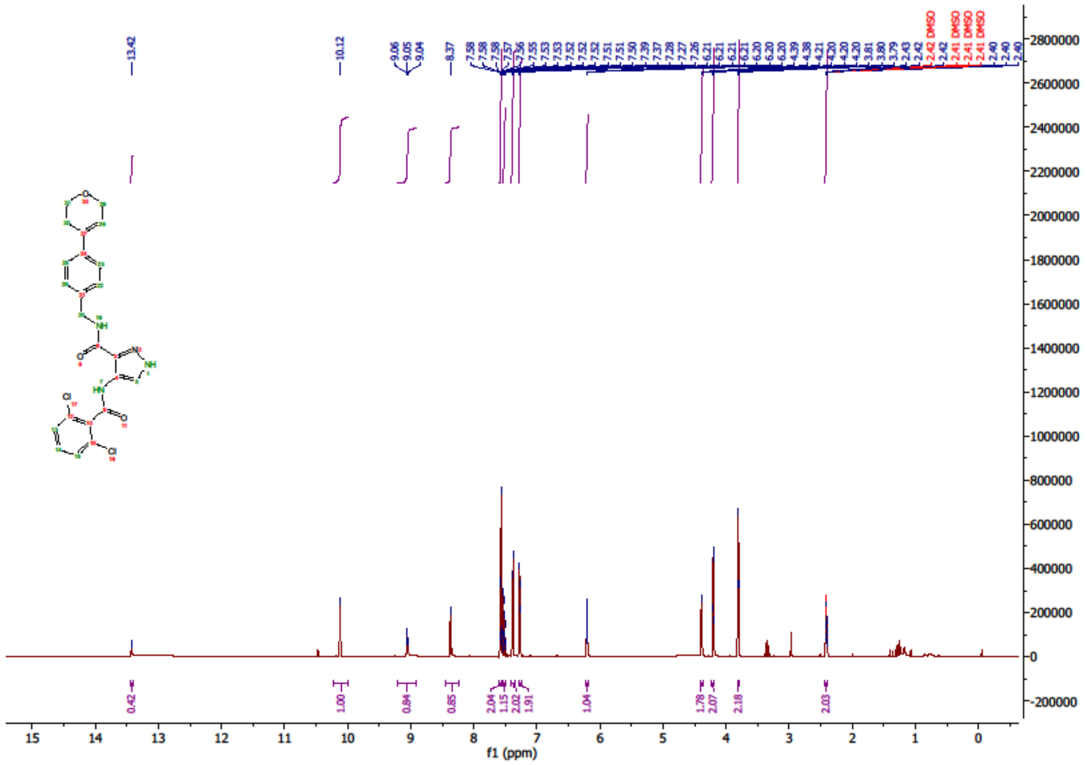

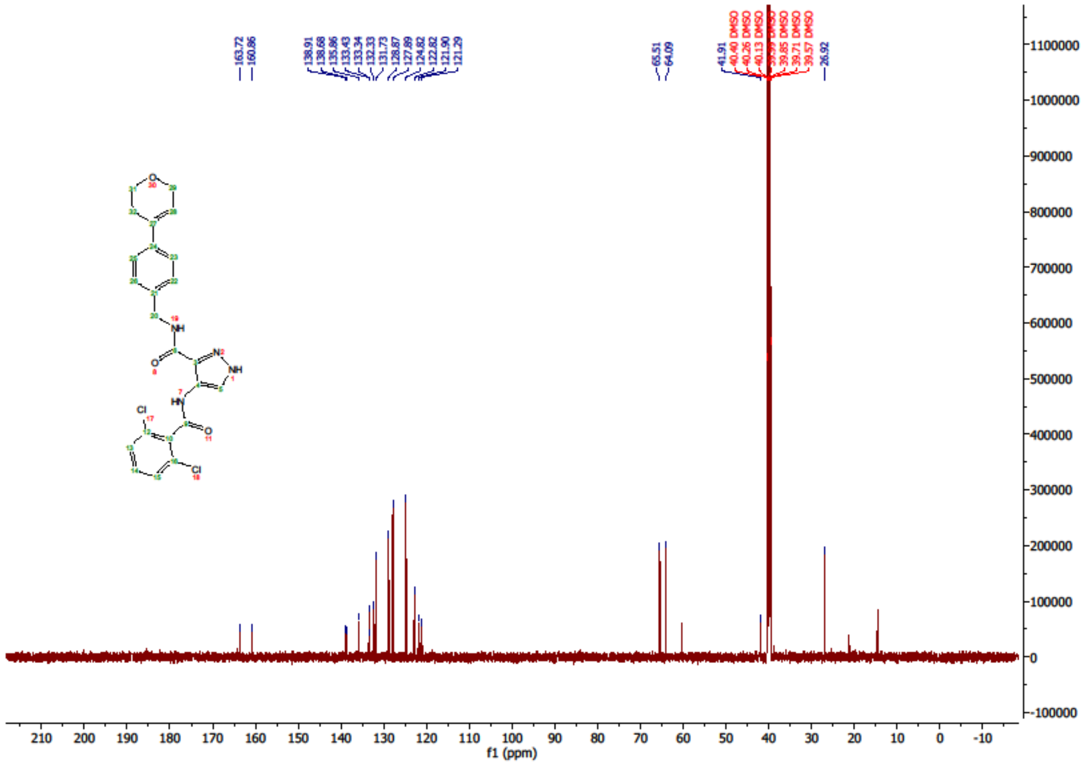

^{1}HNMR and ^{13}CNMR spectra for compound **42**

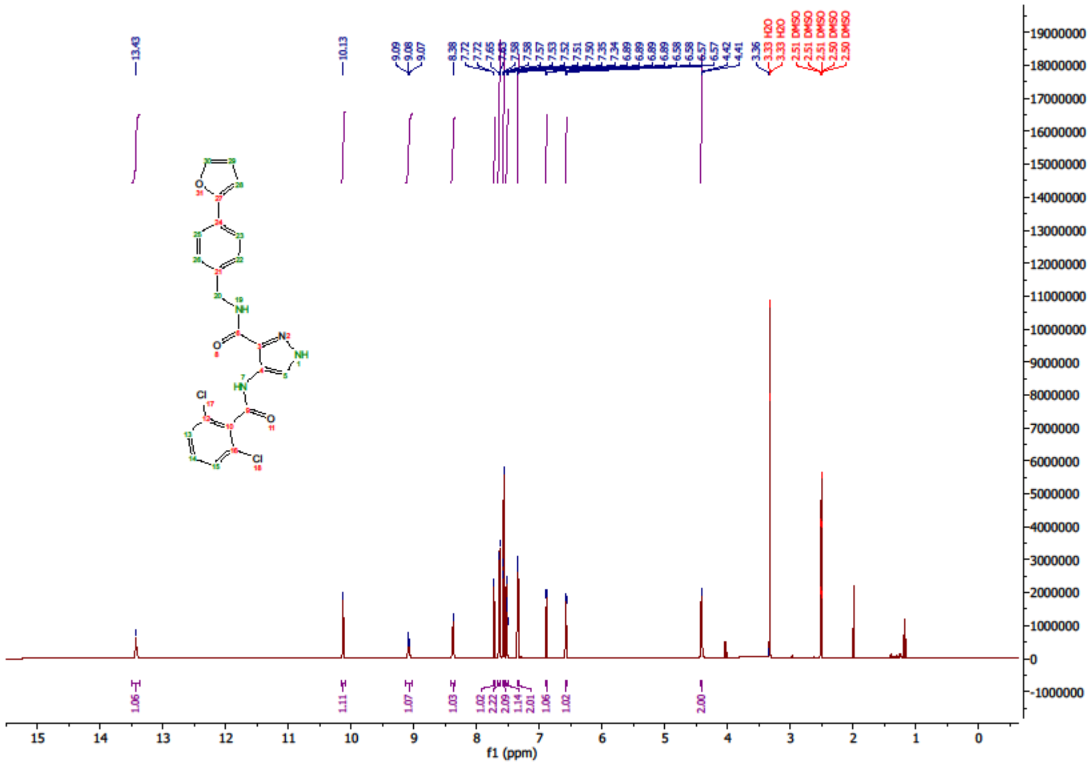

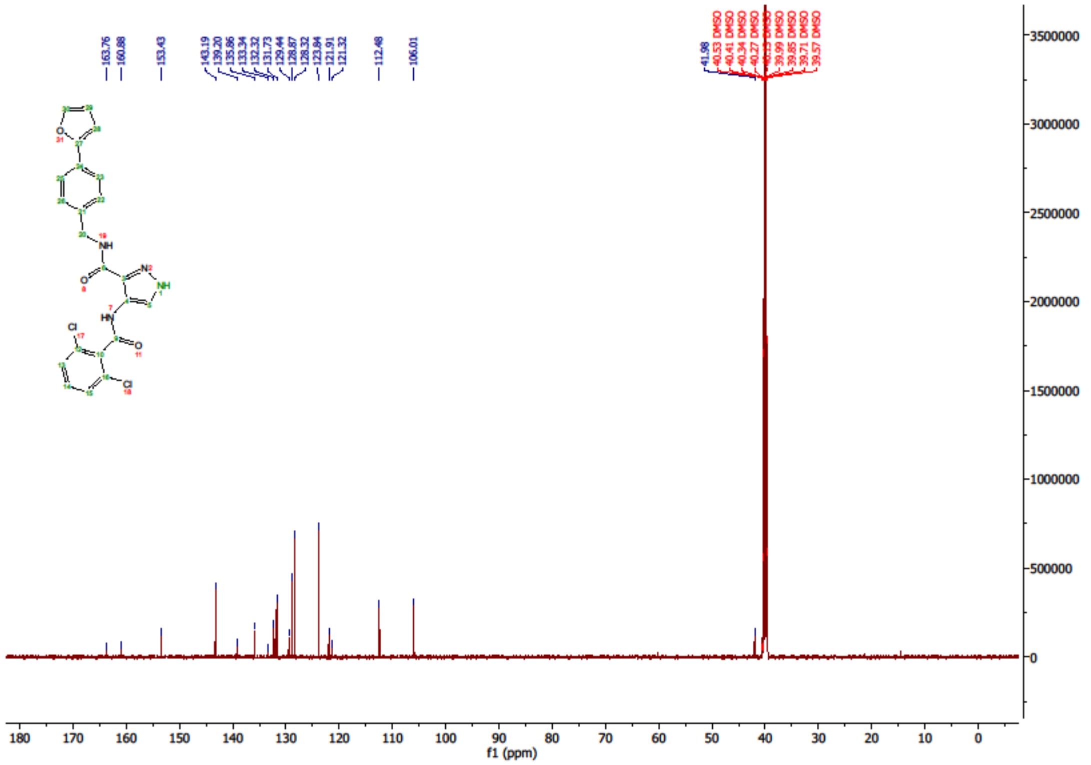

S3. Western blotting data

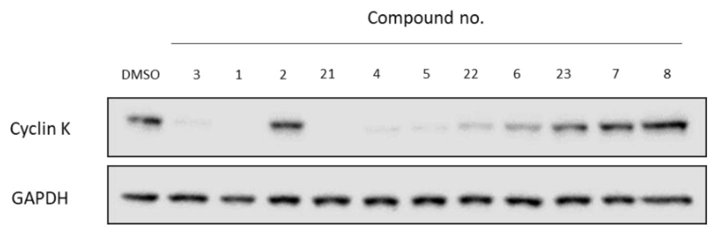

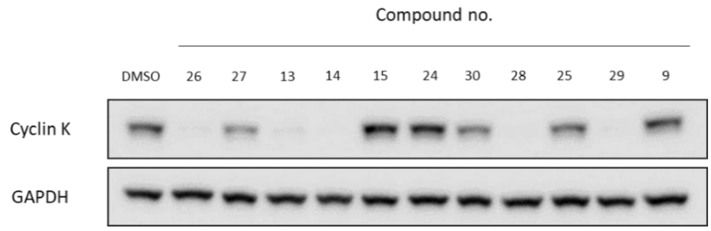

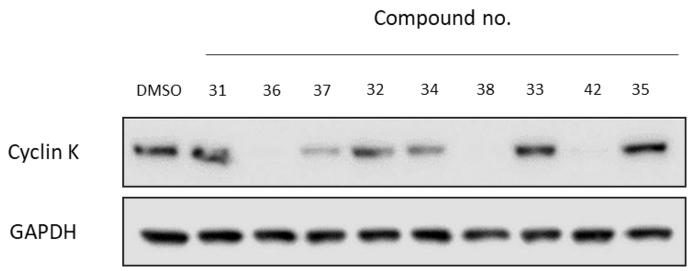

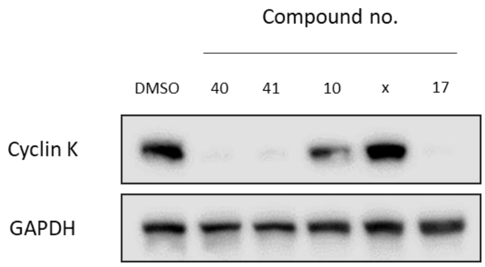

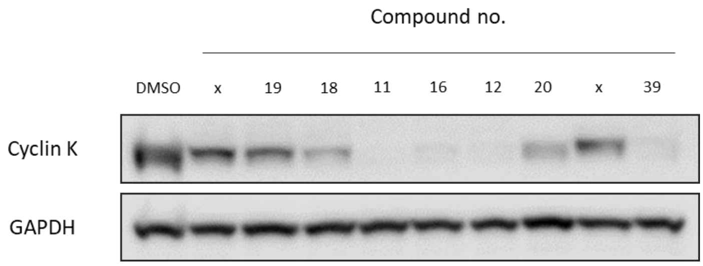

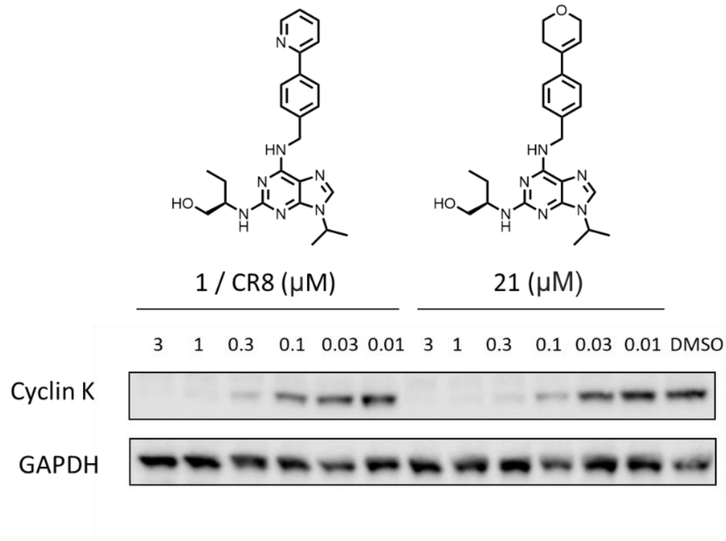

1 / CR8 (µM)
21 (µM)
3 1 0.3 0.1 0.03 0.01 3 1 0.3 0.1 0.03 0.01 DMSO
Cyclin K
GAPDH

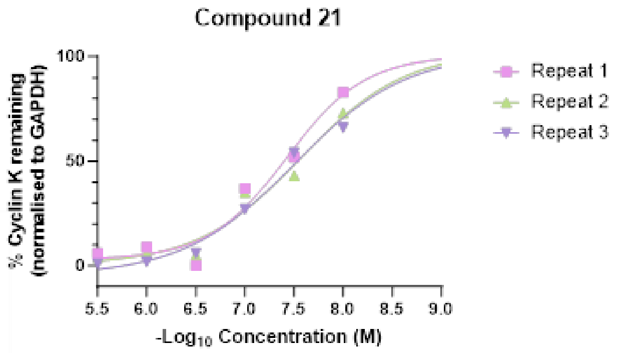

Compound 21
% Cyclin K remaining (normalised to GAPDH)
-Log10 Concentration (M)
Repeat 1
Repeat 2
Repeat 3

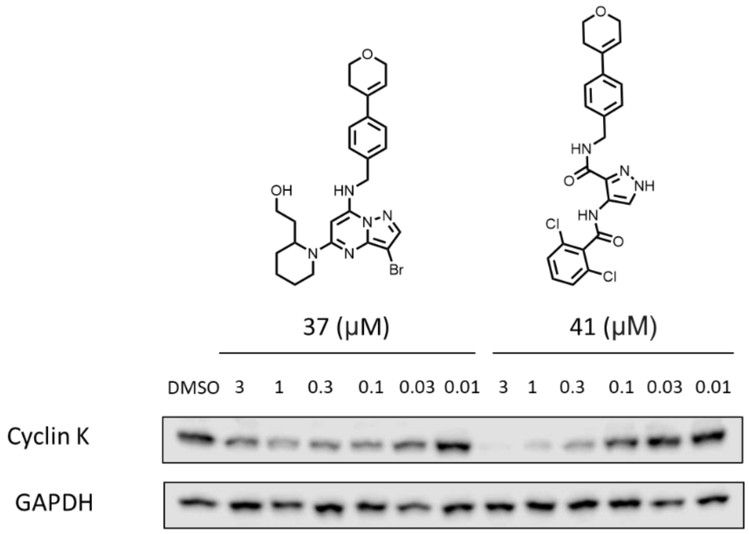

37 (µM)
41 (µM)
DMSO 3 1 0.3 0.1 0.03 0.01 3 1 0.3 0.1 0.03 0.01
Cyclin K
GAPDH

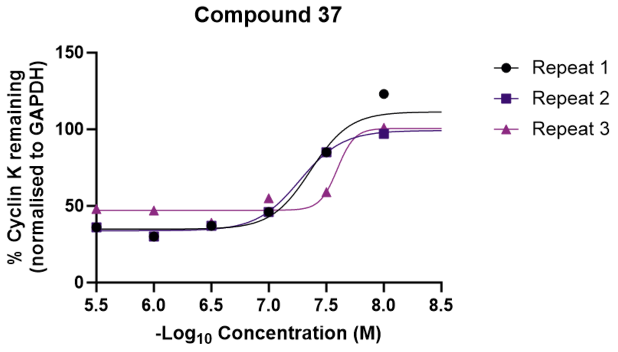

Compound 37
% Cyclin K remaining (normalised to GAPDH)
-Log10 Concentration (M)
Repeat 1
Repeat 2
Repeat 3

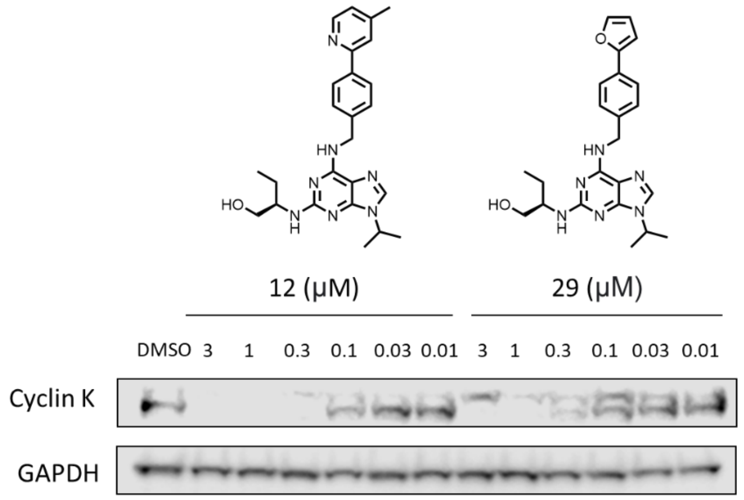

12 (µM)
29 (µM)
DMSO 3 1 0.3 0.1 0.03 0.01 3 1 0.3 0.1 0.03 0.01
Cyclin K
GAPDH

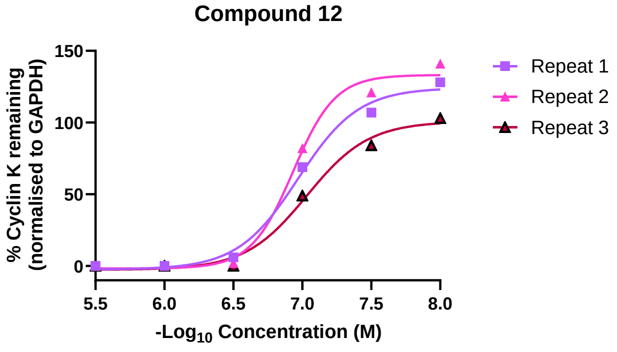

Compound 12
% Cyclin K remaining (normalised to GAPDH)
-Log10 Concentration (M)
Repeat 1
Repeat 2
Repeat 3

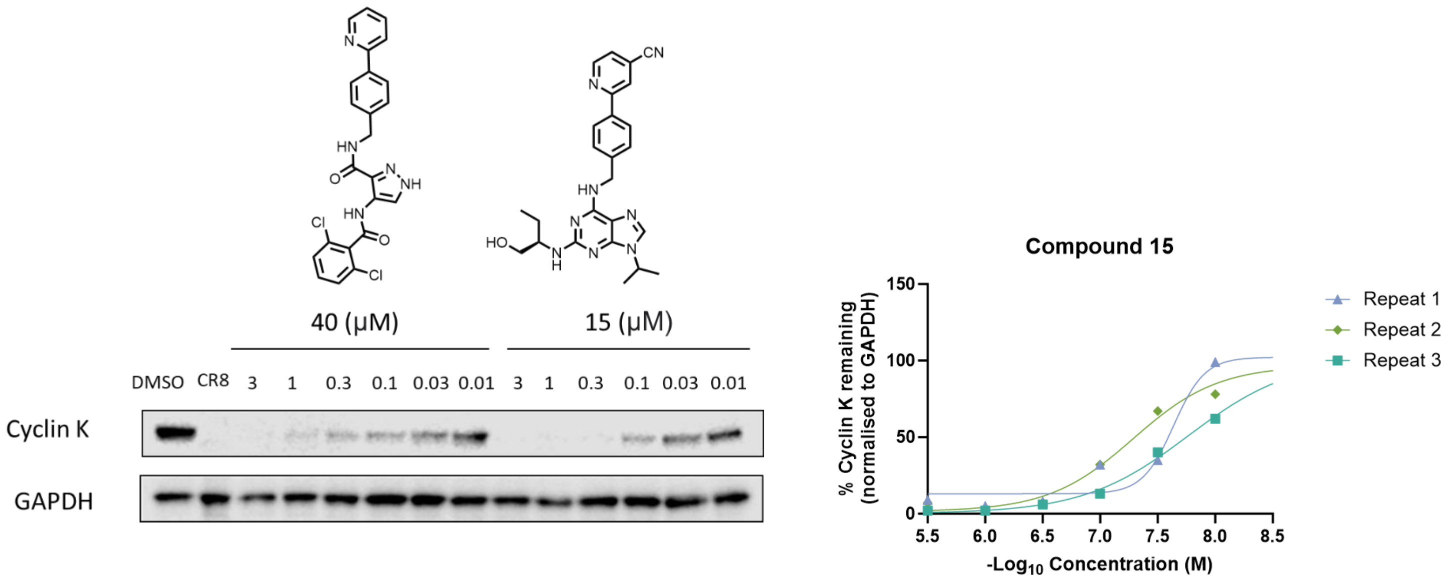
40 (μM)
15 (μM)
DMSO CR8 3 1 0.3 0.1 0.03 0.01 3 1 0.3 0.1 0.03 0.01
Cyclin K
GAPDH
Compound 15
% Cyclin K remaining (normalised to GAPDH)
-Log10 Concentration (M)
Repeat 1
Repeat 2
Repeat 3

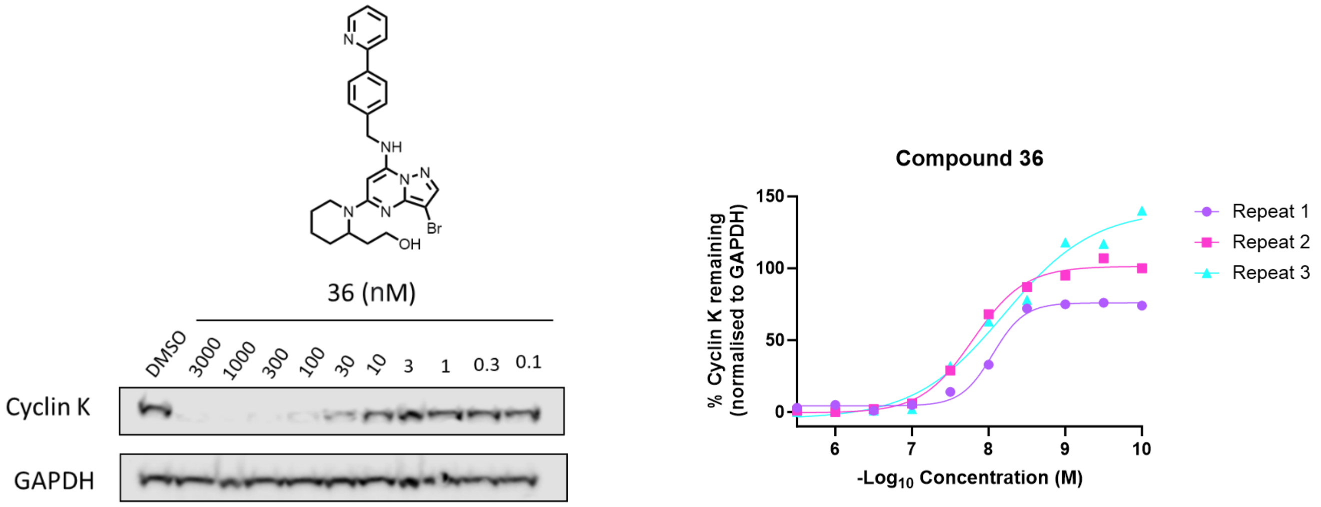
36 (nM)
DMSO 3000 1000 300 100 30 10 3 1 0.3 0.1
Cyclin K
GAPDH
Compound 36
% Cyclin K remaining (normalised to GAPDH)
-Log10 Concentration (M)
Repeat 1
Repeat 2
Repeat 3

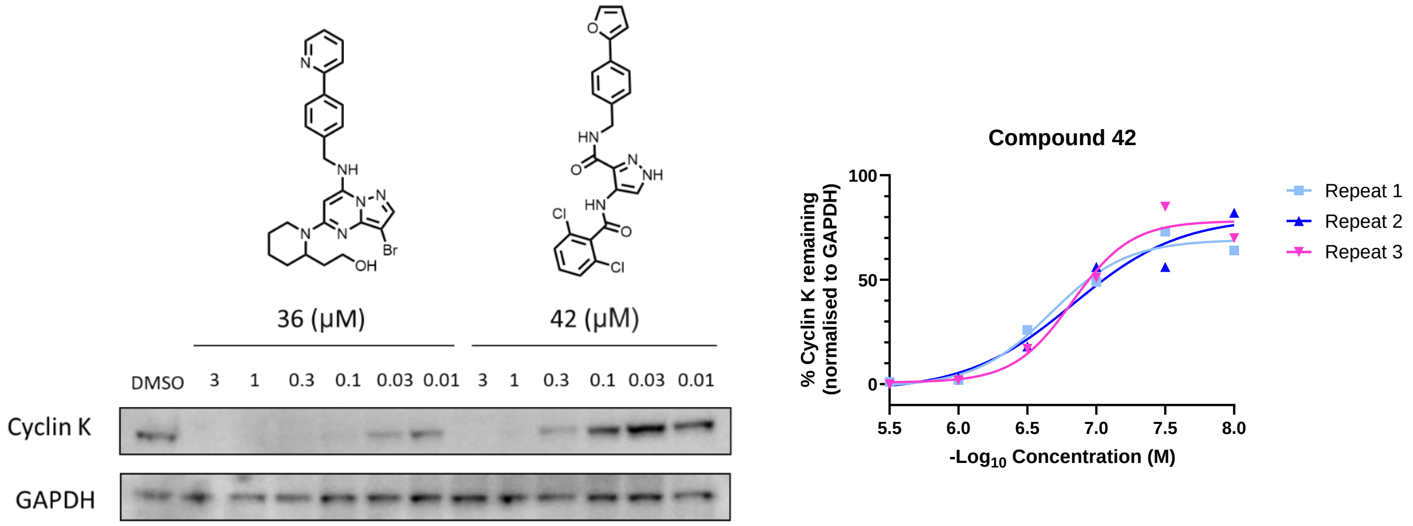
36 (μM)
42 (μM)
DMSO 3 1 0.3 0.1 0.03 0.01 3 1 0.3 0.1 0.03 0.01
Cyclin K
GAPDH
Compound 42
% Cyclin K remaining (normalised to GAPDH)
-Log10 Concentration (M)
Repeat 1
Repeat 2
Repeat 3

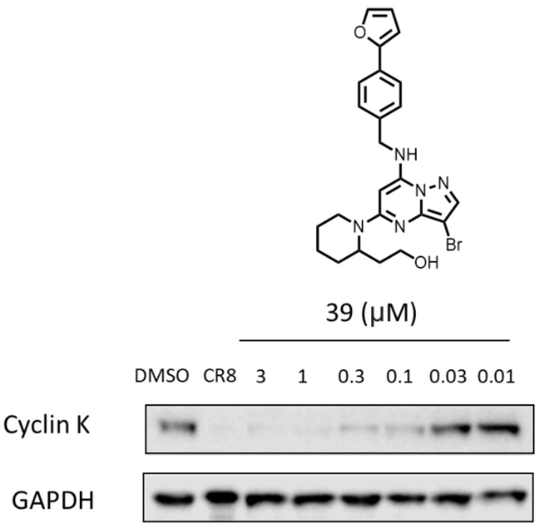

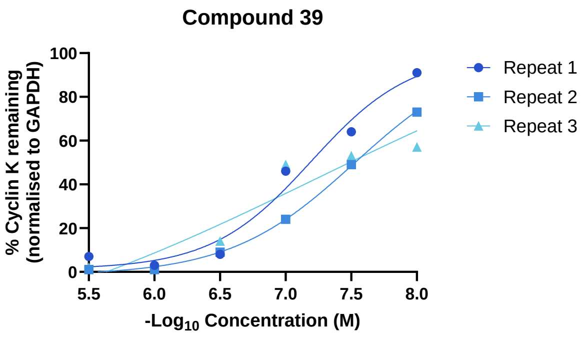

S4. pDC$_{50}$ values

Individual pDC$_{50}$s with standard error.

	pDC50				
**compound no.**	**repeat 1**	**repeat 2**	**repeat 3**	**average**	**standard deviation**
1	6.996	7.663	7.800	7.487	0.351
21	7.482	7.437	7.555	7.491	0.049
29	6.680	6.933	6.568	6.727	0.153
12	6.965	6.935	7.029	6.977	0.039
15	7.859	7.649	7.756	7.755	0.086
36	8.055	7.807	8.208	8.023	0.166
37	7.377	7.276	7.569	7.407	0.121
39	7.205	7.524	6.907	7.212	0.252
40	7.943	7.615	7.576	7.711	0.165
41	6.766	7.026	6.799	6.864	0.115
42	6.837	6.686	6.819	6.780	0.067
